# Epigenetic silencing and CRISPR-mediated reactivation of tight junction protein claudin10b (CLDN10B) in renal cancer

**DOI:** 10.1186/s13148-025-01911-2

**Published:** 2025-06-16

**Authors:** Sarah Arroyo Villora, Yufen Zhao, Paula Castellanos Silva, Alba A. Hahn, Vivien Olanin, David Groll, Sandra Maurer, Vera Roetzer, Witold Szymanski, Tara Procida-Kowalski, Niklas Philipp, Aline Koch, Marek Bartkuhn, Johannes Graumann, Richard Volckmann, Jan Koster, Oliver Rossbach, Denise Salzig, Reinhard Dammann, Cornelia Sigges, Jan Halbritter, Silke Haerteis, Antje Maria Richter

**Affiliations:** 1 Institute for Genetics, Justus-Liebig-University Giessen, Heinrich-Buff Ring 58, 35390 Giessen, Germany; 2 Institute for Molecular and Cellular Anatomy, University of Regensburg, 93053 Regensburg, Germany; 3 Institute of Translational Proteomics & Core Facility Translational Proteomics, Biochemical/Pharmacological Centre, Philipps-University, 35043 Marburg, Germany; 4 Platform for Genomics and Bioinformatics, Institute for Lung Health (ILH), Justus-Liebig University, Giessen, Germany; 5 Platform for Genomics and Bioinformatics, Institute for Lung Health (ILH), German Center for Lung Research (DZL), University of Giessen and Marburg Lung Center (UGMLC), Cardio-Pulmonary Institute (CPI), Justus-Liebig University, Giessen, Germany; 6 Cell Biology and Plant Biochemistry, Institute of Plant Sciences, University of Regensburg, 93053 Regensburg, Germany; 7 Amsterdam UMC, Center for Experimental and Molecular Medicine, Laboratory of Experimental Oncology and Radiobiology, University of Amsterdam, Amsterdam, The Netherlands; 8 Institute of Biochemistry, Faculty of Biology and Chemistry, Justus-Liebig-University Giessen, 35392 Giessen, Germany; 9 Institute of Bioprocess Engineering and Pharmaceutical Technology, University of Applied Sciences Mittelhessen, 35390 Giessen, Germany; 10 Department of Mathematics, Natural Sciences and Computer Science, University of Applied Sciences Mittelhessen, 35390 Giessen, Germany; 11 Department of Nephrology and Medical Intensive Care, Charité-Universitätsmedizin Berlin, 10117 Berlin, Germany

**Keywords:** CLDN10, Renal cell carcinoma (RCC), DNA (hyper)methylation, Epigenetic editing, CRISPR-Cas9, Tumor suppressor

## Abstract

**Background:**

The kidney’s tubular system relies on cell polarity and tight junctions to maintain structure and function and disruptions contribute to diseases like cancer. Loss of tight junction proteins such as Claudins can actively contribute to tumorigenesis.

**Results:**

We aimed to identify biomarkers for renal carcinoma, after kidney transplantation and conventional kidney tumors. We identified the epigenetic silencing of the Claudin 10 gene isoform B (CLDN10B) through DNA hypermethylation in renal cancers, including clear cell (ccRCC), papillary (pRCC) and post-transplantation renal carcinoma (PT-ccRCC). In contrast, *CLDN10A* was hypomethylated in ccRCC and pRCC. Differential methylation of the isoforms discriminates RCC from other malignancies. The epigenetic alteration of *CLDN10B* significantly correlated with reduced patient survival and advanced tumor staging. CLDN10B overexpression or induction significantly inhibited migration, cell cycle progression, and cellular growth. Using a CRISPR-based epigenetic editing tool reactivated CLDN10B to its endogenous level using VP160 and TET1 by promoter demethylation and significantly demonstrated its tumor-suppressive effects in 2D and 3D cell models.

**Conclusion:**

Our findings suggest that CLDN10B acts as a tumor suppressor, and its epigenetic regulation may represent a therapeutic target for RCC. Ultimately, understanding CLDN10B’s regulation and function could provide new insights into renal cancer treatment.

**Supplementary Information:**

The online version contains supplementary material available at 10.1186/s13148-025-01911-2.

## Introduction

With an annual 430,000 documented cases and 18,000 estimated deaths worldwide (2020), renal cell carcinoma (RCC) is the most common malignant disease of the kidney [1]. Recently, incident cases increased by 30% [2, 3]. RCC typically originates from epithelial cells of the renal tubules and primarily metastasizes to the lungs, lymph nodes, bones, and liver [4]. The main histological subtypes of RCC are clear cell (ccRCC), papillary (pRCC), and chromophobe (chrRCC), with ccRCC being the most common, accounting for 75% of all cases [5]. Various pre-existing kidney conditions and an unhealthy lifestyle, such as smoking, promote the development of RCC. Renal tumors also arise in transplant recipients as post-transplantation malignancies (PTMs). PTMs represent a unique subset of cancers influenced by immunosuppressive therapies essential for preventing graft rejection. The incidence of PTMs is two to three times higher in transplant recipients compared to the general population, largely due to the compromised immune surveillance associated with prolonged immunosuppression [6]. PTMs also include clear cell renal cell carcinoma (PT-ccRCC) [7], which was the starting point of our study. In recent years, epigenetic modifications, particularly DNA hypermethylation, have been identified as key factors in tumor development. This process, mediated by DNA methyltransferases (DNMTs), adds a methyl group to the C-position of cytosine, forming 5-methylcytosine (5mC) in the CpG context. Specifically, DNA hypermethylation often occurs in CpG islands (CGIs), which are rich in CpGs. DNA methylation acts as a silencing signal and significantly influences transcription and gene expression by forming gene-silencing complexes. [8]. It is well established that many tumor suppressor genes are epigenetically silenced in cancer due to hypermethylation of the CpG islands. We have identified epigenetically silenced tumor suppressor genes in various tumor entities [9–15]. The development and metastasis of tumors include aberrant cell–cell communication and adhesion. Claudins (CLDN) are essential components of tight junctions (TJs), contributing to cell–cell adhesion and paracellular transport [16]. TJs prevent the uncontrolled exchange of ions and water, thereby playing a significant role in the polarity and survival of a cell. Dysregulation and loss of claudins have already been demonstrated in several cancers [17]. Renal tubules require a high degree of paracellular permeability and contain several claudins, including CLDN10 [18]. *CLDN10* is located on chromosome 13 (q32.1). It encodes three isoforms (A, antisense, B). The *CLDN10* gene harbors two alternative CGI carrying promoters that transcribe and translate into the two distinct CLDN10 proteins (CLDN10A and CLDN10B). Both isoforms have four exons, differing in their first exon. The significance of the *CLDN10* gene is highlighted by HELIX Syndrome (MIM# 617,671), a rare genetic disorder caused by biallelic loss-of-function variants in this gene (CLDN10B). The condition results in hypohidrosis (H), electrolyte imbalances (E), lacrimal gland dysfunction (L), ichthyosis (I) and xerostomia (X), associated with dehydration, muscle cramps, and hypotonia [18, 19]. Our study mostly focused on the CLDN10B isoform (230 kb, 25 kD), which regulates cations exchange [18]. Due to its role in cell polarity and epithelial integrity, the claudin family has garnered significant interest in cancer research [17]. Regarding carcinogenesis and epigenetics, *CLDN6* was found to be hypermethylated in breast cancer [20] and promoter hypermethylation silenced *CLDN7* in renal carcinomas [21]. *CLDN11* promoter hypermethylation is frequent in malignant melanoma of the skin [15] and nasopharyngeal carcinoma [22]. Regarding CLDN10 and cancer, studies reported it as an prognostic biomarker correlating with the immune microenvironment in ovarian cancer [23] and its expression with overall survival of lung adenocarcinoma patients [24]. In RCC, *CLDN10* is downregulated [25], and overexpression inhibited growth and metastasis [26]. CLDN10 expression was also reported to be negatively associated with the methylation levels of two CpG array probes, suggesting *CLDN10* to be an potential RCC biomarker [27]. A comprehensive epigenetic analysis of the CLDN10 gene has been lacking to date. In our study, we addressed this gap by investigating CLDN10 and showed that isoform B is epigenetically silenced in both post-transplant kidney tumors and conventional renal carcinomas. Our study also demonstrates the potential to reactivate CLDN10B's tumor-suppressive function through targeted epigenetic editing, suggesting it could be a promising therapeutic target for ccRCC and future clinical applications.

## Materials and methods

Methylation analysis, CoBRA and pyrosequencing were performed before [12]. The promoter region of *CLDN10* was analyzed by CpG plot http://www.ebi.ac.uk/Tools/seqstats/emboss_cpgplot/ and *UCSC genome browser*. Primers for bisulfite treated DNA were designed to bind only fully converted DNA and amplify the intended part of the promoter. The precise promoter region was chosen for CpG content and presence of restriction enzymes for CoBRA analysis. The size of the *CLDN10B* CoBRA PCR product is 172 bp (with *Taq1* site at 42 bp, 66 bp, 126 bp). For further details on CoBRA analysis see [28]. DNA was isolated after either proteinase K (Thermo Fisher Scientific) digestion of mechanically (Minilys) and extracted with phenol/chloroform and concentrations were determined. For CoBRA methylation analysis a total of 2 µg genomic DNA was bisulfite treated (5 mM hydroquinone, 1.65 M sodium metabisulfite and pH 5.5 with 0.025 M NaOH) and incubated overnight at 50 °C. DNA was purified using MSB Spin PCRapace (STRATEC Molecular), eluted in 50 µl H_2_O and followed by 10 min incubation with 5 µl 3 M NaOH at 37 °C. DNA was then precipitated with 70% ethanol and sodium acetate and resolved in H_2_O. Alternatively, 500 ng genomic/plasmid DNA and the EZ DNA methylation kit (Zymo research) were used according to the manufacturer's protocol. Bisulfite DNA was used for CoBRA PCR. The subsequent PCR product (CoBRA primers) was digested with 0.5 µl of *Taq1* (Thermo Fisher Scientific) 1 h at 37 °C and resolved on 2% TBE gel (0.5 x) together with a mock control and DNA ladder. Pyrosequencing was performed according to the manufacturer's protocol with PyroMark Q24 System (Qiagen).

Analysis of PTM patient renal samples using EPIC1 methylome. Patient DNA was obtained from University Hospital Leipzig (J. Halbritter) with ethics approval (#213–19-ek). Patient DNA was bisulfite-treated using EZ DNA methylation kit (Zymo research) according to manufacturer’s protocol. Methylome EPIC1 array (Illumina) analysis was performed at Life and Brain, Bonn. Methylome analysis was carried out using the R2 platform. Methylation status of *CLDN10B* was verified by CoBRA.

RNA expression analysis was performed before for RT-PCR [12]. RNA was isolated from human cell culture using Isol-RNA lysis procedure (Trizol, Thermo Fisher Scientific). RNA was *DNase*I (Thermo Fisher Scientific) treated and then reversely transcribed by MMLV (Promega). Quantitative RT–PCR was performed in triplicate/quadruplicate with SYBR select (Thermo Fisher Scientific) using Rotor-Gene 3000 (Qiagen) and normalized to *GAPDH/ACTB*. Normal human RNA from several tissues (pools) was obtained from Agilent. We performed RNA-Seq in cooperation with Prof. Bartkuhn incl. library preparation and RNA sequencing (RNA-Seq). For genome-wide analysis of gene expression, RNA sequencing libraries from isolated mRNA were generated and sequenced. A total amount of 1000 ng of RNA per sample was used to enrich for polyadenylated mRNA followed by cDNA sequencing library preparation utilizing the Illumina® Stranded mRNA Prep Kit (Illumina) according to the manufacturer’s instructions. After library quality control by capillary electrophoresis (4200 TapeStation, Agilent), cDNA libraries were sequenced on the Illumina NovaSeq 6000 platform generating 50 bp paired-end reads. The Illumina software bcl2fastq (v2.19.0.316) was utilized for demultiplexing and generating FASTQ files. Initial processing of the sequencing reads comprising quality control, filtering, trimming, alignment, and the creation of gene-specific count tables was conducted using the nf-core RNA-seq v3.7 bioinformatics pipeline (NEXTFLOW version 23.04.03) [29], with the homo sapiens GRCh38 genome and gene annotations downloaded from Ensembl (release 111). Data were uploaded to R2 for further analysis with type of analysis: ‘Metaanalysis’ by ‘K-means’ (10 × 10) with groups and 1500/20313 highest SD_2log genes used for heatmap generation, followed by ‘Find correlated genes with a single gene’ GO-term analysis of CLDN10 co-deregulated genes of dataset.

**Fig. 1:**
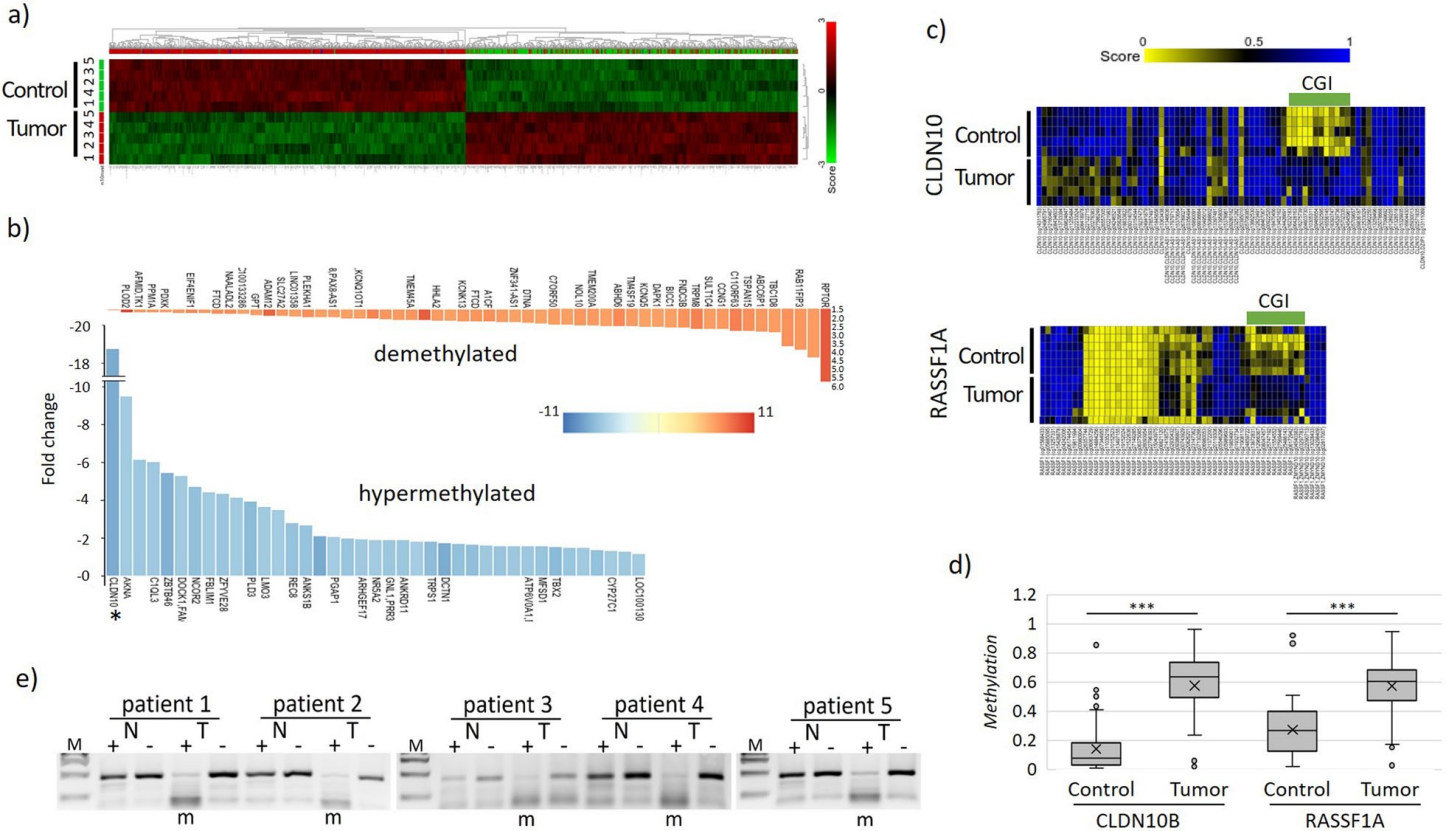
Identification *CLDN10* of differentially methylated candidates in PTM by methylation array EPIC1 of PT-ccRCC patient samples. **a** Differential methylation in PT-ccRCC samples (groups tumors and normal controls) depicted as heatmap (transform zscore, Anova 0.01 fdr). **b** Methylation changes as fold plot of n = 100 most significantly changed genes, *CLDN10* marked with asterisk. Blanks are not gene associated reporters. **c** Hypermethylation of CpG island in tumor samples for *CLDN10B* is shown. The candidate is depicted across its genomic region with hypermethylated CpG island in tumor vs. matching control. **d** Methylation changes across CGIs of *CLDN10B* and *RASSF1A *in tumor vs. control. *RASSF1A *serves as a positive control. **e** CoBRA methylation analysis for *CLDN10B* CpG island reveals specific hypermethylation of tumor samples vs. control in five patient samples. Digest with *Taq1* ( +) of PCR product shows specific tumor methylation vs. unmethylated normal samples. Additionally mock treatment (-) and 100 bp marker. CLDN10B specific PCR product is 172 bp

**Fig. 2:**
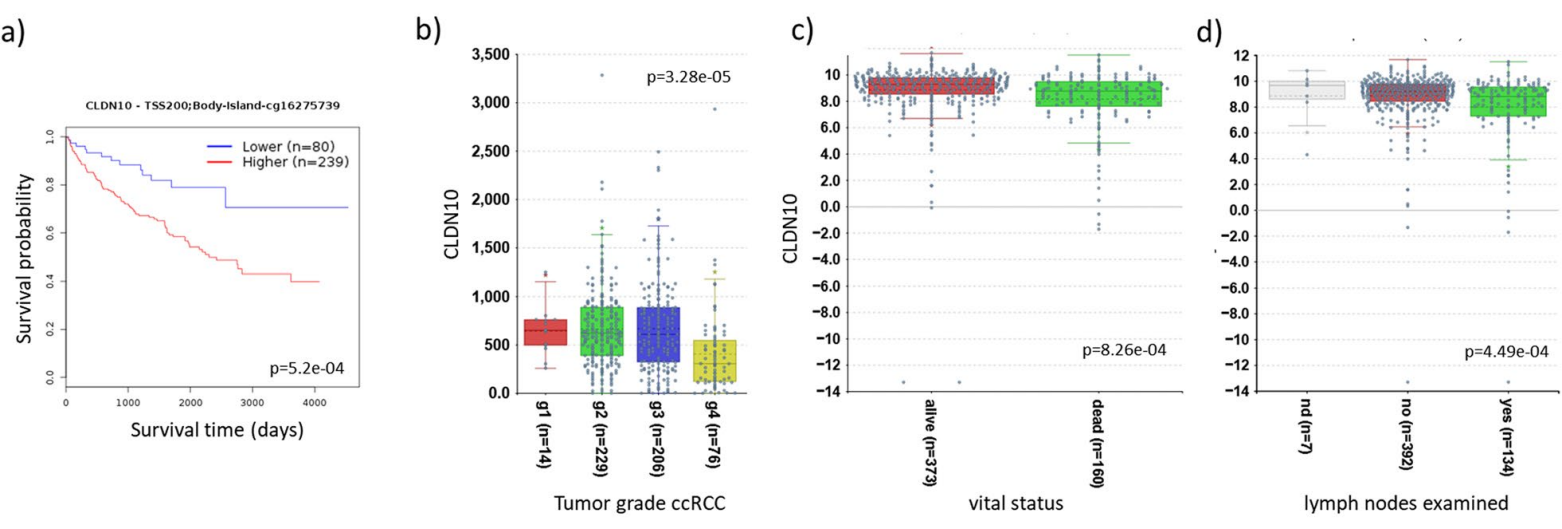
CLDN10 and clinical parameters of ccRCC.** a** Reduced survival probability with high methylation shown for *CLDN10B* (cg16275739 representative CpG 200 bp upstream of isoform B TSS, positioned in *CLDN10B* isoform CGI in TCGA ccRCC KIRC dataset, analyzed by KM MethSurv).** b** Decreased expression correlates with tumor grade for *CLDN10* across all stages (TCGA data ccRCC KIRC grouped by gene; separated by tumor grade; no distinction between isoforms possible, analyzed by R2) and correlation of clinical parameters** c** vital status relative to *CLDN10* expression and ** d** lymph nodes examined (no distinction between isoforms possible, analyzed by R2)

**Fig. 3:**
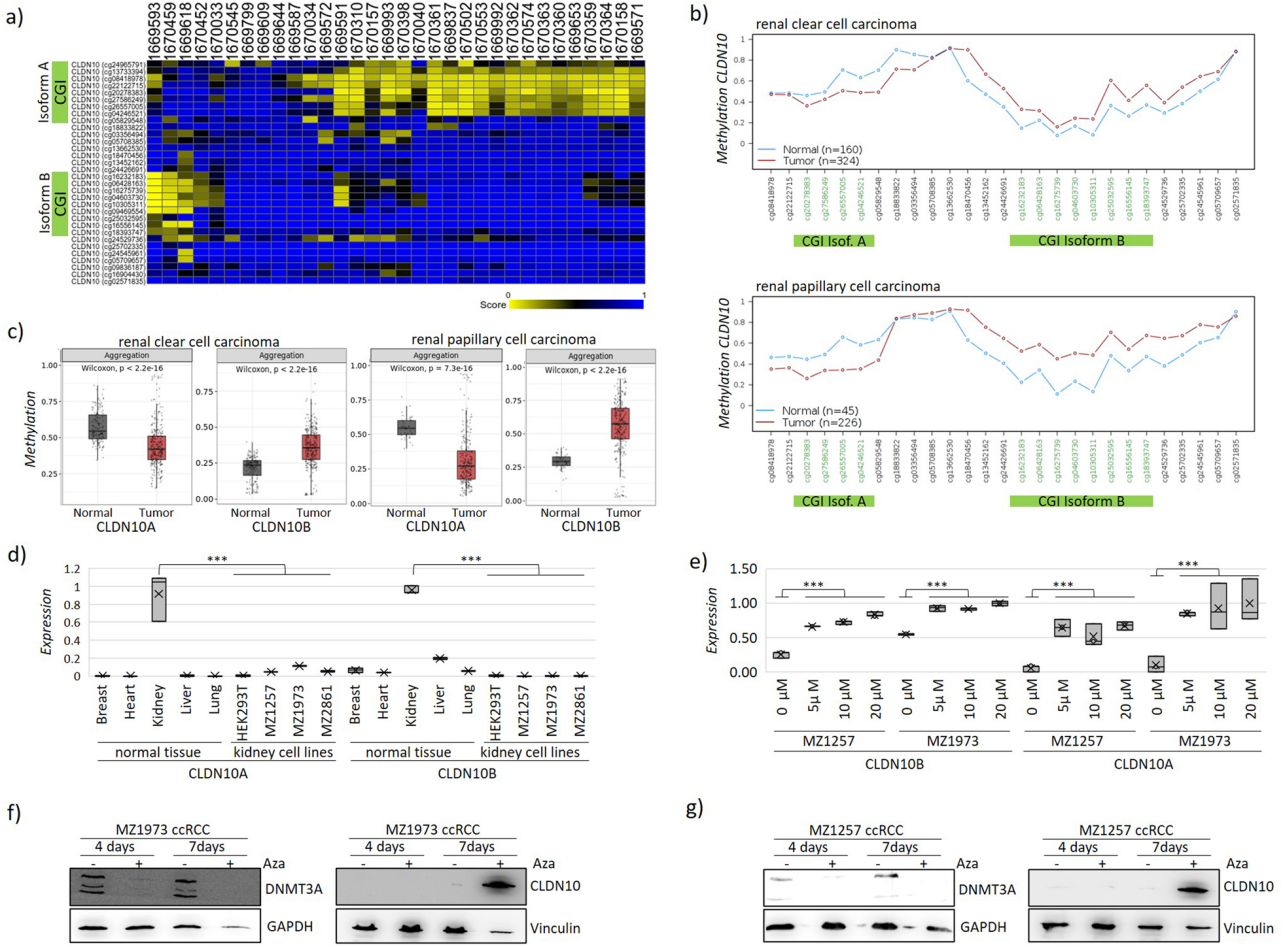
Differential methylation of *CLDN10* isoform A and isoform B in PTM ccRCC. **a** Methylome analysis (450 k array) of kidney cancer cell lines (Esteller dataset, gsm No, probes for *CLDN10*) depicts CGIs (green) for Isoform A with hypomethylation in yellow and isoform B with hypermethylation in blue (analysis R2). **b** Methylation profile across *CLDN10* promoters for both isoforms (ß-value) for kidney cancer (data TCGA Renal Clear Cell Carcinoma and Renal Papillary Cell Carcinoma, analysis Wanderer). **c** Significant demethylation of *CLDN10A* and significant hypermethylation of *CLDN10B* in ccRCC and in pRCC in red vs control in gray (data TCGA by Smart as ß-value, aggregated by mean). **d** Distinct expression pattern of *CLDN10* isoforms in normal tissues and loss of expression renal cell lines by qRT-PCR (normalized, normal tissues: heart, breast, liver, lung, kidney; cell lines: HEK293T transformed, cancer cell lines MZ1257/1973 are ccRCC). **e** Epigenetic reexpression of *CLDN10* isoforms upon pharmacological demethylation treatment by Aza (conc. 0 µM to 20 µM, signif. *p* < 0.001, t-test) in kidney cancer cell lines (ccRCC). **f** CLDN10B expression via Western Blotting in Aza treated ccRCC cell line MZ1973 (4d and 7d, 7,5 µM Aza) confirms CLDN10 (affinity AF0133) induction with DNMT3A (64B814 Thermo) reduction (Vinculin V9131 Sigma, GAPDH 14C10 cell signaling). **g** Equivalent result for MZ1257 are shown

**Fig. 4:**
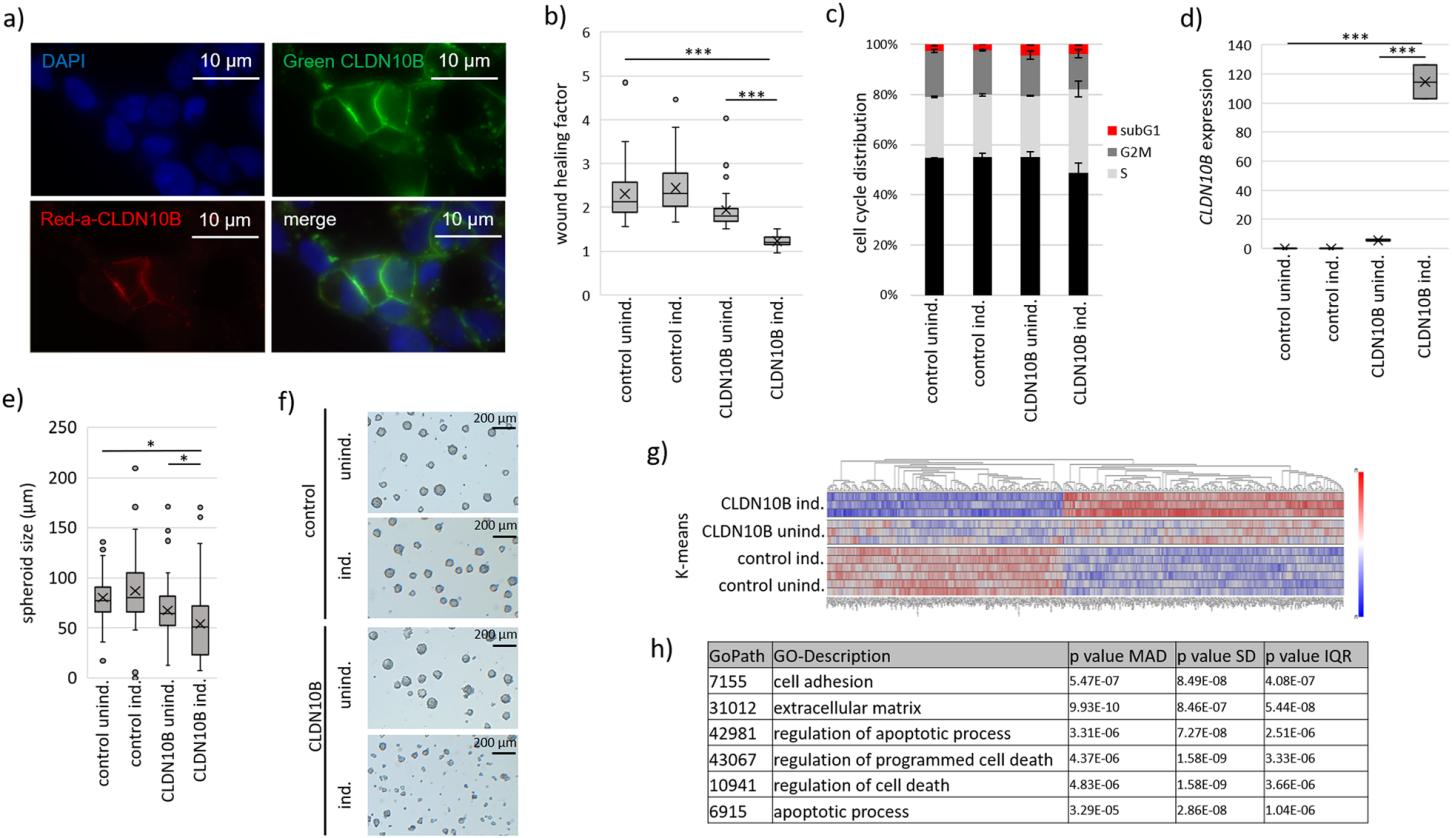
CLDN10 reexpression hinders cellular fitness 2D and 3D culture. **a** CLDN10 overexpression as EGFP fusion in fluorescence microscopy in HEK cells (24 h) and CLDN10 induction in HEK Trex inducible cell line (24 h), together with CLDN10 counterstaining (red). **b** Induction of CLDN10 in 2D culture after reveals diminished wound healing capacity. **c** Cell cycle arrest upon CLDN10 induction after 24 h in HEK Trex measured by flow cytometry (propidium iodide stained) using BDCantoII. **d-f** 3D cell line growth was established for CLDN10 inducible cell line HEK Trex and control cell line under and dynamic cell culture conditions. Reduced spheroid size after 48 h upon CLDN10 induction (doxycycline) in CLDN10-GFP inducible cell line HEK Trex (vs. GFP control cells),** f**) microscopic growth reduction (48 h) and **d** according CLDN10 expression induction (RNA level, 72 h). **g-h** Transcriptome analysis of 3D cell model upon CLDN10 induction reveals an association with GO term cell death. Cells were seeded for 3D formation in dynamic cell culture and dox induced. Spheroids were harvested after 3 days for RNA isolation, reverse transcription and RNA-seq. **g** K-means clustering (3 groups, log2 zscore, passes/rounds set 10) shows separation of CLDN10 from control cell line with heatmap of top-deregulated genes.** h**) Upon CLDN10 induction co-deregulated genes is significantly correlated with several GO-terms

**Fig. 5:**
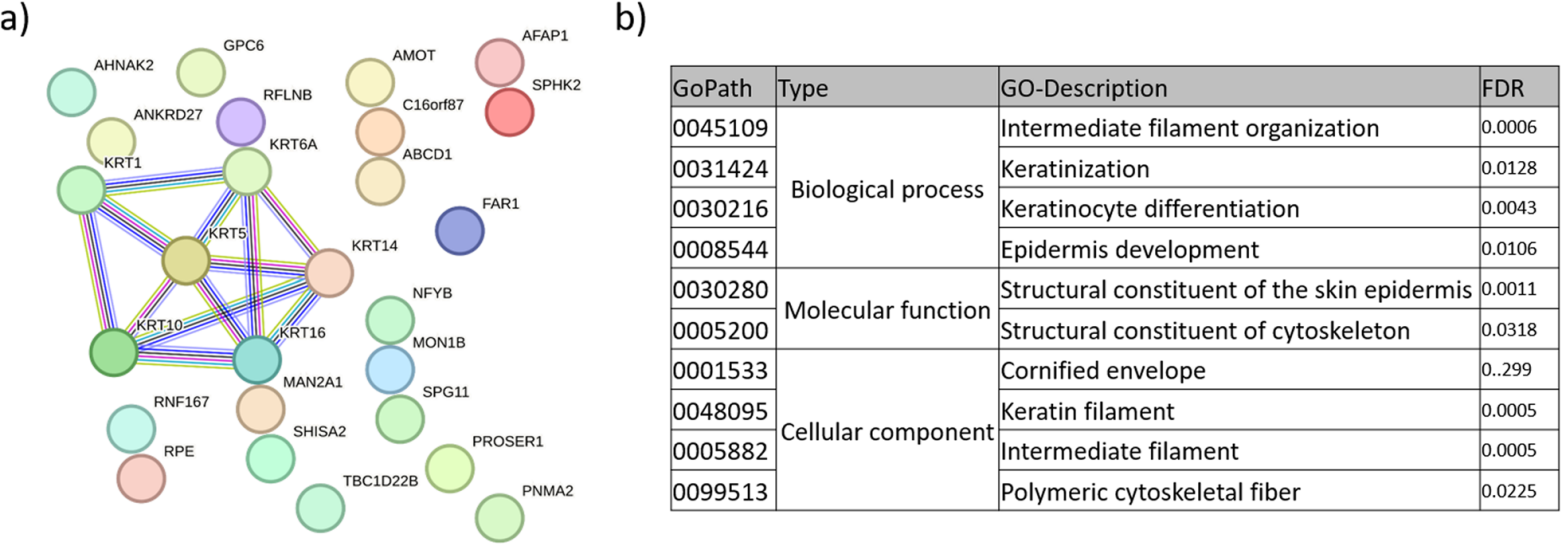
CLDN10B signaling pathway induction. Whole proteome analysis upon CLDN10B induction reveals reexpression association with GO-term keratinization and intermediate filaments. HEK293T cells were transfected with Cas9 under guidance with *CLDN10B* guide #2, cultured for three days and harvested. Experiment was performed in quadruplicates. **a** Significant network appears upon CLDN10B induction with data being analyzed by string, filtering by effect > 0.5, with number of nodes n = 26 and protein–protein interaction enrichment p-value 4.57e-09 and **b** associated GO term of biological process, molecular function and cellular component. Likewise filtered proteome by effect < -0.5 gave no significant results (String)

Methylation, expression, network analysis of publicly accessed data and origin of data (see [12]). Gene expression, promoter methylation correlation and Kaplan–Meier calculations were performed using R2 Genomics Analysis and Visualization Platform [30], Wanderer [31], KM Plotter [32] and MethSurv [33], Human Protein Atlas [34], cbioportal [35], binding partner Network depiction/analysis by String v11 [36] and IntAct database search [37]. The following datasets are listed in order of appearance with resource of data: Fig. 1: own data generated from patient material from PTM-ccRCC with matching control tissues Mixed Renal cell carcinoma (PTM)—Richter—custom—ilmnhmepic; Fig. 2: TCGA ccRCC KIRC dataset; Suppl. Table 2: Top biomarker analysis of ccRCC in KIRC was performed by Methsurv. Figure 3: only kidney cell lines from Cellline Cancer Pharmacogenomic—Esteller—1028—custom—ilmnhm450, Source: GEO ID gse68379 Dataset Date 2016–07-05; TCGA Renal Clear Cell Carcinoma and Renal Papillary Cell Carcinoma; Suppl. Figure 6: Normal Tissues—Lokk—70—custom—ilmnhm450, Source: GEO ID: gse50192 Dataset Date: 2014–02-26; Normal Tissues—Slieker—56—custom—ilmnhm450, Source: GEO ID: GSE48472 Date: 2013–08-06, Mixed Renal cell carcinoma (PTM)—Richter—custom—ilmnhmepic; Suppl. Figure 7: Cellline Cancer Pharmacogenomic—Esteller—1028—custom—ilmnhm450, Source: GEO ID: gse68379 Dataset Date: 2016–07-05; Suppl. Figure 8: Tumor Kidney Chromophobe—TCGA—66—rsem—tcgars, Source TCGA ID KICH Date 2000–01-01; Suppl. Figure 7: kidney sections Samples CAB012969 Female age 41, patient 2530, HPA042348 Male age 61, patient 1859, Suppl. Figure 8 cbioportal compilation of RCC data sets of 1814 samples from 1744 patients in seven studies; Fig. 4 Exp—HEK-TRex—Richter—12—DESeq2_rlog—gse140768, Fig. 5 Exp—Cell line (guided vs non-guided)—Richter—8—diann—prot230928.

Cell culture. 2D cell culture: (see [12] for details). Cell lines were grown in appropriate medium supplemented with 10% FCS and 1% Penicillin/Streptomycin under cell culture conditions (37 °C, 5% CO_2_). Cell lines were transfected using Turbofect (Thermo Fisher Scientific), X-tremeGENE HP (Roche) or Polyethylenimine (in house produced, branched PEI) with either 4 µg (6 well) or 10 µg (10 cm dishes), in combination with serum-reduced medium Opti-MEM (Gibco: Thermo Fisher Scientific, Dreieich, Germany), according to the manufacturer’s protocol. For 5-Aza-2’deoxycytidine (Aza) treatment cancer cell lines were split to 10% density, Aza was added with fresh medium on four consecutive days at 5 µM, 10 µM and 20 µM and RNA as well as DNA were isolated. For CLDN10 expression confirmation under Aza treatment, we treated ccRCC cell line MZ1973 for 4 and 7 days with 7,5 µM Aza and isolated protein. For western blotting see [38]. Antibodies used are: CLDN10 (affinity AF0133), DNMT3A (64B814 Thermo), Vinculin (V9131 Sigma) and GAPDH (14C10 cell signaling). 3D cell culture: Static cell culture was conducted in U-shaped, cell-repellent plates with a total volume of 200 µl and initial centrifugation at 300 g to facilitate cell settling. Volume based growth rate was calculated [39]. For dynamic 3D cell culture, 1.5 million inducible HEK293 TREx cells were transferred into flasks. After 6 h, cells were induced with 2 mg/ml of doxycycline and analyzed by microscopy 72 h later. For dynamic epigenetic editing 3D cell culture, cells were firstly transfected with VP160-dCas9 in 2D cell culture. VP160-dCas9 transfected cells were Puromycin (2.5 µg/ml) selected in 2D, then transferred into flasks (72 h post transfection). Flasks were shaken for 2 days and spheroids analyzed by microscopy (Suppl. Figure 20b). The kidney cell lines HEK293T (HEK, RRID: CVCL_0063) and HEK293-TREx (CVCL_D585) were used for functional assays, therefore were short tandem repeat (STR) confirmed within the last 3 years (Eurofins Genomics, Ebersberg, Germany). All cell lines, including the kidney cancer cell lines, were mycoplasma-free. Stable CLDN10B-inducible HEK cell line (see for earlier use [40]: HEK293 cells, which stably express the Tet Repressor under Blasticidin selection (5 µg/ml, Roth), were obtained from Thermo Fisher Scientific as part of the TetOn-TREx system and served as the control cell line. These cells were transfected with the cloned CLDN10B-GFP-pcDNA4ToMyc (from CLDN10B-GFP plasmid [18]) for stable integration of Doxycycline-inducible CLDN10B. Following transfection, cells were selected with Zeocin (500 µg/ml, Thermo Fisher Scientific). Similarly, stable cells expressing only EGFP were generated as controls. For final CLDN10B induction Doxycycline (2 mg/ml) was used. CRISPR-based induction of CLDN10B: Firstly, we identified relevant regulatory elements within the *CLDN10B* promoter such as E-boxes. In overlap with the E-box guide #2 for *CLDN10B* was positioned. Secondly, HEK293T cells were transfected with Cas9 (nuclease active, wt) together with *CLDN10B* guide #2. Cells were cultured for three days, and then harvested for RNA and expression analysis as well as protein lysate for mass spectrometry. The experiment was conducted in quadruplicates.

Mass spectrometry. For further details, please refer to the Supplementary Methods. Cells used for whole proteome analysis following CLDN10 induction (performed in quadruplicates) were lysed, and 50 μg of protein was extracted for subsequent processing. Similarly, for the interactomics experiment, proteins were eluted from beads, lysed, and prepared for further processing. All samples were reduced and alkylated. A modified version of the SP3 method [41] was used for further sample preparation. Protein digestion was performed with trypsin. Peptides were purified using solid phase extraction on C18 microspin columns according to the manufacturer's instructions (Macherey–Nagel), based on the original protocol [42]. Peptides for the whole proteome analysis upon CLDN10 induction were analyzed by liquid chromatography–mass spectrometry (LC–MS/MS) carried out on a Exploris 480 instrument connected to an Ultimate 3000 rapid separation liquid chromatography instrument data-independent-acquisition method. Purified peptides from the CLDN10 interactomics were analyzed by LC–MS/MS carried out on a Bruker Daltonics timsTOF Ultra instrument connected to a Bruker Daltonics nanoElute instrument with a 45 min gradient data-independent-acquisition method. Peptide spectrum matching and label-free quantitation were subsequently performed using DIA-NN [43] and a library-free search against the Human Uniprot.org database (20,407 reviewed Swiss-Prot entries; April 2023). Downstream data processing and statistical analysis were carried out by the Autonomics package developed in-house (version 1.13.19). Proteins with a q-value of < 0.01 were included for further analysis. The full list of settings, code for data processing and statistical analysis are uploaded along with the mass spectrometric raw data to the ProteomeXchange Consortium with dataset identifier: PXD056422, via the MassIVE partner repository (https://massive.ucsd.edu/, MassIVE-ID: MSV000095998; 10.25345/C5NK36H32).

Cell cycle analysis, immunofluorescence and wound healing assay (for further details see [11, 12, 44].**).** Regarding flow cytometry analysis cells were transfected and ethanol fixed at indicated time points. The following day cells were treated with 50 µg/ml *RNase*A for 30 min at 37 °C. Subsequently, cells were stained with 50 µg/ml propidium iodide prior to measuring DNA content in FACSCantoII (BD Biosciences). FACSDiva Software (BD Biosciences) was used for measurement/gating to distinguish transfected fluorescent cells and to determine cells in G0/G1, S and G2/M phase of the cell cycle. For localization analysis cells were seeded on glass slides and transfected the following day. Cells were fixed with 3.7% formaldehyde at according time points, permeabilized using tritonX, stained with DAPI (0.1 µg/ml in PBS, Sigma), embedded in anti-fading with Mowiol (Sigma) and analyzed with Axio Observer Z1 (Zeiss) under 63 × magnification and Volocity Software (Perkin Elmer). For wound healing assay, cells were transfected or induced as described and grown to confluence before a scratch was placed with pipette tip. Wound closure was measured at day 0 and 24 h / 48 h later and is depicted in % gap closure, assuming that time 0 corresponds to a wound width of 100%. A Transwell migration assay (Corning, Wiesbaden, Germany; 24-well format) was conducted using the MZ1973 ccRCC cell line, transfected with X-tremeGENE HP (Roche, Mannheim, Germany) for CLDN10B-EYFP expression and control cells with EGFP alone. Both cell populations were co-transfected with a puromycin resistance plasmid for positive selection with Puromycin (2.5 µg/ml). After selection, cells were counted, and equal numbers (20.000 cells) were plated into the Transwell inserts. The lower chamber contained DMEM supplemented with 10% FBS, while the upper chamber had 5% FBS to promote migratory attraction. After the designated time point, the inserts were removed, the inside of the inserts was cleaned with a cotton swab to eliminate non-migrated cells, and both the inserts with outer migrated cells were fixed with formaldehyde and stained with toluidine blue. Cell numbers were then counted microscopically.

Epigenetic editing by CRISPR-dCas9. The CRISPR-Cas technique in mammalian cells has now been developed and widely studied in genetics. We are using the nuclease deficient dCas9 with point mutations of its two nuclease domains HNH/RuvC, rendering it unable to cleave DNA but retaining the ability to target genomic regions [45, 46]. We are using fusion proteins of the dCas9 enzyme to effector domains/gene-regulatory proteins, which enables stable and efficient transcriptional activation, with the site of delivery determined solely by a co-expressed short guide (sg)RNA [47]. We used the VP160 transcription activator, originally from herpes virus protein VP16, and TET1, the ten-eleven translocation methylcytosine dioxygenase 1 and its catalytic domain is used for CRISPR approaches [48]. A 6-well dish of cells with transfected EYFP served as a transfection control. The medium was changed 5 h after transfection. Transfection efficiency was checked after 24 h by fluorescence microscopy. Puromycin (Gibco: Thermo Fisher Scientific, Dreieich, Germany) selection was performed at 2.5 µg/ml from 24 h after transfection. RNA, DNA were isolated 120 h after transfection. *CLDN10B* sgRNAs/Oligos were positioned/generated using Benchling and cloned into modified px459 delSpCas9(BB)- 2A-Puro V2.0 (modified Addgene #62,988, delCas9, available on request) [12]. *CLDN10B* sgRNAs are #1, #2, #3, #4, #5, #6 (Suppl. Table 1.) and are positioned relative to TSS at -259 #1, -179 #2,—19 #3, + 121 #4, + 247 #5 and + 345 #6. A combination of all sgRNAs was used for all experiments. As the non-guided control, we used a sgRNA without a human target sequence alignment CAAAATTGCAAGCGTCAAAG. We calculated their off-targets and found no off-targets for up to one mismatch, the CLDN10 guides specifically detect CLDN10B. One off-target is calculated found for #2 and #3 with two mismatches, but not within a relevant regulatory promoter region (Suppl. Table. 6).

Availability of data and materials. The datasets supporting the conclusions of this article are available in the MassIVE partner repository, dataset identifier: PXD056422, via the https://massive.ucsd.edu/, MassIVE-ID: MSV000095998; 10.25345/C5NK36H32: and in the Genomics Analysis and Visualization Platform, dataset identifier: Mixed Renal cell carcinoma (PTM)—Richter—12—custom – ilmnhmepic, Exp HEK-TRex—Richter—12—DESeq2_rlog—gse140768 https://hgserver1.amc.nl/cgi-bin/r2/main.cgi. Transcriptomic data are found in the NCBI's Gene expression omnibus under the accession GSE283785 (https://www.ncbi.nlm.nih.gov/geo/query/acc.cgi?acc=GSE283785).

**Fig. 6:**
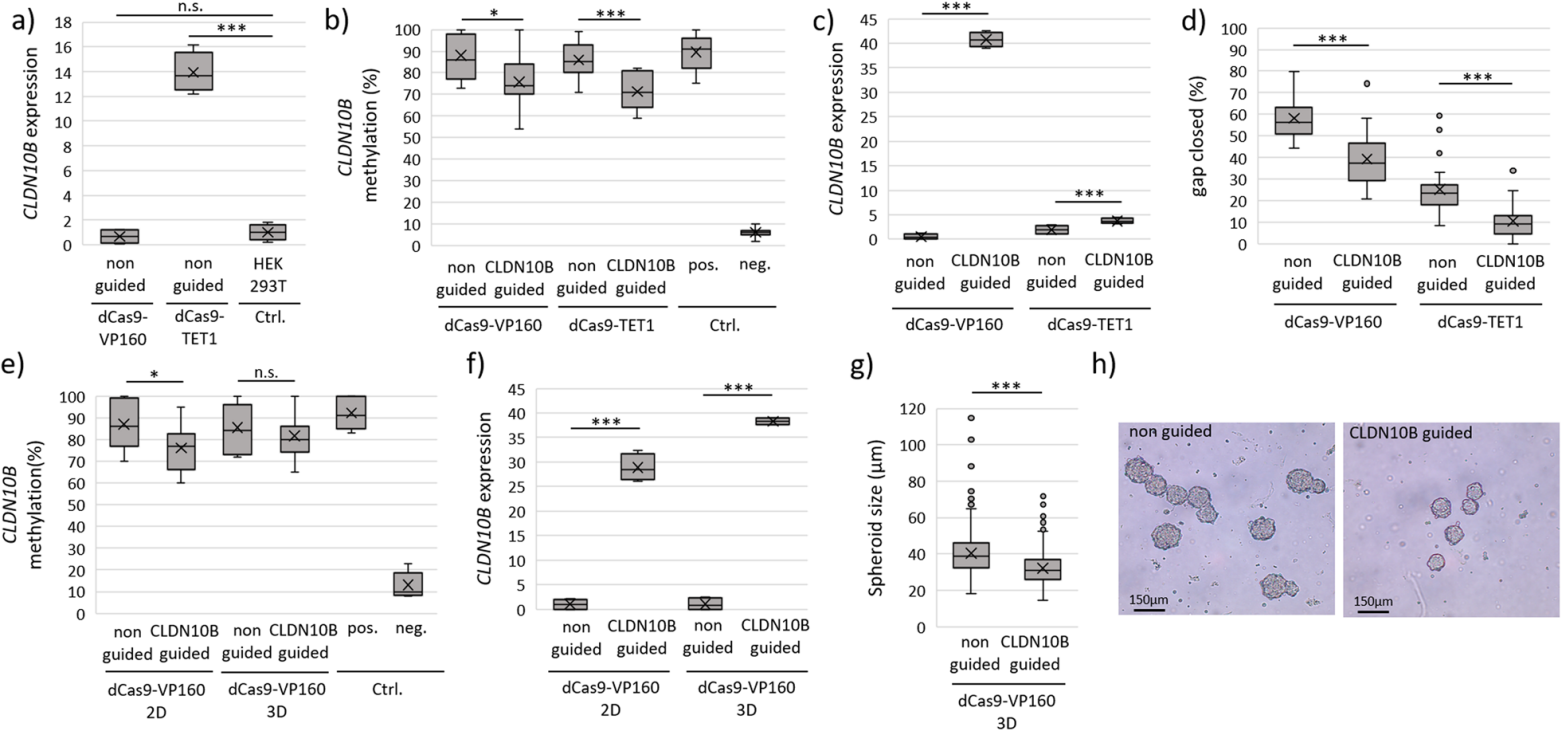
Epigenetic editing with gene reexpression, promoter demethylation and reactivation of tumor-suppressive function is achieved by VP160 and TET1 effectors on CLDN10B. For epigenetic editing of *CLDN10B,* HEK cells were transfected with according CRISPR-dCas vectors (either VP160 or TET1), RNA isolated, reversely transcribed and expression analyzed by RT-PCR. DNA was isolated, BS treated and *CLDN10B* CGI pyrosequenced. **a** dCas9-TET1 increases the endogenous background level (non-guided) of *CLDN10B* expression, compared to untreated HEK cells. **b** *CLDN10B* was demethylated by VP160 and TET1, that reactivated CLDN10B expression (**c**) and its tumor-suppressive function (d). **d** Wound healing assay in 2D cell culture reveals reduced cell migration after EpiEdit treatment. Scratch was placed 72 h post transfection, wound closure was examined by measuring the cell-free area at 0 h and 48 h (120 h post transfection) (Suppl. Figure 18 a)). **e–g** d Cas9-VP160 treatment of HEK cells in 2D and dynamic 3D cell culture. 3 Mio. cells were transferred 72 h post transfection (2D) into dynamic 3D cell culture (analyzed 120 h post transfection) (Suppl. Figure 18 b)). Demethylation of *CLDN10B* by dCas9-VP160 (e) leads to reexpression **f** and consequently to a reduced microscopic spheroid size (g + h)

Statistical analysis was performed according to experimental setup. Significance abbreviations: * < 0.05, ** < 0.01, *** < 0.001, n.s. not significant. Analysis performed by indicated software in Figure text and Fig. 1a Anova 0.01 FDR, Fig. 1d t test by excel/graphpad, Fig. 2a likelihood ratio (LR), Fig. 2b-d Anova, Suppl. Figure 4 Anova, Fig. 3c Wilcoxon, Fig. 3d + e t-test, Fig. 4 b/d/e t-test, Suppl. Figure 12 g Anova, Suppl. Figure 14a t-test and Fig. 6 t-test.

CLDN10 isoform analysis: For the analysis of the isoform composition and alternative splicing, UCSC annotated reference sequence mRNAs NM_182848 (CLDN10A1), NM_001160100 (CLDN10A2) and NM_006984 (*CLDN10B*) from genome version GRCh38/hg38 were used. For visualization of publicly available RNA-Seq and gene structure data, we utilized the UCSC Genome Browser (www.genome.ucsc.edu). Protein domain predictions were done using SMART (www.smart.embl-heidelberg.de/), predictions of extracellular versus intracellular domains was derived from dbPTM (www.awi.cuhk.edu.cn/dbPTM/).

Plasmids and Primers. Please see Supplementary Table 1.

## Results

To date, post-transplantation malignancies (PTM) of RCC are not intensively studied, especially information of its epigenome is lacking. To identify novel biomarkers, we took a whole methylome approach studying the alteration of the epigenome (DNA-methylation) of renal PTMs. Paired primary patient material from five clear cell RCC (PT-ccRCC) together with their normal adjacent control tissue was used for DNA isolation and subsequent bisulfite conversion before methylation array (EPIC). Using these methylome data, we identified differentially methylated regions and genes in the group of post-translation clear cell renal cell carcinoma against their corresponding matching patient control tissue. PT-ccRCC and normal tissue showed differential methylation as depicted in heatmap (transformed z score, 1312 probes met criteria, Anova p value < 0.01, multiple testing correction FDR). To choose the most biologically relevant genes, we sorted the possible differentially methylated candidate genes by the number of EPIC probes that corresponded to a gene, and calculated with at least 50% of reporters per gene to be differentially methylated, calculated the fold change per reporter and sorted by their differential methylation in relation to CGI. We found the *CLDN10* gene to be the top candidate (Fig. 1a,b) and genomically is characterized by two distinct CGIs (Suppl. Figure 1). A clear distinction between PT-ccRCC and the corresponding control tissue was observed (Fig. 1a).

The highest number of differentially methylated single CpG probes corresponded to *CLDN10B* (Fig. 1b, fold plot 100 most significant genes). The *CLDN10* gene is transcribed as isoform A from the CGI with low CpG content (Suppl. Figure 2a), and as isoform B from a major CGI with high CpG content (Suppl. Figure 2b), and an additional antisense transcript (Suppl. Figure 1,4). Alternative promoter usage results in an alternative exon 1 in isoform A and B. Isoform A can be subdivided into two alternative splice variants, named A1 and A2 here (Suppl. Figure 4a). An alternative 5’-splice site in exon 1 adds 19 amino acids to the first extracellular domain of protein isoform A1 compared to A2 (Suppl. Figure 4b, c). This has been previously reported [49]. In isoform B, the complete first transmembrane and extracellular domain are different (Suppl. Figure 4c). Interestingly, intron 1 of all isoforms contains a highly conserved, U-rich element of unknown function (Suppl. Figure 4d).

Across human tissues, kidney is the predominant expression site of *CLDN10* (Suppl. Figure 3, GTex). The isoform A is expressed mostly in the kidney, but also in skin, pancreas, salivary gland, and to a lesser extent in the brain (Suppl. Figure 3). The B isoform is also expressed in the kidney but also in uterus, testis, skin, pancreas, salivary gland, lung, liver, fallopian tube, esophagus, cervix, brain and spinal cord (Suppl. Figure 3). Overall, *CLDN10B* expression dominates over the level of *CLDN10A* expression. Regarding its promoter methylation level, pattern of *CLDN10* differential methylation was observed across the CGI of the *CLDN10* isoform B, which is hypermethylated in comparison with the controls (Fig. 1c, Suppl. Figure 1). Hypermethylation was also observed for the bona fide tumor suppressor *RASSF1A* (Fig. 1c), well known to be hypermethylated and inactivated in ccRCC [50, 51], serving as a positive control for our PT-ccRCC set. We observed a significant methylation increase of the CGIs of *CLDN10B* and *RASSF1A* in PTM vs. control. *CLDN10* methylation of tumors was increased by more than 40% from below 20% of methylation in the control samples and similarly, also for *RASSF1A* methylation increased by 30% in PTM vs. control (Fig. 1d). We verified *CLDN10B* methylation in PT-ccRCC using another methylation analysis method (CoBRA, Suppl. Figure 2c) and found *CLDN10B* hypermethylation only in the patient tumor samples, but not the patient matching normal controls (Fig. 1e). Additionally, we found that clinically relevant parameters correlated with *CLDN10B* in conventional ccRCC from TCGA data. Its hypermethylation in ccRCC significantly correlated with reduced patient survival (Fig. 2a). Regarding its expression levels, *CLDN10* expression was significantly decreased with advanced tumor stage, but already present from stage g2 in ccRCC (Fig 2b). Consistently, loss of *CLDN10* expression significantly correlated with decreased patient survival of ccRCC [26]. Interestingly, tumor grade for ccRCC, but also pRCC correlated with increased methylation of the *CLDN10B* gene and was significant for cg10305311, that is positioned 100 bp upstream of the TSS (Suppl. Figure 5). These results indicate that *CLDN10* methylation occurs early in carcinogenesis, rather than at later stages. This hypermethylation aligns with existing literature, which suggests that the inactivation of tumor suppressors, such as *RASSF1A*, often occurs early during carcinogenesis, rather than as a late event, in several cancers such as renal tumors [52]. Additionally, *CLDN10* expression correlated with further clinical parameters such as vital status and lymph nodes examined (Fig. 2c,d).

Regarding *CLDN10* methylation pattern in RCC, we observed the differential methylation of *CLDN10* that appeared to be isoform specific with *CLDN10B* being strongly hypermethylated, whereas *CLDN10A* was slightly hypomethylated in PT-ccRCC (Fig. 1c).

The differential *CLDN10* methylation was also observed in ccRCC cell lines, with a strong hypermethylation of the B isoform and associated hypomethylation of the A isoform (Fig. 3a). The results were verified in primary tumors from renal clear cell carcinoma and renal papillary carcinoma of TCGA data in comparison to controls that showed the same pattern (Fig. 3b). The aggregated methylation across the CGI of *CLDN10A* was significantly reduced and of *CLDN10B* significantly hypermethylated in tumor to normal samples from TCGA renal clear cell carcinoma (Fig. 3c). In renal papillary cell carcinoma, similarly, the aggregated methylation across the CGI of *CLDN10A* was significantly reduced and of *CLDN10B* significantly hypermethylated (Fig. 3c). In normal control tissues however, the promoter of *CLDN10A* is methylated, whereas the B isoform is unmethylated (Suppl. Figure 6a-b), which is also the case in renal control normal tissues (own dataset PTM patients, Suppl. Figure 6c). We concluded that during carcinogenesis the *CLDN10B* promoter is turned into a hypermethylated state, whereas the A isoform is demethylated. Ultimately, with this *CLDN10* differential methylation pattern, identification of kidney cancer (ccRCC) cell lines from a large dataset of cancer cell lines is possible (Suppl. Figure 7). It must be mentioned that in chromophobe renal cell carcinoma we found only *CLDN10A* hypomethylation, but not *CLDN10B* hypermethylation (Suppl. Figure 8). In summary, *CLDN10A* hypomethylation and *CLDN10B* hypermethylation are specific for ccRCCs and pRCCs. Interestingly, an analysis of the top 200 CpG biomarkers from the KIRC dataset, representing ccRCC, revealed that neither CLDN10 nor RASSF1A were among them (Suppl. Table 2). This highlights the importance of our follow-up study comparing PT-ccRCC with conventional ccRCCs. Regarding expression, both isoforms are expressed in healthy human kidneys, but only *CLDN10B* is expressed in other tissues (Fig. 3d), confirming the Gtex expression data (Suppl. Figure 3). On the protein level expression for CLDN10 was assessed (The Human Protein Atlas) and it was detectable in the tubulus system (Suppl. Figure 9). Interestingly, in the highly proliferative kidney cell lines the expression of *CLDN10B* is lost (transformed HEK293T and ccRCCs), but *CLDN10A* is detectable (Fig. 3d). To test the epigenetic responsiveness to DNA methylation of *CLDN10*, we treated ccRCC cell lines with a pharmacological inhibitor of DNMTs (5-Aza-2’-deoxycytidine, Aza), that hinders methylation of DNA across the epigenome. We found that *CLDN10A* and *CLDN10B* expression were significantly restored upon Aza treatment, with *CLDN10B* being significantly more responsive (Fig. 3e). Protein levels of CLDN10 were restored under Aza treatment in ccRCC cell line MZ1973 and MZ1257, and Aza driven DNMT inhibition was confirmed by reduction in DNMT3A protein levels (Fig. 3f + g). Mutation frequency of the CLDN10 open reading frame, however, is rare in RCC (Suppl. Figure 10). Overall, our data conclusively show the epigenetic regulation of *CLDN10B* by DNA hypermethylation in ccRCC. The methylation data of *CLDN10* in renal cancer (ccRCC and pRCC) and PT-ccRCC led to our hypothesis of CLDN10B as a potential tumor suppressor in different types of renal cancer.

Our data highlighted the major decrease in *CLDN10B* expression in renal carcinomas caused by promoter hypermethylation; we therefore generated a stable HEK293 TREx CLDN10B-GFP cell line. We used the immortal embryonic kidney cells with an inducible tetracycline/doxycycline system to reexpress CLND10 and test its tumor-suppressive function. To validate the GFP fusion in the cell line and for localization of CLDN10B, we induced the cells with doxycycline and fixed them on glass slides 24 h post-transfection. CLDN10B-GFP localized in/near the cell membrane using fluorescence microscopy (Fig. 4a). For verification, we counterstained CLDN10B by immunofluorescence (Alexa Fluor 405, red staining, Fig. 4a). As a property of a potential tumor suppressor gene, we investigated the proliferation and migration behavior of the HEK293 TREx CLDN10B cells (Fig. 4b). We performed a wound healing experiment, induced the stable cells and compared them with uninduced cells. As a negative control, we used HEK293 TREx cells without containing CLDN10B (control cell line). After 24 h hours of CLDN10B induction, we observed a 37% reduction in cell migration, while the negative control showed no reduction (Fig. 4b and Suppl. Figure 11a). To verify that CLDN10B negatively regulates migration, we conducted transwell assays in the ccRCC cell line MZ1973 and observed a reduction in migratory potential upon its overexpression (Suppl. Figure 11b). In addition, we performed cell cycle analysis in HEK293 TREx CLDN10B by flow cytometry, where the nuclei were stained with propidium iodide. Compared to the negative control and the uninduced cells, the CLDN10B-induced cells showed cell cycle arrest in the form of an S-phase accumulation (Fig. 4c). To further investigate the behavior of the cells under CLDN10B induction, we established the used TREx cell line in a dynamic 3D cell culture system. We achieved a 21-fold reexpression of *CLDN10B* (Fig. 4d), which resulted in a significant 20% reduction of spheroid size (Fig. 4 e–f, Suppl. Figure 12). Interestingly, initial CLDN10B aggregation was observed after 24 h, which is expected as the expression of CLDN10B tight junctions promotes cellular aggregation (Suppl. Figure 12, 24 h), preceding any noticeable growth suppression. A reduction in growth over time due to CLDN10B induction was observed not only under dynamic cell 3D cell culture conditions, but also under static cell culture conditions. CLDN10B growth reduction effects were characterized by decreased 3D spheroid diameter, slower growth rate, and was microscopically visualized (Suppl. Figure 13). The spheres from the 3D dynamic CLDN10B-induced culture were harvested and used for transcriptome analysis using RNA-seq. CLDN10B induction altered the RNA expression profile and *CLDN10* levels significantly associated with GO terms cell adhesion, extracellular matrix and cell death (Fig. 4 g-h, Suppl. Figure 14). For verification, we additionally applied a transient transfection method with a CRISPR-based (Cas9) induction of CLDN10B by targeting the double E-Box (CAGCTG/CAGATG) motif within the *CLDN10B* promoter at position -179 of the TSS (by gRNA #CTGCAGATGGAGAACCCGGG) (Suppl. Figure 15). *CLDN10B* expression was strongly and significantly induced (Suppl. Figure 16a) by this approach. Samples subjected to whole proteome analysis by mass spectrometry led to a clear separation into groups guided vs. non-guided (Suppl. Figure 16b).

This CLDN10B induction was associated with alterations to the proteome with GO terms intermediate filament organization, keratinization, epidermis development (Fig. 5 a-b). In a detailed view, Keratin (KRT) proteins enrichment upon CLDN10B induction was visualized in proteome and as direct comparison between groups (Suppl. Figure 17a-b). In summary, we observed that CLDN10B induction led to distinct transcriptomic and proteomic changes associated with its pathway activation. In order to understand the CLDN10B pathway, its interactions and tumor-suppressive function, we performed mass spectrometry to identify its direct binding partners through GFP-Trap comparing overexpressed CLDN10B-GFP vs. GFP only. The identified proteins interacting with CLDN10B clearly separated GFP-only-interactors from the CLDN10B-GFP-interacting group by PCA (Suppl. Figure 18a, results listed in Suppl. Table 3). The proteins that are co-precipitated with CLDN10B-GFP formed a network analyzed by String and were color-coded for their GO-Term association (Suppl. Figure 18b and Suppl. Table 4). Interestingly, KRTs were not enriched among the significant binding partners of CLDN10B (Suppl. Figure 18c and Suppl. Figure 19). The protein network associated with overexpressed CLDN10B fused to GFP was predominantly linked to GO-terms related to mitochondrial organization, nucleocytoplasmic transport, and heat shock protein binding (Suppl. Table 4). Additionally, we identified a partial overlap between the proteins identified in our GFP-Trap CLDN10B experiment and CLDN10 interaction data from the IntAct database (Suppl. Table 5). The IntAct database provides information on CLDN10 binding/interaction partners, which we filtered for co-immunoprecipitation experiments, yielding results from the HEK293T and HCT116 cell lines, with notable differences. Five of the 11 proteins from IntAct overlapped with our immunoprecipitation, supporting the validity of our GFP-Trap method for CLDN10B. Interestingly, the alterations in the whole proteome under CLDN10B induction are not reflected with CLDN10B's binding partners. Additionally, overexpression of CLDN10 did not associate with GO-terms reflecting its membrane localization. In general, plasmid-based gene overexpression from strong promoters results in higher protein levels compared to the 'normal' levels generated by the endogenous promoter activation. As a result, we prioritized investigating CLDN10B function directly under its natural expression levels. Given the *CLDN10B* responsiveness to demethylation treatment (Fig. 3e) we employed our epigenetic editing approach using our CRISPR-dCas9 System under transient transfection [11–14]. We aimed to demethylate the *CLDN10B* CGI and restore its expression and tumor-suppressive function. Firstly, we designed and cloned single guide RNAs (sgRNA/guides/#) covering the *CLDN10B* CGI (six in total, Suppl. Figure 15a-b).

For editing we used the well-established inducer VP160 and dioxygenase TET1 as effectors coupled to the nuclease deficient Cas9 (dCas9), as earlier [12]. HEK293T cells were co-transfected with guides and dCas9-effectors as indicated. Interestingly, we observed that under non-guided conditions TET1 already induced moderate levels of *CLDN10B* (Fig. 6a), which was in line with earlier findings of TET1 off targets by systemic demethylation [53]. For the condition ‘non-guided’ we used a guide RNA sequence that does not correspond to any human sequence and serves as a non-specific gRNA. When inducing *CLDN10B*, both, the application of VP160 and TET1 demethylated *CLDN10B* (Fig. 6b). VP160 reduced *CLDN10B* methylation by 14%, while TET1 achieved a 17% reduction (Fig. 6b, base resolution Suppl. Figure 21a,b). This led to a 40-fold expression induction for VP160 (Fig. 6c), due to its direct transcriptional inducer function. The demethylation by TET1 led to a twofold expression induction of *CLDN10B* (Fig. 6c). Through a scratch/wound healing assay, we already demonstrated that *CLDN10B* induction resulted in decreased cell migration and slower wound closure (Fig. 4b). Similarly, under epigenetic editing, we found that reactivating *CLDN10B* using VP160 and TET1 also impaired cellular migration. Cellular migration was significantly reduced by 32% with VP160 treatment, while TET1 led to a 56% reduction (Fig. 6d, Suppl. Figure 20a). This greater effect is likely due to the nearly 60% slower cell migration in the non-guided control for TET1, compared to VP160 (Fig. 6d). Considering that CLDN10B plays a crucial role in forming tight junctions, thereby influencing cell–cell contacts and cellular polarity, we developed a dynamic 3D cell culture system (Suppl. Figure 20b). The 3D culture and spheroid formation more accurately replicate tumor architecture compared to traditional 2D cultures [54]. *CLDN10B* demethylation under CRISPR-editing was consistent across both 2D and 3D cultures, with a 13% reduction observed in 2D and a 5% reduction in 3D cultures (Fig. 6e, with base (paif) resolution Suppl. Figure 21c). However, demethylation was reduced in the 3D culture, likely due to the extended time frame and the use of our transient transfection assay. The induction of endogenous *CLDN10B* expression was stable, showing a 14-fold increase in 2D cultures and a 19-fold increase in 3D cultures (Fig. 6f). This upregulation significantly reduced spheroid size by 21% (Fig. 6g-h). It is important to note that VP160 has partial demethylation properties, which we have confirmed, and is also a well-known strong transcriptional activator, as demonstrated by the level of CLDN10B induction.

**Fig. 7:**
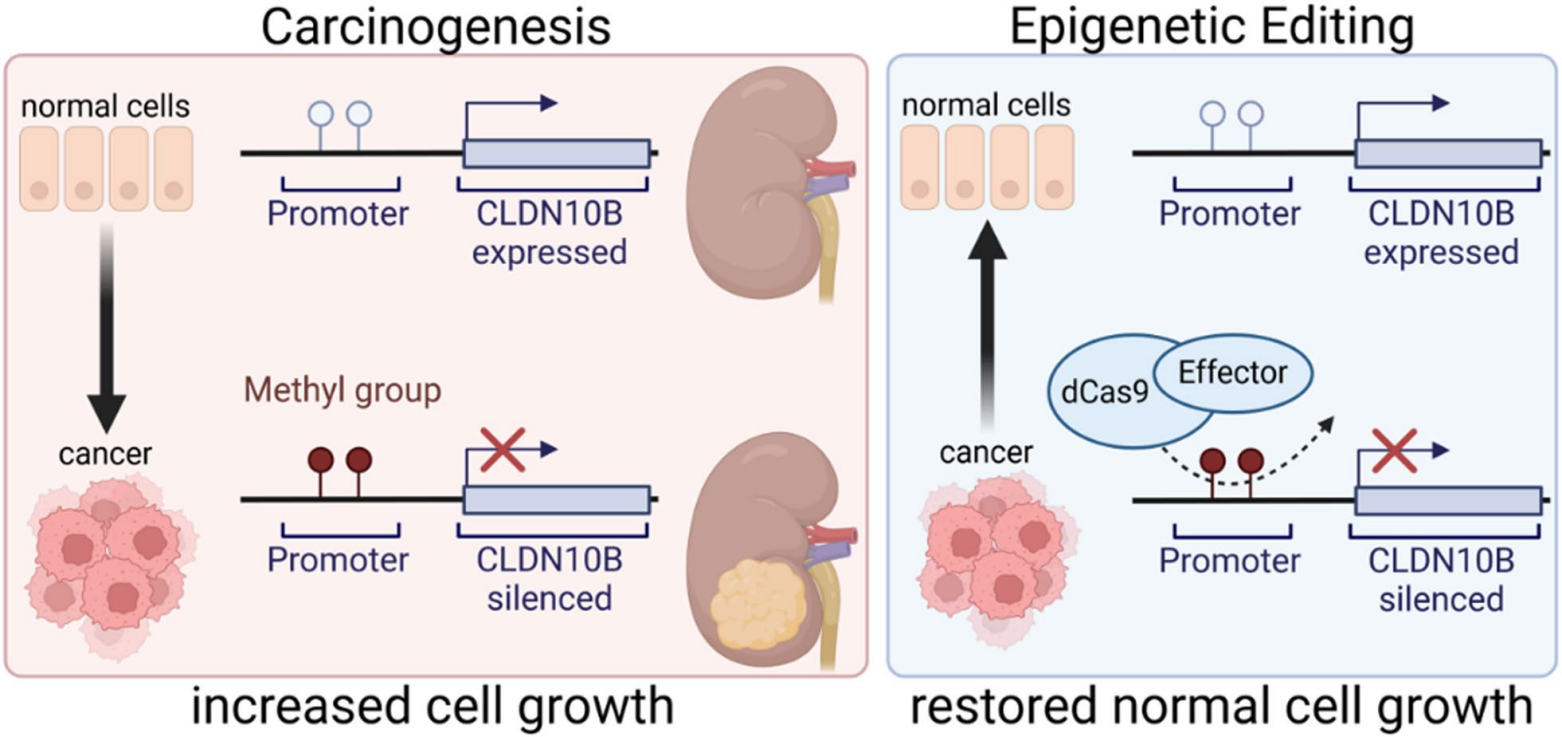
*CLDN10B* is silenced in kidney cancer, and its therapeutic reactivation is achievable. During carcinogenesis, hypermethylation of the CLDN10B promoter, particularly at its CpG island, occurs. This leads to the epigenetic inactivation and silencing of CLDN10B in kidney cancer and a loss of its tumor suppressor function. Using a CRISPR-dCas9 system, it is possible to reverse this epigenetic modification. By fusing dCas9 to effectors like TET1 (for demethylation) or VP160 (for both demethylation and transcriptional activation), targeted reactivation of CLDN10B can be achieved. Restoring *CLDN10B* expression suppresses cell growth

In summary (Fig. 7), our study shows that *CLDN10B* is frequently inactivated in renal cell carcinoma both pre- and post-transplantation. During the process of carcinogenesis, hypermethylation of the *CLDN10B* promoter occurs particularly within its CpG island, as an early event. This epigenetic modification of the DNA silences the *CLDN10B* gene, leading to a loss of its function as a tumor suppressor. As a result, CLDN10B becomes epigenetically silenced in kidney cancer, likely contributing to the progression of the disease. Reversing this hypermethylation and restoring *CLDN10B* expression is feasible through the use of advanced epigenetic editing techniques, such as the CRISPR-dCas9 system. This system can be engineered to target hypermethylated genes like *CLDN10B*. By fusing the catalytically inactive dCas9 to specific effector proteins, such as TET1 (which mediates DNA demethylation) or VP160 (which induces both demethylation and transcriptional activation), it is possible to precisely reactivate the CLDN10B. This targeted reactivation has been shown to restore CLDN10B expression, which in turn suppresses cell proliferation, offering a potential therapeutic strategy to combat kidney cancer.

## Discussion

The kidney tissues tubular system is composed of different segments: proximal, thick ascending limb Henle (TAL), distal, and collecting ducts. Its cells exhibit planar polarity, and its disruption contribute to diseases such as kidney cancer [55]. Clear cell renal cell carcinoma ccRCC arise from proximal tubular epithelial cells of the renal cortex and represent 75–80% of RCC. Papillary renal cell carcinoma pRCC arises from the renal tubular epithelium, shows a papillary growth pattern and comprises 10–15% of RCC [56]. A key element in maintaining cellular polarity is the formation of tight junctions [57], that anchor neighboring cells, regulate paracellular transport and communication, defines plasma membrane domains and facilitates cell–cell interactions [58]. Regulating tight junction formation is crucial for epithelial tissue structure and polarity; thus, loss of tight junction genes like *Claudins* would severely disrupt epithelial formation [59]. We identified Claudin 10 (*CLDN10B* isoform) to be epigenetically inactivated by DNA hypermethylation in renal cancer. We observed *CLDN10B* silencing in ccRCC cases under immunosuppression following kidney transplantation (post-transplantation malignancy, PTM) and in conventional clear cell renal cell carcinoma.. Even in our cohort of only five PT-ccRCC patient samples, the *CLDN10B* was the strongest differentially methylated gene. The use of patient-matched controls helps to mitigate this limitation. Additionally, PT-ccRCC is as rare as sporadic ccRCC, and the availability of samples remains limited. We are currently leaning toward the hypothesis that continuous immunosuppression accelerates carcinogenesis in potentially pre-existing lesions in individual kidney cells, which could explain the increased cancer susceptibility in immunosuppressed kidney transplant (KTx) patients. With our larger cohort PT-ccRCC, we hope to provide a more definitive answer to this question by comparing this specific cohort to conventional ccRCCs patients. Interestingly, CLDN10B was also hypermethylated in primary renal cancer of ccRCC and pRCC. This is supported by earlier reports on TCGA KIRC data that also found *CLDN10B* hypermethylation and *CLDN10* expression reduction [27]. However, we found that neither *CLDN10* nor *RASSF1A* were among the top 200 CpG biomarkers in ccRCC and pRCC (TCGA), possibly indicating the uniqueness of PT-ccRCC, which we are further investigating.

*CLDN10* gene is represented by two protein coding isoforms, both expressed in kidney (CLDN10A, CLDN10B). The role of splice variants of *CLDN10* or the antisense transcript are not part of the present study. Nevertheless, future CLDN10 research should take possible splice variants into account. It was reported that overexpressed CLDN10 splicing isoforms including exon 4 (containing the 4th transmembrane domain) generates proteins localized in the cytoplasmic membrane, while proteins produced from isoforms lacking exon 4 are retained in the endoplasmic reticulum [49]. However, this exon skipping event seems rare in humans, since there is no evidence for exon 4 skipping in publically available human RNA-Seq data. The CLDN10 antisense transcript may add another layer of regulation beyond promoter hypermethylation, to be explored in future studies. We found that *CLDN10* hypermethylation only occurs on the B isoform, whereas the A isoform is hypomethylated in PT-ccRCC and conventional/sporadic ccRCC and pRCC. This inverse methylation pattern was also consistent in ccRCC cell lines and identifies renal from other cancer cell lines. It could be speculated that during carcinogenesis *CLDN10B* becomes hypermethylated and silenced, whereas *CLDN10A* is hypomethylated and induced expression. It will be interesting to study the functional link between hypermethylation of *CLDN10B* and how its loss leads to induction of *CLDN10A*. Further studies will clarify if CLDN10A compensates for CLDN10B loss. Future studies should explore whether our observation is specific to RCCs from the TAL, where CLDN10B is mainly expressed [60]. In chromophobe RCC subtype, which originates from the cortical collecting ducts [61], we did not observe hypermethylation of *CLDN10B*, and only partial hypomethylation of the A isoform, which is in line with the predominant CLDN10B expression in the TAL and CLDN10 in proximal tubules. The observation of CLDN10B loss by hypermethylation in ccRCC leads us to speculate on its diverse function in different regions of the kidney tubular system. CLDN10B in the proximal tubule would rather plays a role in maintaining tissue integrity as a tight junction protein connecting neighboring cells. Its loss contributes to ccRCC formation, especially when combined with VHL loss, a key driver of ccRCC. In contrast, CLDN10B expression in the thick ascending limb (TAL) regulates ion selectivity, and its hypermethylation might also impair this function. However, since TAL cells do not typically give rise to ccRCC, this CLDN10B dysfunction in the TAL is unlikely to contribute to tumorigenesis. *CLDN10B* mutations are clinically significant, causing the rare recessive HELIX syndrome, a type of Claudinopathy. Although HELIX syndrome patients, mostly children and young adults, have not shown increased RCC susceptibility, with only 20 cases reported, it remains unclear whether they may develop RCC later, particularly under immunosuppressive conditions. In renal cancer, mutation of CLDN10 is rare and hypermethylation likely causes a loss of function of the B isoform. Expression analysis of CLDN10 using databases like TCGA (KIRC-ccRCC, KIRP-pRCC) can be misleading, as they do not necessarily distinguish between the A and B isoforms, both of which are expressed in kidney tissue. Nevertheless, and clinically relevant, we and others [27] observed loss of *CLDN10* expression is associated with decreased patient survival and advanced tumor staging in ccRCC, irrespective of isoform levels. This was consistent with previous research indicating *CLDN10* as a RCC biomarker [25, 27]. Distinguishing between the isoforms is essential to understand the epigenetic silencing of CLDN10B. Clinically, we found that hypermethylation of CpG probes of *CLDN10B* correlated with reduced survival for ccRCC. *CLDN10B* hypermethylation extends across its CGI and into regions with high CpG content (shore and shelf), found in primary renal clear cell carcinoma, papillary cell carcinoma, and in PT-ccRCC. *CLDN10B* expression was restored with pharmacological demethylation (5-Aza-2’deoxycytidine), suggesting its epigenetic regulation as a potential therapeutic target for cancer. We first established its tumor-suppressive role in conventional 2D cell cultures and in 3D cultures in the HEK cell model. Overexpressing CLDN10B in HEK293T was detectable along the cell membrane by immunofluorescence, in line with its tight junction formation. We observed CLDN10B growth suppressive function by reduced wound healing and cell cycle arrest in S phase and in reduced spheroid size in 3D culture. The immortal renal cell line HEK, while a superior model for transfection, is not a perfect representation of renal cancer subtypes. Follow-up studies should therefore include renal cell lines from ccRCC or pRCC to validate CLDN10B's growth-suppressive role. Nevertheless, HEK cells exhibit a similar epigenetic silencing of many tumor suppressor genes, making them a useful model for epigenetic modulation. *CLDN10B* induction altered the mRNA expression pattern toward GO-terms of ‘cell adhesion’ and ‘extracellular matrix’ and the GO-term ‘regulation of cell death’. However, it must be mentioned that we did not observe apoptosis induction by subG1 phase in our cell cycle analysis. Plasmid-based overexpression or inducible cell lines often produce high protein levels, which may not accurately reflect tumor suppressors behavior like CLDN10B. Detecting CLDN10B protein is challenging due to non-specific results of commercial antibodies. To study CLDN10B in its natural context, we used an approach based on its endogenous induction and reactivation. We identified putative E-boxes of the *CLDN10B* promoter that could serve as binding motifs for transcription factors. Using a direct CRISPR-Cas9 approach, we induced *CLDN10B* by targeting E-boxes, possibly generating indels. We speculate that this is due to the loss of repressive factors, binding to the E-Box that silence *CLDN10B*. This endogenous CLDN10B induction was associated with distinct proteomic changes indicative of a shift toward cellular polarity, supporting CLDN10B as a regulator of cell polarity or even epithelial organization. Interestingly, the CLDN10B binding partners and their network differ from the proteomic changes observed upon CLDN10B induction. The protein network associated with overexpressed CLDN10B is linked to heat shock protein binding, mitochondrial function, and cellular transport. We identified more co-precipitating partners for CLDN10B than those listed in the IntAct database, with a partial overlap that supports our approach. Although CLDN10B localizes to the membrane, it still interacts with proteins related to mitochondria and cellular transport, consistent with a previous report highlighting a role for CLDN10B in mitochondria [26]. However, we believe that overexpression approaches has its limitations. Overexpressing CLDN10B may identify non-representative partners that do not reflect its natural interactions. We conclude that correct dosing of CLDN10B is crucial for understanding its endogenous function. Overinduction or overexpression may not accurately represent natural levels. Consequently, we prioritized analyzing endogenous levels through our epigenetic editing approach and reversed the epigenetic silencing of CLDN10B to explore its tumor-suppressive function in an endogenous context. However, we observed relevant limitations with the dCas9-TET1 system, particularly due to off-target effects. Overexpression of TET1, even without specific targeting, triggered *CLDN10B* expression induction, which aligns with our previous findings on TET1 inducing ubiquitous demethylation of other target genes [11] and is furthermore also consistent with other studies on TET use [53]. This effect is likely linked to the activity of TET dioxygenases, which trigger a gradual demethylation process. Consequently, overexpressing TET1 replicates the effects of systemic demethylation treatments, such as Aza, and should therefore be employed sparingly. Our unpublished data demonstrated the specificity of CLDN10 CRISPR-dCas targeting using the guides employed in this study, with no significant off-target effects detected through whole methylome EPIC2 analysis. Additionally, we confirmed that the guides used for CLDN10 targeting do not bind to other genomic sites. Nevertheless, targeting the *CLDN10B* promoter with VP160 and TET1 in the dCas9 system not only demethylated and reactivated *CLDN10B* but also restored its tumor-suppressive function in a wound healing assay. VP160 proved more effective, likely due to its additional role as a transcriptional activator, increasing *CLDN10B* levels strongly. Both our observations and previous reports suggest that dCas9-VP160 has the ability to demethylate genomic regions through steric hindrance of DNMT activity [53]. Given TET1’s limited impact on *CLDN10B* induction and the elevated baseline expression in its presence, we used dCas9-VP160 and demonstrate CLDN10B’s tumor-suppressive effects also in the 3D spheroid model. Building on our hypothesis that CLDN10B functions as a tumor suppressor in renal cancer, we now confirm its epigenetic silencing in renal cancer. Our renal cell line study further supports its tumor-suppressive role by demonstrating its growth-inhibitory function. Future studies are underway to further establish its tumor suppressor function in renal cancer. The kidney’s tubular system depends on cellular polarity and tight junctions, and disruptions can lead to diseases like kidney cancer. In this study, we discovered that Claudin 10B (*CLDN10B*) is epigenetically silenced by DNA hypermethylation in renal cancers, including clear cell renal cell carcinoma (ccRCC), papillary renal carcinoma (pRCC) and post-transplant ccRCC, whereas *CLDN10A* becomes hypomethylated. The *CLDN10B* silencing was associated with poor patient survival and advanced tumors. We demonstrated that CLDN10B functions as a tumor suppressor, inhibiting cellular growth. By using a gene-specific CRISPR-based epigenetic editing strategy, we successfully reactivated endogenous CLDN10B, restoring its tumor-suppressive function. Our findings highlight CLDN10B as a promising therapeutic target in renal cancer.

## Supplements

Methods Details on mass spectrometry-based proteomics. For whole proteome analysis upon CLDN10 induction cells were resuspended in 4% sodium lauroyl sarcosinate (SLS) in 100 mM Tris–HCl (pH ~ 7.5) solution and boiled for 10 min at 90 °C in a ThermoMixer (1800 rpm). Supernatants were collected by 13,000 g centrifugation at 4 °C for 15 min and protein concentration was estimated by BCA Assay. Prior to protein digestion, DNA was removed by two cycles of incubation at 90 °C in a ThermoMixer (10 min, 1800 rpm) followed by 1 min sonication and protein content was estimated with Pierce™ BCA Protein Assay Kit. Approximately 50 μg of protein (approx. 50 μl of solution) were taken for further processing. Proteins from the CLDN10 interaction partner analysis by GFP-Trap, were eluted with 0.2 M Glycine pH 2.5 and Neutral. Buffer: Tris 1 M pH10.4 and snap frozen. Protein denaturation was initiated by addition of 20 μl of 8% sodium lauroyl sarcosinate (SLS) in 100 mM Tris–HCl (pH ~ 7.5) solution and boiled for 10 min at 90 °C in a ThermoMixer (1800 rpm).

All samples were reduced by the addition of 400 mM dithiothreitol to a final concentration of 10 mM and incubation at 95 °C for 10 min, followed by alkylation by iodoacetamide at a final concentration of 13 mM and incubation for 30 min at 25 °C. A modified version of the SP3 method [41] was used for further sample preparation on an in-house made magnetic rack. Protein binding was performed in a final concentration of 70% anhydrous acetonitrile (ACN) solution at neutral pH with subsequent washes with 70% ethanol and 100% anhydrous acetonitrile. After acetonitrile removal, beads were resuspended in 50 μl of 50 mM TEAB buffer and 1 ug of trypsin (Promega, Madison, Wisconsin, USA) was added. Protein digestion was performed overnight, at 37 °C with shaking and was stopped by addition of TFA to a final concentration of 1.5%. Peptides were purified using solid phase extraction on C18 microspin columns according to the manufacturer's instructions (Macherey–Nagel, Germany), based on the original protocol [42]. Purified peptides were first dried and then resuspended in 50 μL of 0.1% TFA. Peptide concentration was estimated using the Pierce Fluorimetric Peptide Assay, and sample volumes were adjusted to achieve equal concentrations.

Peptides for the whole proteome analysis upon CLDN10 induction were analyzed by liquid chromatography−mass spectrometry (MS) carried out on a Exploris 480 instrument connected to an Ultimate 3000 rapid separation liquid chromatography (RSLC) nano instrument and a nanospray flex ion source (all Thermo Fisher Scientific, Waltham, USA). Peptide separation was performed on a reverse-phase high-performance liquid chromatography (HPLC) column (75 μm by 42 cm) packed in-house with C18 resin (2.4 μm; Dr. Maisch HPLC GmbH, Ammerbuch, Germany). Around 500 ng of peptides were first loaded onto a C18 precolumn (preconcentration setup) and then eluted in the backflush mode with a gradient from 98% solvent A (0.15% formic acid) and 2% solvent B (99.85% acetonitrile and 0.15% formic acid) to 25% solvent B over 65 min, continued from 25 to 35% of solvent B for another 24 min. The flow rate was set to 300 nL/min. The data were acquired in a data-independent mode (DIA) for the initial label-free quantification, and study was set to obtain one high-resolution MS scan at a resolution of 120,000 (m/z 200) with the scanning range from 350 to 1400 m/z followed by DIA scans with 45 fixed DIA windows with the width of 14 m/z (1 m/z overlap from neighboring windows), ranging from 320 to 950 m/z at a resolution of 15,000. The automatic gain control was set to 300% for MS survey scans and 3000% for DIA scans.

Purified peptides from the CLDN10 interactomics were analyzed by liquid chromatography–tandem mass spectrometry (MS) carried out on a Bruker Daltonics timsTOF Ultra instrument connected to a Bruker Daltonics nanoElute instrument. Approximately 50 ng of peptides were loaded onto a C18 precolumn (Thermo Trap Cartridge 5 mm, µ-Precolumn TM Cartridge/PepMap TM C18, Thermo Scientific) and then eluted in the backflush mode with a gradient from 98% solvent A (0.15% formic acid) and 2% solvent B (99.85% acetonitrile and 0.15% formic acid) to 17% solvent B over 36 min, continued from 17 to 25% of solvent B for another 18, then from 25 to 35% of solvent B for another 6 min over a reverse-phase high-performance liquid chromatography (HPLC) separation column (PepSep Ultra, C18, 1.5 μm, 75 μm × 25 cm, Bruker Daltonics) with a flow rate of 300 nL/min. The outlet of the analytical column was coupled to the MS instrument by CaptiveSpray 20 μm Emitter. Data were acquired using a data-independent acquisition (DIA) paradigm using a default method provided by Bruker. In short, spectra were acquired with fixed resolution of 45,000 and mass range from 100 to 1700 m/z for the precursor ion spectra and a1/k0 range from 0.64 to 1.45 V s/cm2 with 100 ms ramp time for ion mobility, followed by DIA scans with 24 fixed DIA windows of 25 m/z width, ranging from 400 to 1000 m/z.

Peptide spectrum matching and label-free quantitation were subsequently performed using DIA-NN [43] and a library-free search against the Human Uniprot.org database (20,407 reviewed Swiss-Prot entries; April 2023). In brief, output was filtered to a 1% false discovery rate on precursor level. Deep learning was used to generate an in silico spectral library for library-free search. Fragment m/z was set to a minimum of 200 and a maximum of 1,800. In silico peptide generation allowed for N-terminal methionine excision, tryptic cleavage following K*,R*, a maximum of one missed cleavage, as well as a peptide length requirement of seven amino acid minimum and a maximum of 30. Cysteine carbamidomethylation was included as a fixed modification and methionine oxidation (maximum of two) as a variable modification. Precursor masses from 300 to 1800 m/z and charge states one to four were considered. DIA-NN was instructed to optimize mass accuracy separately for each acquisition analyzed and protein sample matrices were filtered using a run-specific protein q-value (“–matrix-spec-q” option). Downstream data processing and statistical analysis were carried out by the Autonomics package developed in-house (version 1.13.19). Proteins with a q-value of < 0.01 were included for further analysis.

MaxLFQ [62] values were used for quantitation of the whole proteome analysis upon CLDN10 induction. DIA-NN spectral identification software initially identified 8185 protein groups. After dropping 24 without replication (within the subgroup), and filtering out 1356 proteins with less than 2 precursors identified, 6805 protein groups were retained for further analysis. Differential abundance of protein groups was evaluated by Autonomics employing Bayesian moderated t-test as implemented by limma [63].

For the interactomics study, raw intensities were used instead of normalized values. DIA-NN software identified 7396 protein groups. After dropping 153 without replication (within subgroup), and filtering out 2507 proteins with less than 2 peptides identified, 4736 protein groups were retained for further analysis. Systematic missing values (missing completely in some subgroups but detected in others (for at least half of the samples) were imputed by introducing intensities that mimics a lower value of detection of a mass spectrometer and are randomly chosen from a narrow distribution of values. Differential abundance of protein groups was evaluated by Autonomics employing Bayesian moderated t-test as implemented by limma.

The full list of spectral matching settings can be found in the “…report.log.txt”, full code for data processing and statistical analysis are uploaded along with the mass spectrometric raw data to the ProteomeXchange Consortium with dataset identifier: PXD056422, via the MassIVE partner repository (https://massive.ucsd.edu/, MassIVE-ID: MSV000095998; 10.25345/C5NK36H32:).[v6].

## Supplementary Information

Supplementary Material.

## Data Availability

Data described in the manuscript will be available for download at indicated repositories (please see MM section). The datasets are available in the R2 Genomics Analysis and Visualization Platform and repository under ‘PTM’ https://hgserver1.amc.nl/cgi-bin/r2/main.cgi?open_page = login and the mass spectrometric data in the ProteomeXchange Consortium with dataset identifier: PXD056422, via the MassIVE partner repository (https://massive.ucsd.edu/, MassIVE-ID: MSV000095998; 10.25345/C5NK36H32:) and transcriptomic data in the NCBI's Gene expression omnibus under the accession GSE283785 (https://www.ncbi.nlm.nih.gov/geo/query/acc.cgi?acc = GSE283785).
