# Supplementary material for: Epigenetic silencing and CRISPR-mediated reactivation of tight junction protein claudin10b (CLDN10B) in renal cancer
Source: Clin Epigenetics. 2025 Jun 16;17:102. doi: 10.1186/s13148-025-01911-2 (PMC12172364; doi:10.1186/s13148-025-01911-2)
Supplement: Supplementary file 1 — Supplementary Material. [file 13148_2025_1911_MOESM1_ESM.pdf]

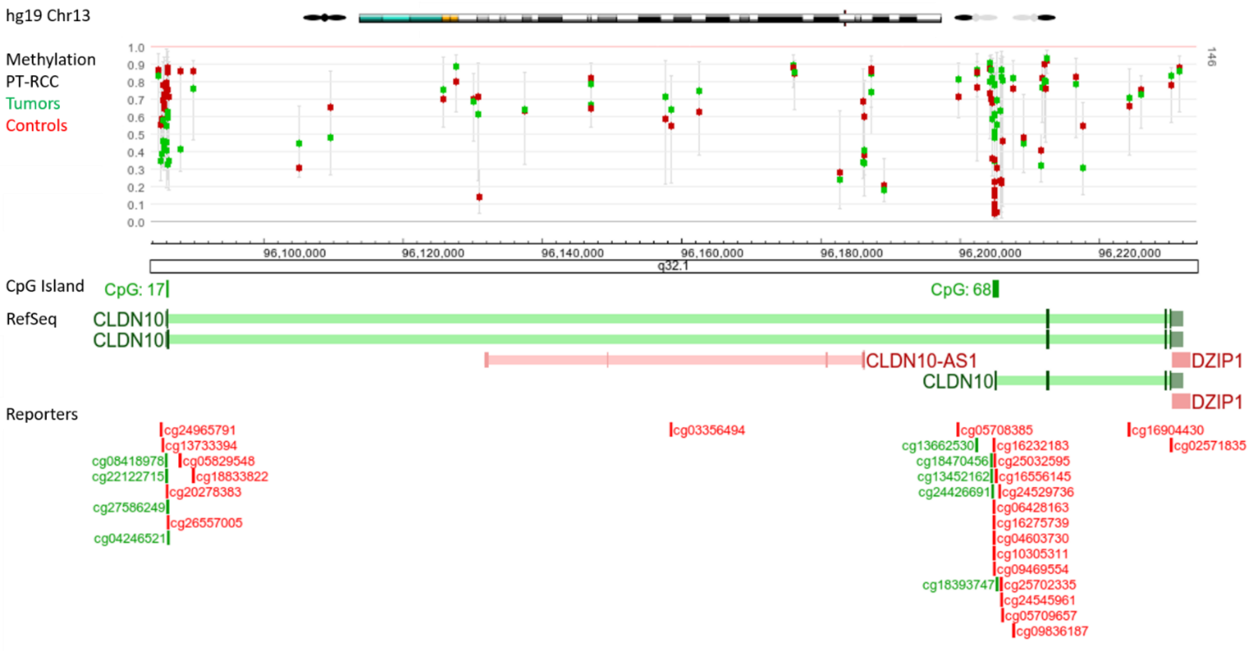

Suppl. Fig. 1. Genomic organization of CLDN10. *CLDN10 is located on chromosome 13 and shown with its transcripts isoform A, isoform B and antisense transcript. Only isoform A and isoform B harbor a CpG island (17 CpGs for A isoform, 68 CpGs for B isoform). Methylation probes from 450k array (Illumina) are mostly positioned across promoters/CGIs. Analyzed by R2 Genome Browser.*

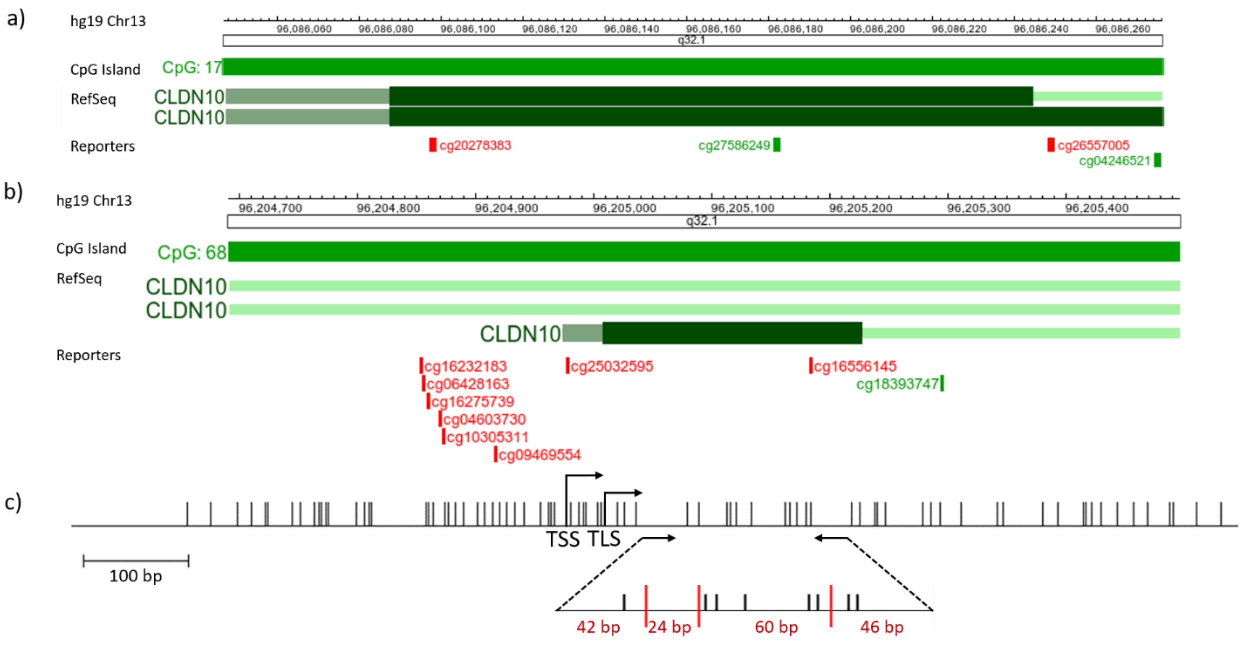

Suppl. Fig. 2. Genomic organization of CLDN10A and CLDN10B. *a) Isoform A harbors a CpG island with only 17 CpGs and is shown with methylation probes from 450k array (Illumina) within CGIs. b) Isoform B harbors a CpG island with 68 CpGs and is shown with methylation probes from 450k array (Illumina) within CGIs. c) Details for CLDN10B methylation analysis by CoBRA with genomic CpGs (vertical lines) relative to transcriptional start site (TSS) and translational start site (TLS) (bent arrow) and horizontal arrows indicate COBRA PCR product (172 bp) and magnification of COBRA restriction analysis (with Taq1, with digestions products in red).*

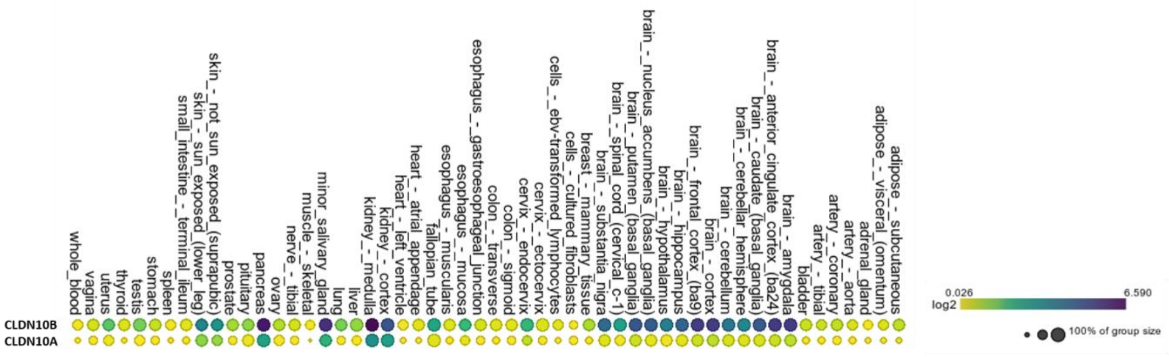

Suppl. Fig. 3. Isoform specific expression CLDN10 across normal tissues. *Expression analysis of CLDN10 across normal tissue from GTeX v8 (isoform) with tissue details from n=17382 (tmp, analyezd by R2) with identifier for isoforms CLDN10A ENS00000376873.7 and CLDN10B ENST00000299339.2.*

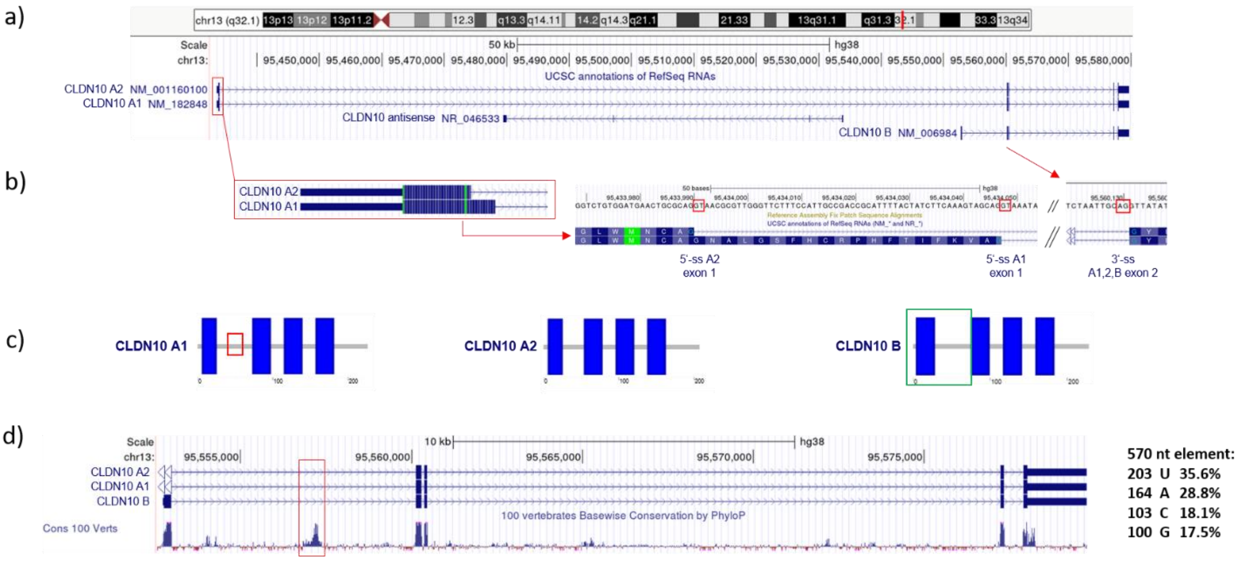

Suppl. Fig. 4. Isoform analysis of CLDN10. *UCSC reference sequence annotation on GRCh38/hg368 genome. a) CLDN10 isoform overview displaying two A isoforms (A1 and A2), the antisense CLDN10 RNA, as well as isoform B. b) enlargement of the exon 1 alternative donor or 5'-splice sites (ss) of isoforms A1 and A2, as well as the acceptor or 3'-splice site of all three isoforms A1, A2 and B. Nucleotide and amino acid sequences are displayed, with conserved intronic dinucleotides characteristic of canonical splice sites highlighted. Note that 57 nt or 19 amino acids are added in isoform A1 compared to A2. c) SMART protein domain analysis. All isoforms encode four transmembrane domains (blue boxes). The additional 19 amino acids in isoform A1 add to the extracellular domain between transmembrane domain 1 and 2 (based on dbPTM predictions; see also Suppl. Fig. 10). CLDN10 isoform B sequence is completely different in transmembrane domain 1 and the first extracellular domain (highlighted by green box, left panel). d) Intron 1 of all isoforms contains a 507 nt sequence element which is highly conserved between 100 vertebrate species, suggesting a functional role. The element is enriched in uridine nucleotides.*

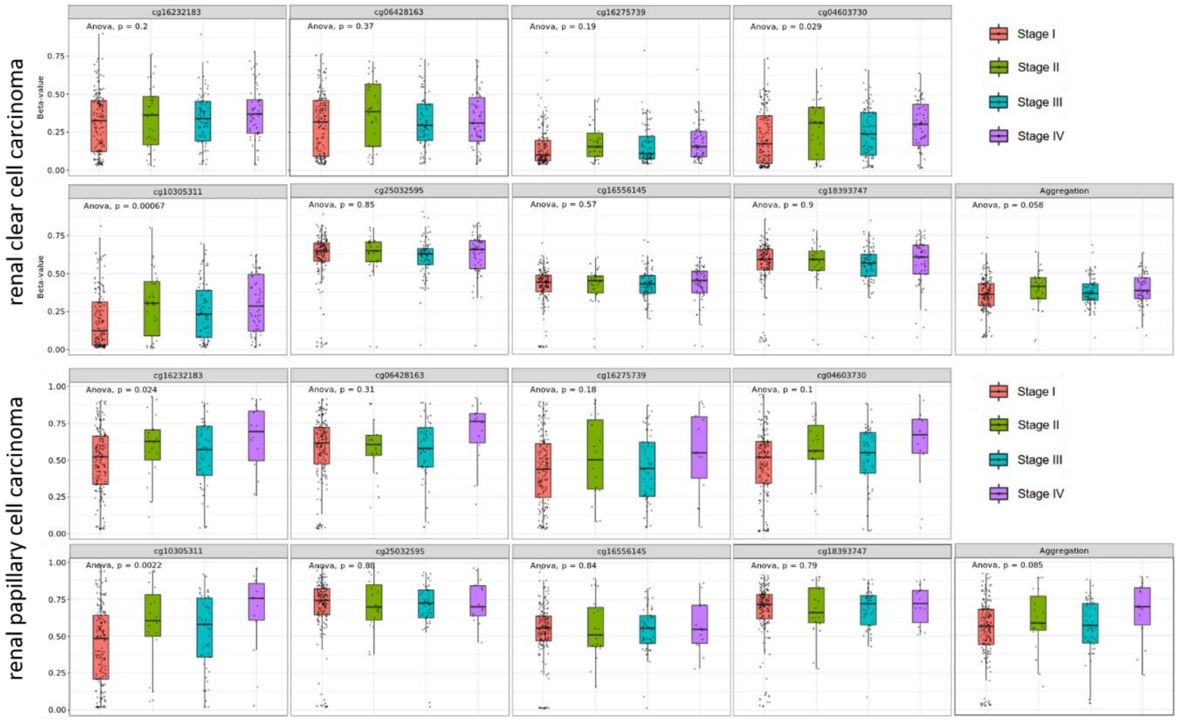

Suppl. Fig. 5. Correlation analysis of CLDN10B hypermethylation with tumor stage in renal clear cell and papillary cell carcinoma. *Methylation levels of CLDN10 CGI probes cg16232183, cg06428163, cg16275739, cg04603730, cg10305311, cg25032595, cg16556145 and cg18393747 for tumor stages I to IV reveals significantly increasing methylation for cg04603730 and cg10305311 in ccRCC (KIRC) and cg16232183 and cg10305311 for pRCC (KIRP). Analysis by SMART shown as ßvalue and methylation aggregation by mean.*

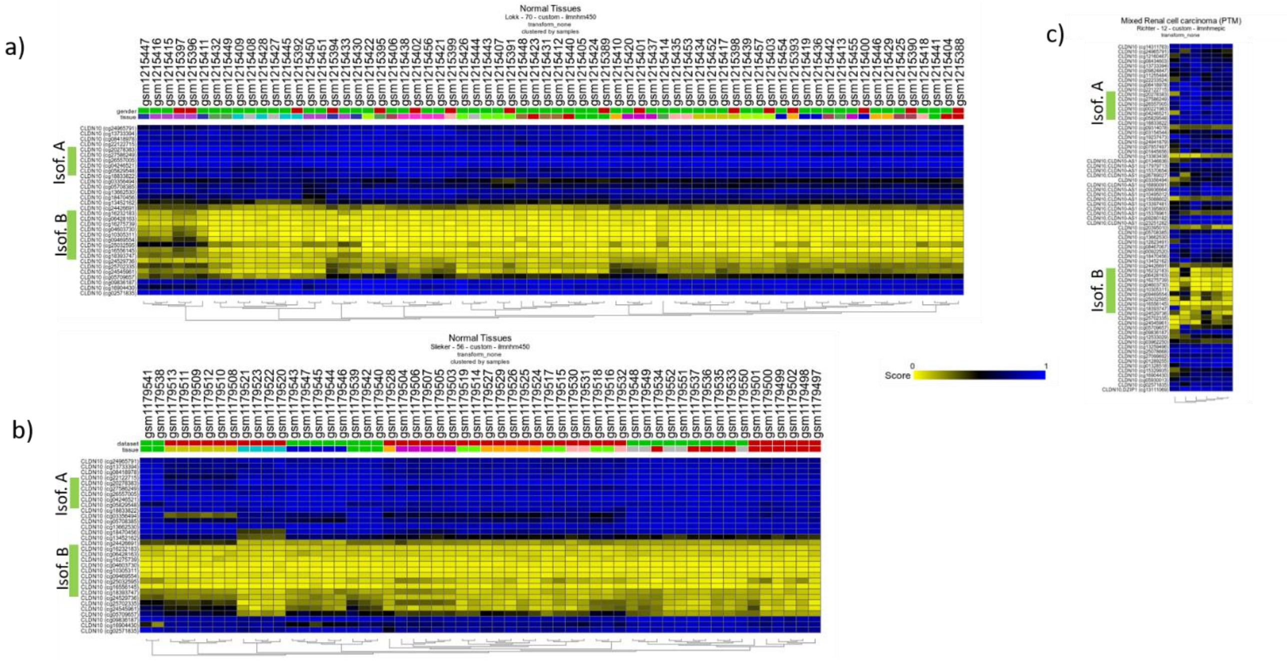

Suppl. Fig. 6. Across normal tissues the promoter of CLDN10A is methylated and CLDN10B unmethylated. *Uniform methylation pattern of the CLND10 promoters across normal tissues with Isoform A methylated (blue) and CLDN10 unmethylated (yellow) (a+b; 450k array, datasets Slieker n=56, Lokk n=70, analyzed by R2). c) Uniform methylation pattern of CLDN10 also in normal renal tissue from PTM patients (EPIC1 array, dataset Richter, n=6, analyzed by R2). CGIs of isoforms are marked with green boxes. EPIC array (c) contains additional CLDN10 probes, including its antisense transcript.*

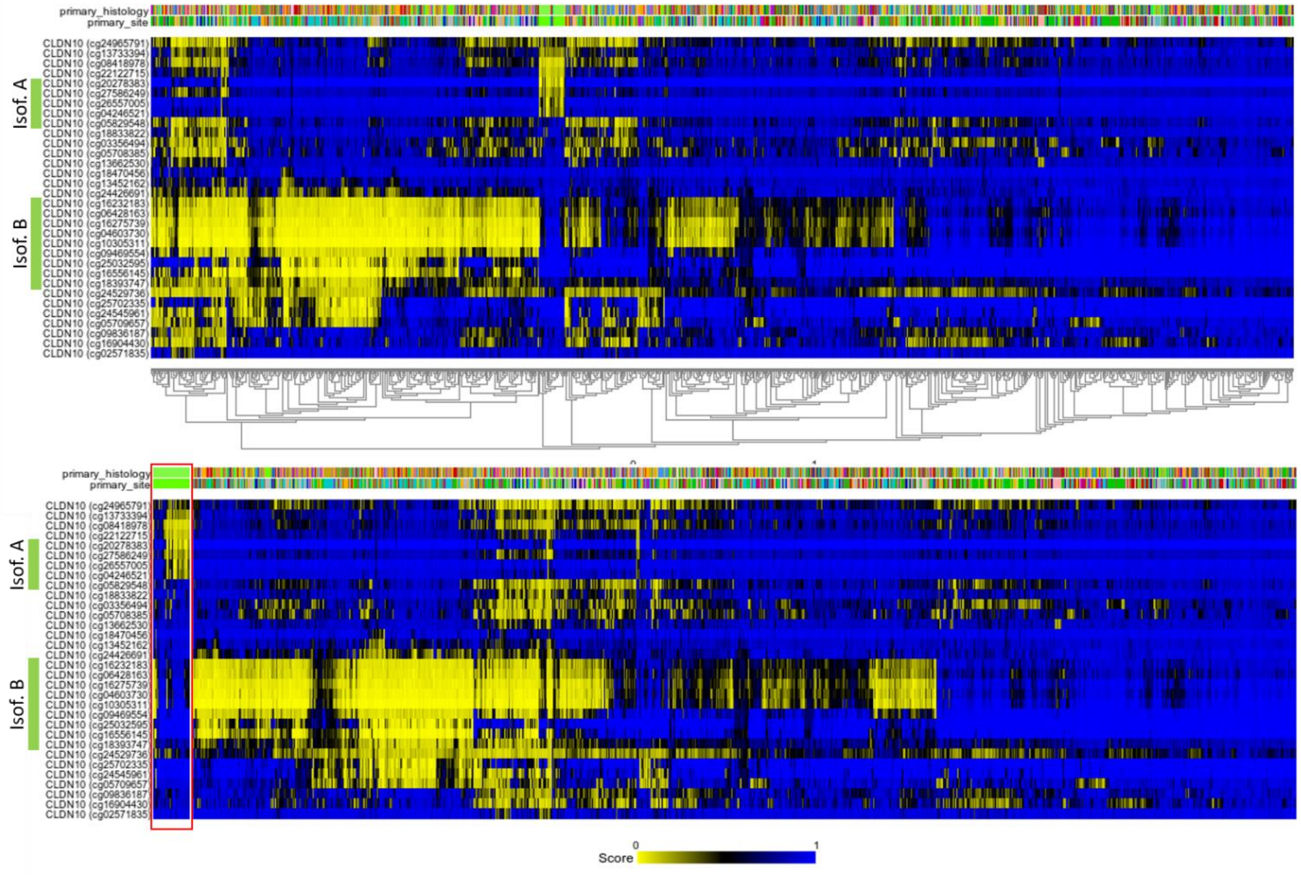

Suppl. Fig. 7. Differential methylation of CLDN10 isoforms identifies kidney cancer amongst various cancer cell lines. *Methylation pattern of CLND10 by methylome (450k array, datasets Esteller n=1028, analyzed by R2). CGI of isoforms is marked with green box. Upper heatmap is clustered by methylation pattern and lower heatmap additionally split by tissue (primary site kidney marked in red).*

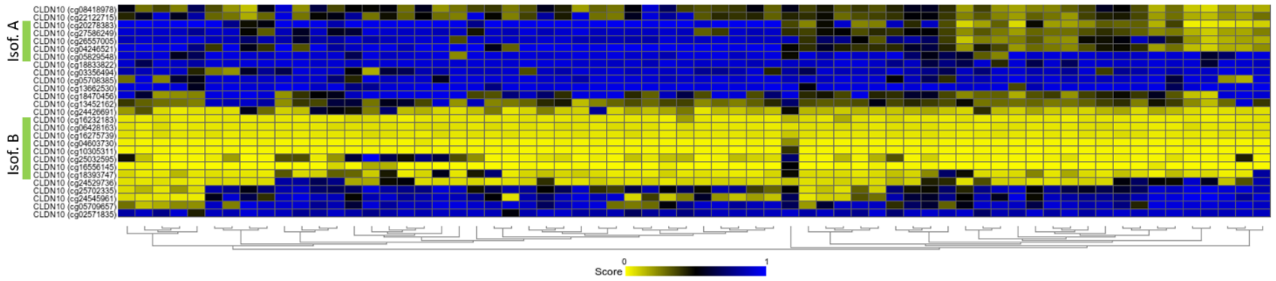

***Suppl. Fig. 8. Partial hypomethylation of CLDN10 isoform A, but not hypermethylation of CLDN10B in chromophobe renal cell carcinoma**. **a)** Methylation pattern of CLND10 by methylome (450k array, datasets TCGA n=66, analyzed by R2). CGI of isoforms is marked with green box.*

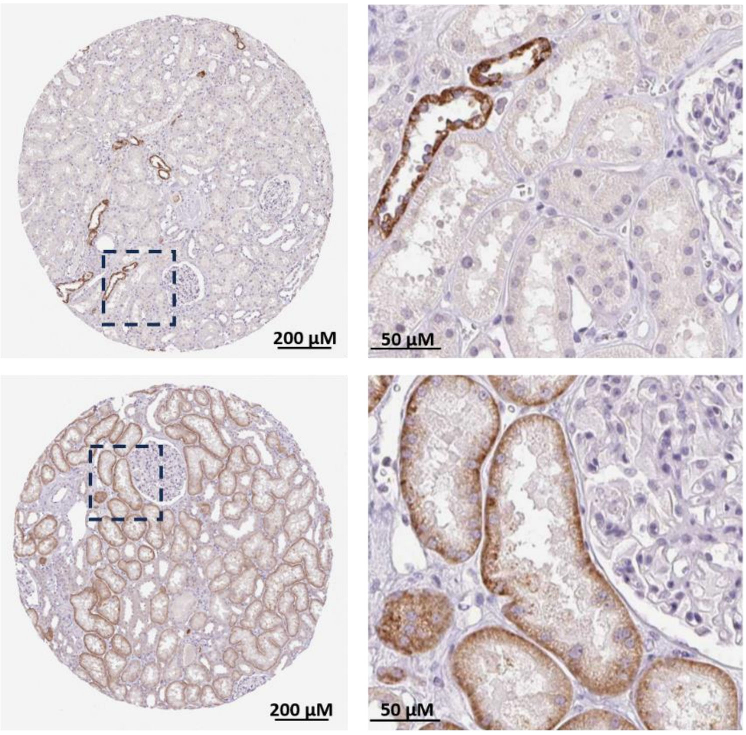

Suppl. Fig. 9. Protein expression of CLDN10 in human kidney. *Expression profile of CLDN10 across kidney sections for two patients shows CLDN10 is mostly present in tubulus system of the kidney. Magnification is shown on the right. Samples are: CAB012969 Female age 41, patient 2530, cells in glomeruli: staining was not detected. In cells in tubules the staining was medium, intensity was strong, quantity <25% and location was cytoplasmic/ membranous. HPA042348 Male age 61, patient 1859, cells in glomeruli: staining was not detected. In cells in proximal tubules the staining was medium, intensity was moderate, quantity >75% and location was cytoplasmic/ membranous. Data/sections for expression profile from The Human Protein Atlas.*

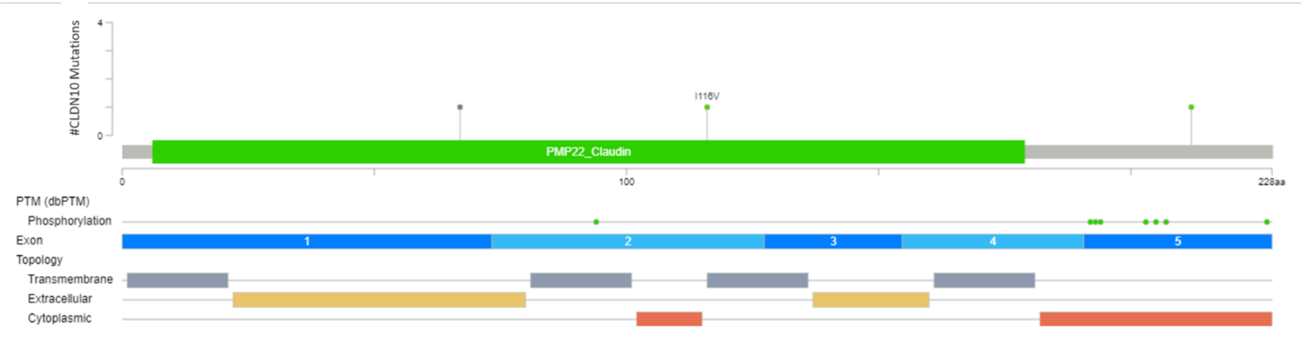

Suppl. Fig. 10. CLDN10 is rarely mutated (0.4 %) in RCC. *a) Schematic overview of CLDN10 analyzed with cbioportal shows location of mutations for available RCC data sets of 1814 samples / 1744 patients in 7 studies (green missense, grey truncating and orange splice mutations; all of unknown significance. Phosphorylation sites are annotated in green, exon structure in blue and topology with transmembrane, extracellular and cytoplasmic subcellular location of the mature protein.*

Suppl. Fig. 10. CLDN10 is rarely mutated (0.4 %) in RCC. *a) Schematic overview of CLDN10 analyzed with cbioportal shows location of mutations for available RCC data sets of 1814 samples / 1744 patients in 7 studies (green missense, grey truncating and orange splice mutations; all of unknown significance. Phosphorylation sites are annotated in green, exon structure in blue and topology with transmembrane, extracellular and cytoplasmic subcellular location of the mature protein.*

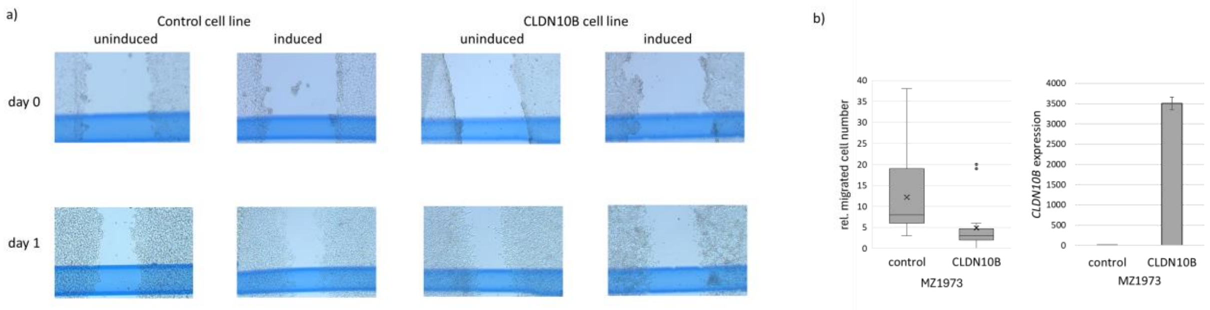

Suppl. Fig. 11. CLDN10B induction slows wound/scratch closure in 2D cell culture and reduces transwell migration. *a) HEK Trex cell line (control and CLDN10B-inducible) were seeded at same cell number, grown to confluency in 2D culture, both cell lines were mock and doxycycline induced (2 lines, 2 conditions), wound/scratch was placed and position marked (blue). Wound/scratch was determined on day 0 and after 24h at exact same position for n=36 positions along scratch line. According quantification is shown in Fig. 4 e). b) MZ1973 ccRCC cell line was transfected with CLDN10B expression vector, positive cells were selected with puromycin (0,25µg/ml for co-transfected puromycin resistance containing plasmid) and cells were counted and equal cell numbers were place in transwell migration chamber with 5% FCS in upper chamber and 10% FCS in lower chamber. Migration was measured 24h later from migrated cells in lower chamber. Experiment was performed in comparison to empty vector transfected cells under equal conditions. 6 different transwells were calculated and mean and SD are shown. In addition, overexpression of CLDN10B was quantified by RT PCR from very same experiment.*

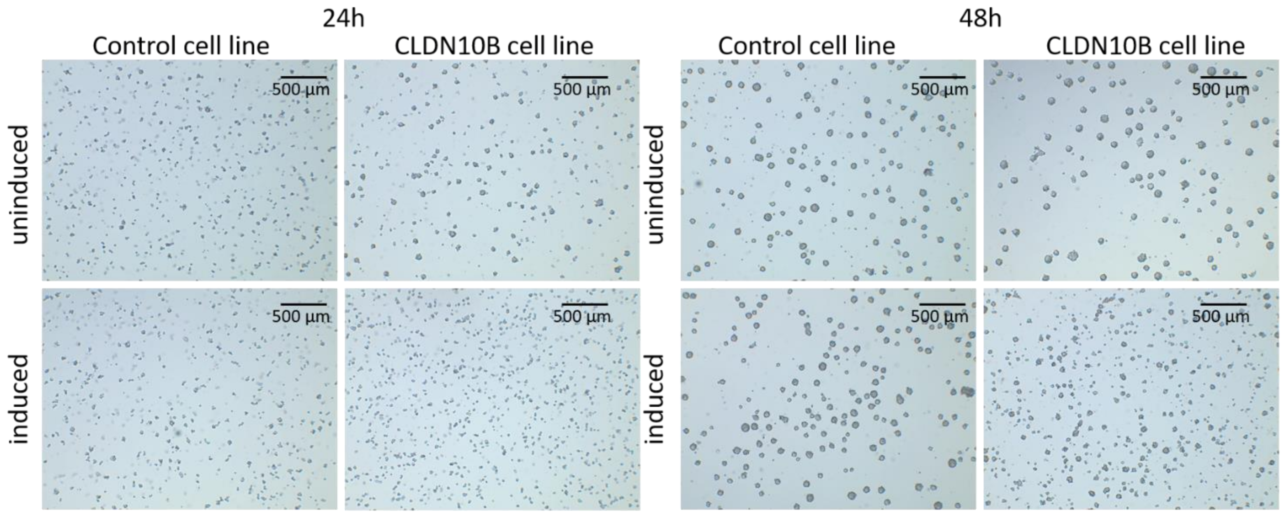

Suppl. Fig. 12: Visualization of reduced spheroid formation upon CLDN10B induction in dynamic 3D culture. *3D Spheroids of control and CLDN10B inducible cell line under uninduced and induced conditions (HEK Trex) are shown after Dox stimulation at 24h and 48h. Picture equivalent as Fig. 4f; detailed view.*

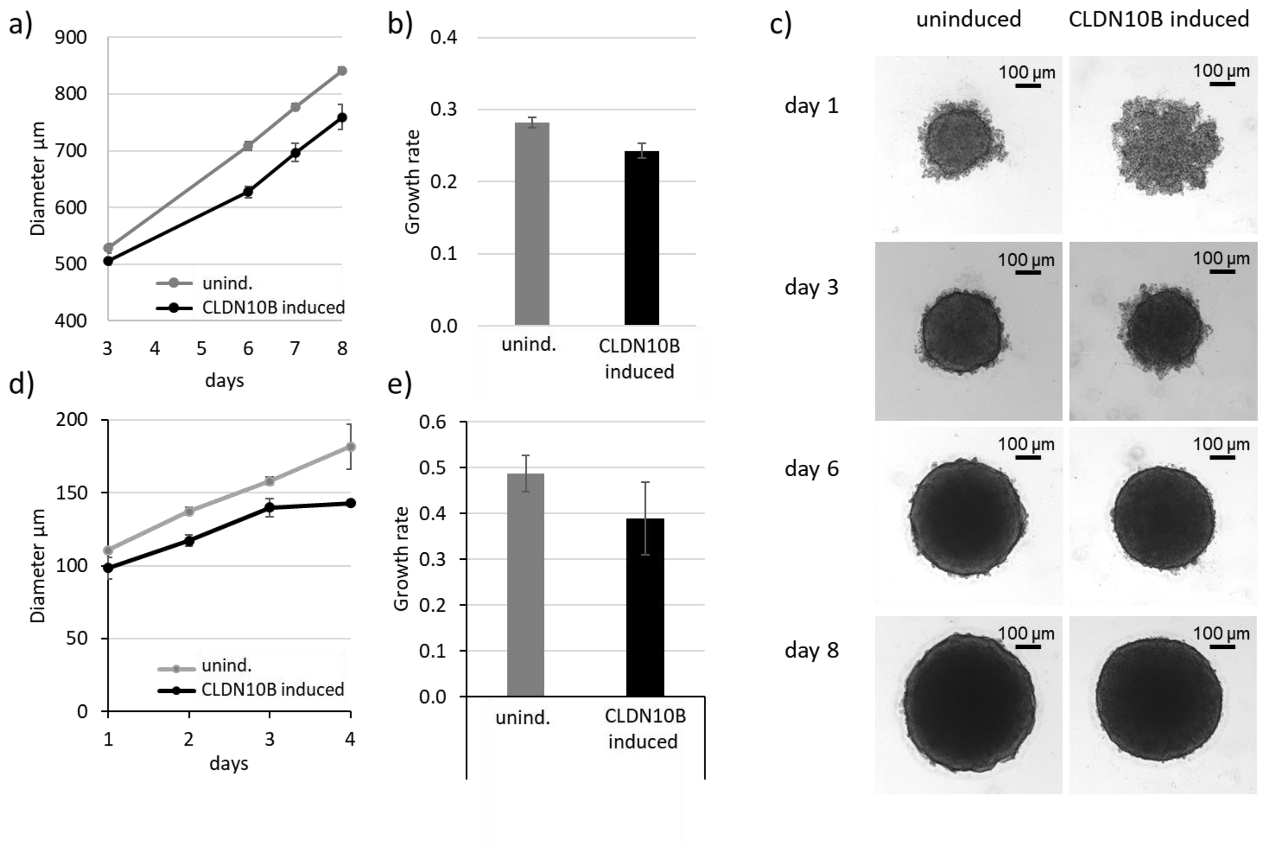

Suppl. Fig. 13. Overtime comparison of 3D static and dynamic cell culture in which CLDN10B reduces 3D spheroid growth of HEK Trex cell line. *Overtime 3D cell line growth was established for CLDN10B inducible cell line HEK Trex under static cell culture (a-c) and dynamic cell culture (d+e) conditions. Upon induction of CLDN10B by doxycycline the following parameters were determineed: diameter (a+d) and volume based growth rate (b+e). c) microscopy images of static spheroids of CLDN10B stable cell line uninduced and induced up to day 8.*

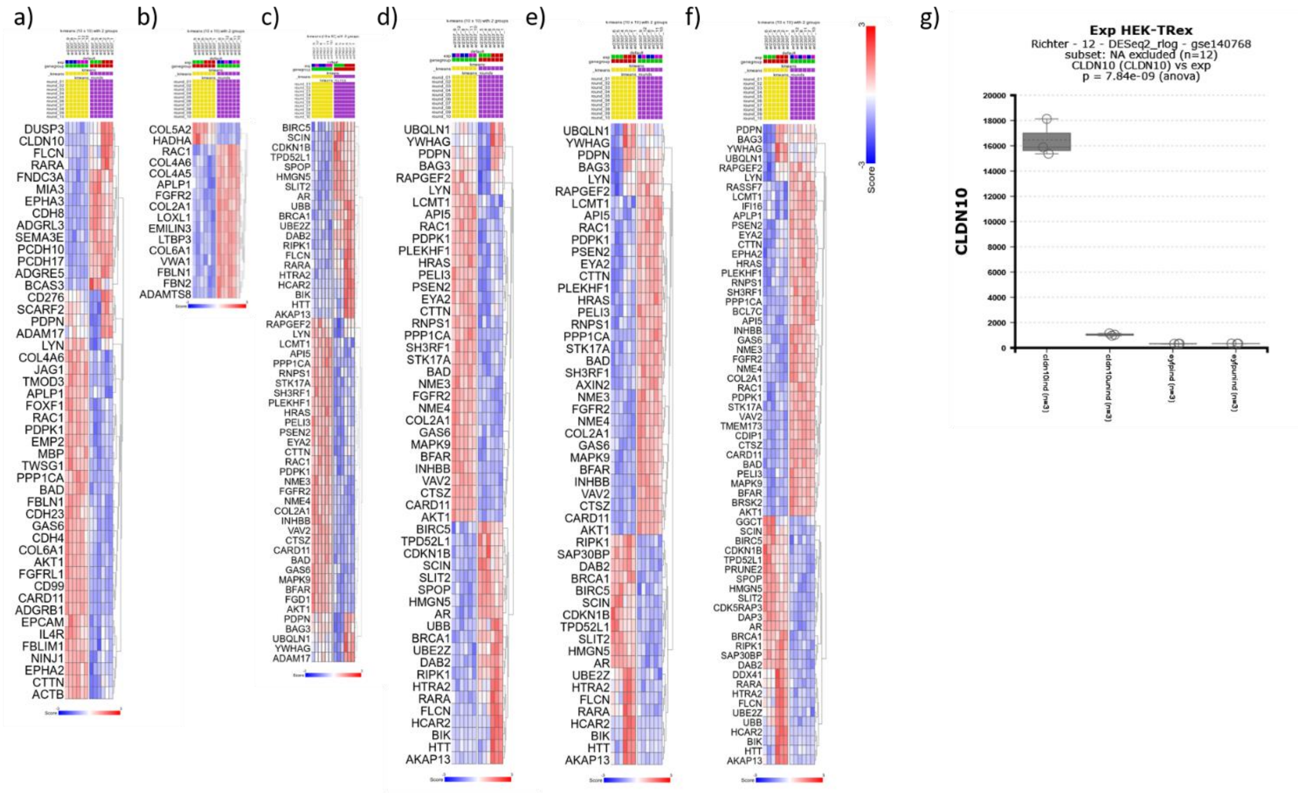

Suppl. Fig. 14. K-means clustering heatmap in CLDN10B induced 3D culture from Transcriptome analysis for several GO-term. *Differential expression between multiple groups was calculated with anova p<=0.01 and search by GO terms and k-means. a) GO 7155 cell adhesion n=64 meet criteria. b) GO 31012 extracellular matrix n=18 meet criteria. c) GO 42981 regulation of apoptotic process n=59 meet criteria. d) GO 43067 regulation of programmed cell death n=57 meet criteria. e) GO 10941 regulation of cell death n=58 meet criteria. f) GO 6915 apoptotic process n=71 meet criteria. g) Significant CLDN10 induction from array of the four conditions.*

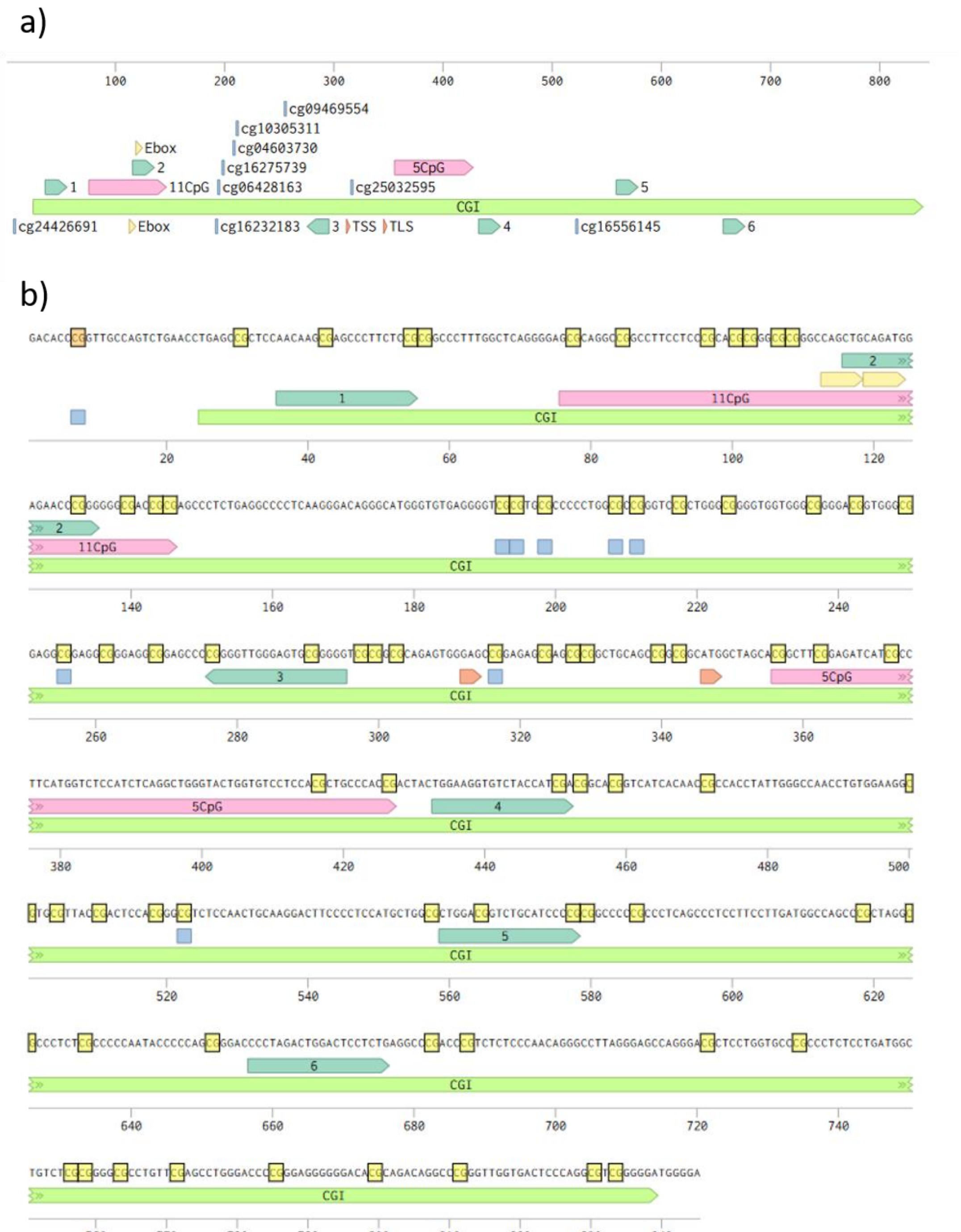

Suppl. Fig. 15. *CLDN10B promoter structure. CLDN10B promoter is shown with its CGI and CG enrichment (yellow boxes), methylation probes from array 450k and Epic (blue), guides for CRISPR targeting and epigenetic editing (green 1-6), Ebox motifs (yellow) and regions for pyrosequencing (pink, 11 CpGs upstream of TSS and 5 CpGs downstream of TSS). Generated with benchling. a) overview and b) detailed view.*

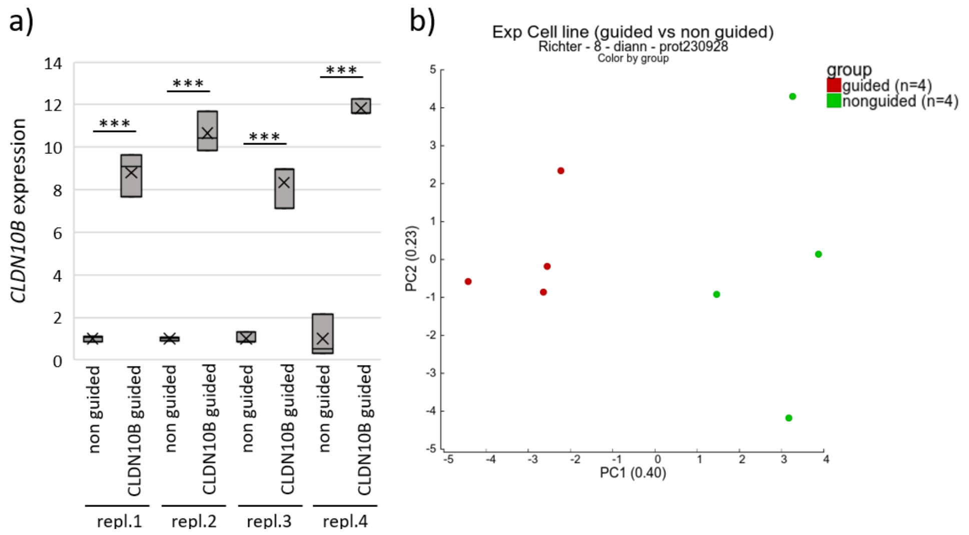

Suppl. Fig. 16. Whole proteome analysis upon CRISPR-based induction of CLND10 in HEK293T. *HEK293T cells were transfected with Cas9 under guidance with CLDN10B guide #2, cultured for three days and harvested. Experiment was performed in quadruplicates. a) CLDN10B expression was verified by RT-PCR. b) PCA analysis shows distinction between guided and non guided control conditions, with guided (CLDN10B, red) and non guided (green) by R2.*

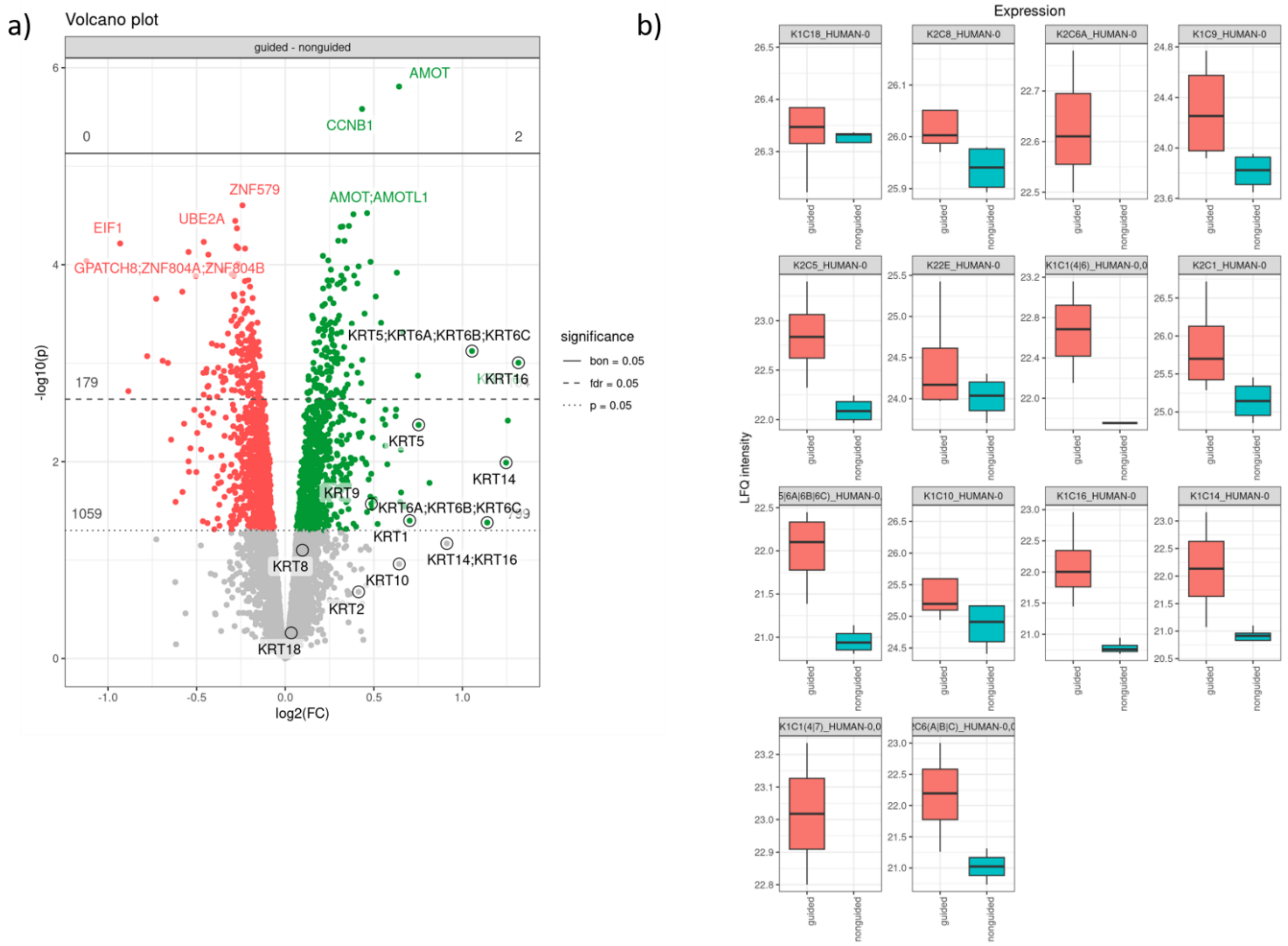

Suppl. Fig. 17. Whole proteome analysis upon CLDN10B induction. *HEK293T cells were transfected with Cas9 under guidance with CLDN10B guide #2, cultured for three days and harvested Experiment was performed in quadruplicates. a) Volcano plot with alterations of proteome and marked KRTs. b) Comparative changes of proteome upon CLDN10B induction as log2 fold change in relation to log10 p value shows KRTs are induced with b) single KRTs in groups guided (red) and non guided (green) as direct comparison.*

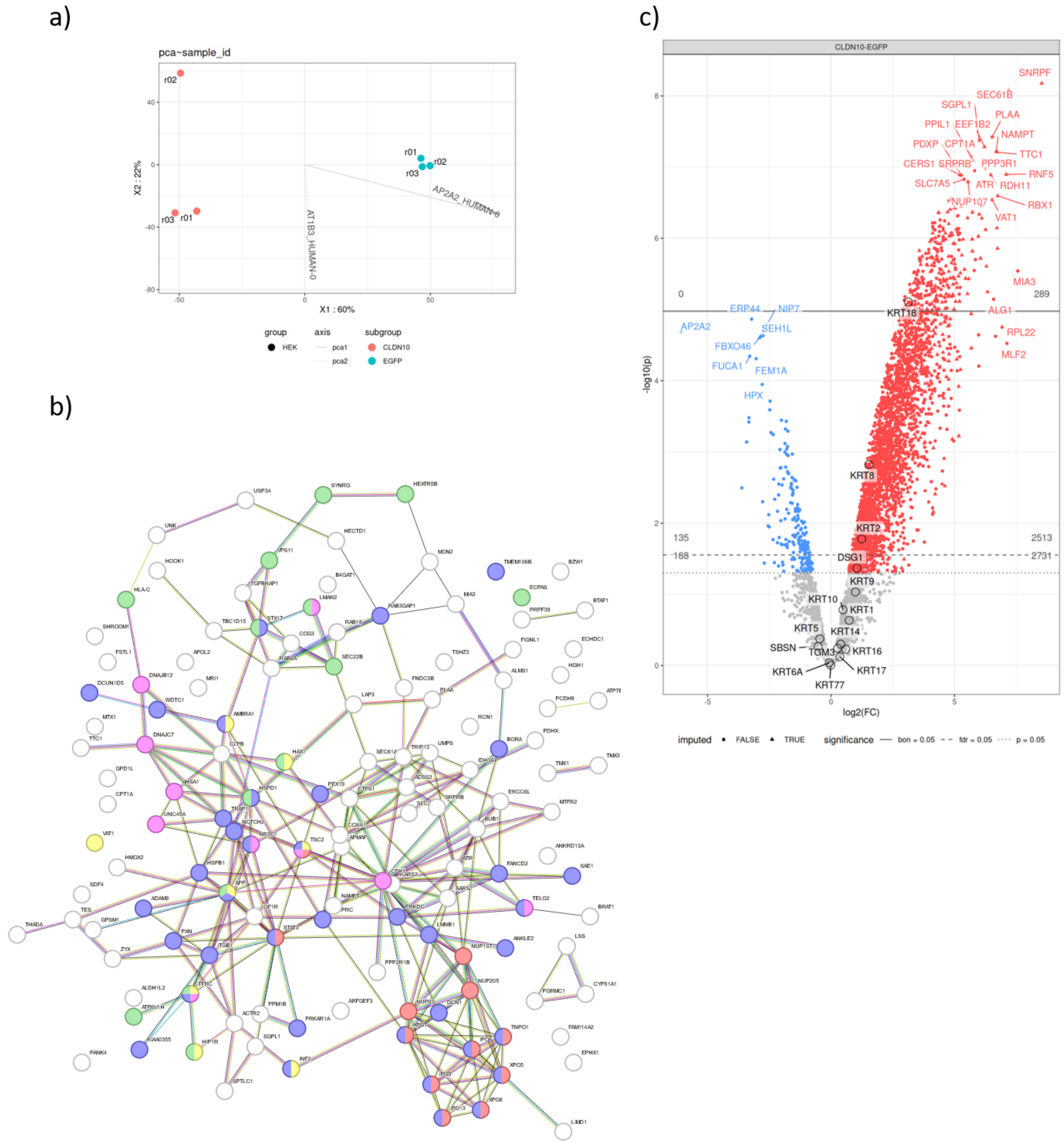

Suppl. Fig. 18. *CLDN10B interaction partner analysis by GFP-Trap. HEK293T cells were transfected with CLDN10B-GFP vs. GFP, cultured for three days, harvested and analyzed by mass spec. Experiment was performed in triplicates. CLDN10B expression was verified by fluorescence microscopy. a) PCA analysis shows distinction between groups of CLDN10B (red) and GFP only (green).b) String interaction network of CLDN10B binding partners with effect size >4 and with peptide count >5 in CLDN10B sample, number of nodes 135 with PPI enrichment p-value < 1.0e-16. Color code: Yellow Regulation of mitochondrion organization, Red Nucleocytoplasmic transport, Pink Heat shock protein binding, Blue Enzyme binding and Green coated vesicle. c) Volcano plot with CLDN10B binding partners. KRTs are not enriched as binding partners and are marked.*

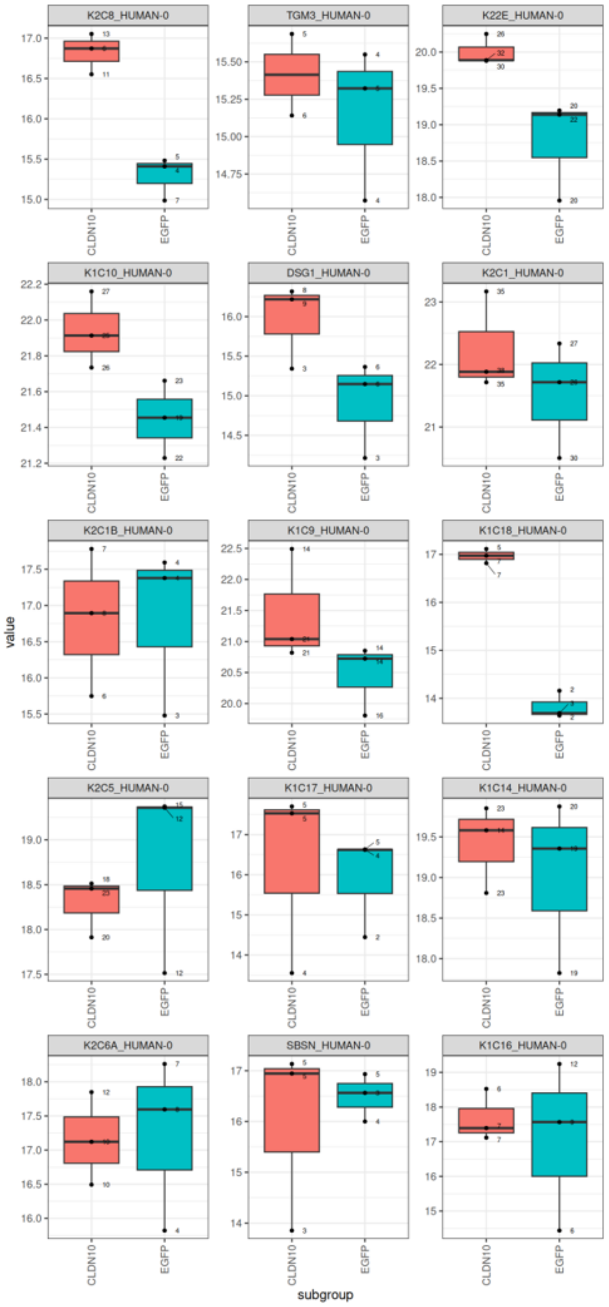

Suppl. Fig. 19. *CLDN10B interaction partner analysis by GFP-Trap. HEK293T cells were transfected with CLDN10B-GFP vs. GFP, cultured for three days, harvested and analyzed by mass spec. Experiment was performed in triplicates. CLDN10B expression was verified by fluorescence microscopy. Single candidate proteins from CLDN10B-GFP interaction (red) vs GFP only (blue) as direct comparison.*

a)

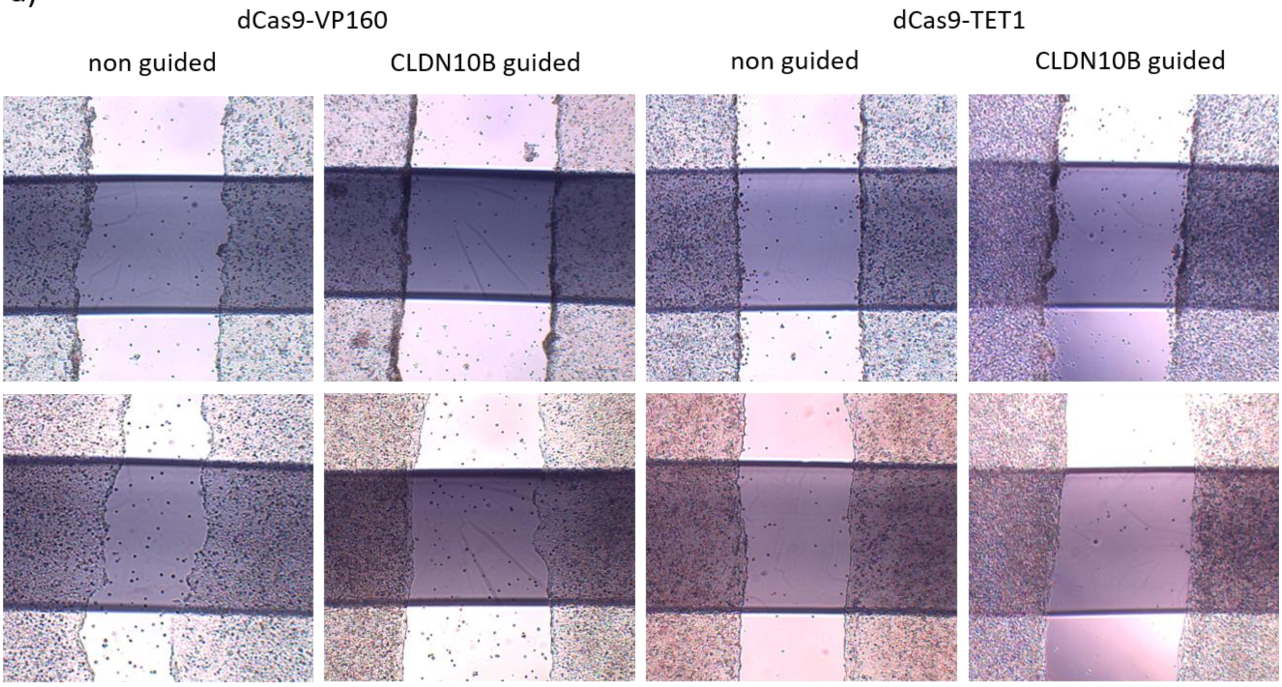

b)

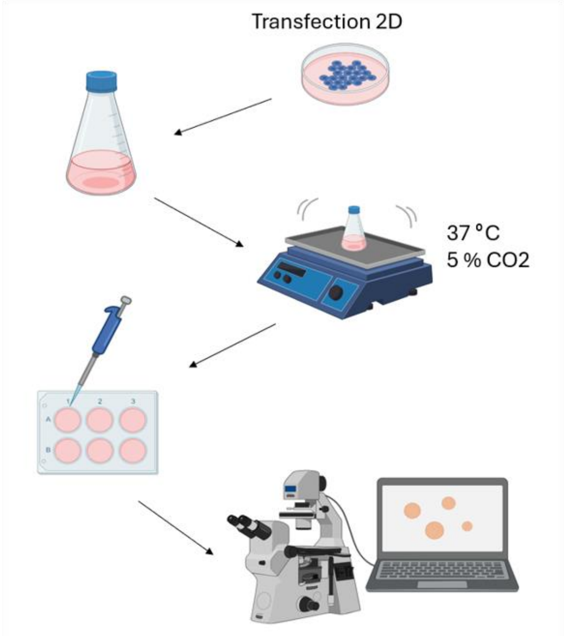

Suppl. Fig. 20. CLDN10B Epigenetic editing induction slows wound closure in 2D cell culture. *HEK cells were seeded at same cell number, transfected cells were selected by Puromycin (2,5 µg/ml) and grown to confluency in 2D cell culture, scratch was placed and position marked. Wound healing was analyzed on day 0 and after 48h at exact same position (n=27 for each condition) along scratch line. According quantification is shown in Fig. 6. b) Schematic overview of the 2D/3D cell culture procedure from Fig. 6. (created with biorender)*

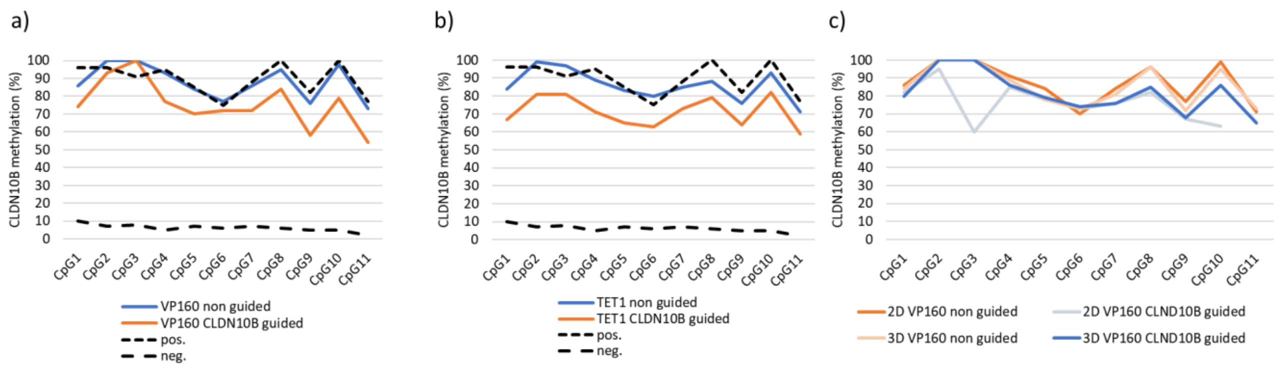

Suppl. Fig. 21. CLDN10B Epigenetic editing with promoter demethylation by Pyrosequencing by single CpG resolution. *For epigenetic editing of CLDN10B HEK cells were transfected with according CRISPR-dCas vecgors (either VP160 or TET1), DNA was isolated, BS treated and CLDN10B CGI pyrosequenced. Here single CpG resolution is shown (equivalent to Fig 6. b and e). CLDN10B was demethylated by a) VP160 and b) TET1 in pyrosequenced region of CGI (equivalent scratch data analysis) and c) CLDN10 demethylation by VP160 in 2D and 3D.*

Suppl. Table 1

Primer	Sequence 5'-3'	Analysis
Beta-Actin	CCTTCCTTCCTGGGCATGGAGTC	Expression
	CGGAGTACTTGCGCTCAGGAGGA	
GAPDH	TGGAGAAGGCTGGGGCTCAT	
	GACCTTGGCCAGGGGTGCTA	
CLDN10B	ACCGACTACTGGAAGGTGTC	
	GTATATAACCGTCCAGCGCCA	
CLDN10A	CTGCATGCCTAGACCACCAA	
	CAACGAGAGCTCCAAACCCT	
CLDN10B 5CpGs	TTTATTTTAGGTTGGGTATTGGTGTTT	CoBRA
	CCAACATAAAAAAAAAATCCTTACAATTAA	
	GATTATTGGAAGGTGTTTATTTA	Pyrosequencing
CLDN10B 11CpGs	GGTTGTTAGTTTGAATTTGAGTYGTTTTA	CoBRA
	ACCCCTCACACCCATACCCTAT	
	CCCTATCCCTTAAAAAACCTCAAAAAA	
	GGTTTTTTGGTTTAGGGGA	Pyrosequencing
CLDN10B #1	AACAAGCGAGCCCTTCTCCG	Epigenetic editing
CLDN10B #2	CTGCAGATGGAGAACCCGGG	
CLDN10B #3	CCCCCGCACTCCCAACCCCG	
CLDN10B #4	TGGAAGGTGTCTACCATCGA	
CLDN10B #5	CTGGACGGTCTGCATCCCCG	
CLDN10B #6	CCCTAGACTGGACTCCTCTG	
**Plasmid**	**Name**	**No.**
EGFP	pEGFP-C2 (clontech)	Addgene: 6083-1
TREx-system	pcDNA 4/TO/myc-His A (Invitrogen) insert: EGFP-only	Addgene: 2126<br>Cloned from EGFP-C2
	pcDNA 4/TO/myc-His A (Invitrogen) Insert: GFP-CLDN10B	Addgene: 2126; Cloned GFP-CLDN10B from pAcGFP1-N1_CLDN10b_wt
	pcDNA 4/TO/myc-His A (Invitrogen)	Addgene: 2126
SpCas9 (wt)	pSpCas9(BB)-2A-Puro (PX459)	Addgene: 48139
VP160	pAC154-dual-dCas9VP160-sgExpression	Addgene: 48240
TET1 (CD)	pdCas9-Tet1-CD	Addgene:158601
Guides (#)	pSpCas9(BB)-2A-Puro (PX459)<br>Cas9 deletion (Deutschmeyer et al. 2019)	Based on Addgene: 48139

Suppl. Table 2 first part

Name	HR	CI	P.value	adj.P.value	LR_test_pvalue	Best_split	CHR	MAPINFO	UCSC_RefGene_Name	UCSC_RefGene_Group	Relation_to_UCSC_CpG_Island
cg25311470	12.273	(3.891;38.713)	1.90E-05	0.0011	4.50E-11	q25	7	107950866	NRCAM	5'UTR	Open_Sea
cg18518804	10.326	(3.798;28.07)	4.70E-06	6.00E-04	2.10E-11	q25	14	91275103	TTC7B	Body	Open_Sea
cg21808448	9.562	(3.517;25.995)	9.70E-06	0.00084	1.60E-10	q25	1	39859609	MACF1	Body	Open_Sea
cg08210297	9.205	(3.388;25.011)	1.30E-05	0.00096	3.70E-10	q25	2	233320854	ALPI	1stExon;5'UTR	N_Shelf
cg16370701	8.838	(3.253;24.01)	1.90E-05	0.0011	9.70E-10	q25	6	43029051	KLC4	5'UTR;Body	S_Shore
cg16417118	8.78	(3.231;23.863)	2.10E-05	0.0011	1.20E-09	q25	1	2162931	SKI	Body	S_Shore
cg10876207	8.479	(3.121;23.035)	2.80E-05	0.0013	2.60E-09	q25	14	69726197	GALNTL1	TSS1500	N_Shore
cg19526600	8.36	(3.078;22.71)	3.10E-05	0.0014	3.50E-09	q25	1	54360115	DIO1	1stExon;Body	Open_Sea
cg01802898	8.236	(3.353;20.232)	4.30E-06	0.00057	1.60E-10	q25	1	16069621	TMEM82	Body	N_Shore
cg02382320	8.167	(3.322;20.073)	4.70E-06	6.00E-04	2.10E-10	q25	5	1090936	SLC12A7	Body	S_Shore
cg23154526	8.119	(2.989;22.056)	4.00E-05	0.0015	6.80E-09	q25	15	60297323	FOXB1	Body	Island
cg05906166	8.04	(3.263;19.808)	5.90E-06	0.00067	4.20E-10	q25	19	16437057	KLF2	Body	Island
cg27649239	7.944	(3.235;19.509)	6.20E-06	0.00068	3.90E-10	q25	15	68120393	LBXCOR1	Body	Island
cg05988605	7.892	(3.213;19.382)	6.60E-06	7.00E-04	4.60E-10	q25	8	28173887	PNOC	TSS1500	Open_Sea
cg27397850	7.604	(3.096;18.677)	9.60E-06	0.00084	1.20E-09	q25	7	27281941	EVX1	TSS1500	N_Shore
cg26959827	7.436	(3.024;18.286)	1.20E-05	0.00093	2.30E-09	q25	5	1335931	CLPTM1L	Body	S_Shore
cg22460204	7.428	(3.024;18.241)	1.20E-05	0.00092	2.00E-09	q25	7	39332836	POU6F2	Body	Open_Sea
cg09276998	7.381	(3.002;18.148)	1.30E-05	0.00095	2.70E-09	q25	10	29991884	SVIL	5'UTR	Open_Sea
cg18126247	7.371	(3.002;18.099)	1.30E-05	0.00095	2.40E-09	q25	5	176811239	SLC34A1	TSS200	Open_Sea
cg04023150	7.368	(3;18.095)	1.30E-05	0.00095	2.50E-09	q25	1	44873064	RNF220	5'UTR	Island
cg20421928	7.32	(2.978;17.995)	1.40E-05	0.00098	3.20E-09	q25	5	168419433	SLIT3	Body	Open_Sea
cg20741105	7.302	(2.972;17.941)	1.50E-05	0.00099	3.20E-09	q25	7	43686889	C7orf44	Body	Open_Sea
cg19056664	7.299	(2.97;17.935)	1.50E-05	0.00099	3.30E-09	q25	5	1335965	CLPTM1L	Body	S_Shore
cg13322131	7.286	(2.965;17.905)	1.50E-05	0.001	3.40E-09	q25	6	50814305	TFAP2B	3'UTR	S_Shore
cg14827391	7.263	(2.957;17.842)	1.50E-05	0.001	3.60E-09	q25	17	751287	NXN	Body	Open_Sea
cg11827925	7.258	(2.95;17.856)	1.60E-05	0.001	4.20E-09	q25	7	30951498	AQP1	1stExon;5'UTR	Open_Sea
cg27183801	7.231	(2.945;17.757)	1.60E-05	0.001	3.80E-09	q25	12	186358	IQSEC3	Body;TSS200	N_Shore
cg24830241	7.204	(2.933;17.694)	1.70E-05	0.001	4.20E-09	q25	7	101257675	MYL10	Body	Open_Sea
cg10308253	7.117	(2.898;17.475)	1.90E-05	0.0011	5.50E-09	q25	6	149803111	ZC3H12D	5'UTR	Open_Sea
cg16353006	7.051	(2.871;17.315)	2.00E-05	0.0011	6.80E-09	q25	1	197882469	LHX9	Body	S_Shore
cg08088222	7.049	(2.871;17.307)	2.00E-05	0.0011	6.80E-09	q25	12	122070432	ORAI1	Body	Open_Sea
cg02097120	7.037	(2.865;17.288)	2.10E-05	0.0012	7.40E-09	q25	1	154492952	TDRD10	Body	Open_Sea
cg09907542	7.037	(2.865;17.287)	2.10E-05	0.0012	7.40E-09	q25	4	7033761	TBC1D14	3'UTR	Open_Sea
cg08482307	7.023	(2.86;17.244)	2.10E-05	0.0012	7.30E-09	q25	5	14728684	ANKH	Body	Open_Sea
cg17278960	6.994	(2.848;17.174)	2.20E-05	0.0012	8.10E-09	q25	1	59775908	FGGY	5'UTR	Open_Sea
cg13364881	6.987	(2.845;17.162)	2.20E-05	0.0012	8.60E-09	q25	1	197880513	LHX9	TSS1500	Island
cg10569493	6.979	(2.842;17.136)	2.20E-05	0.0012	8.60E-09	q25	5	109200659	MAN2A1	Body	Open_Sea
cg18833228	6.975	(2.839;17.137)	2.30E-05	0.0012	9.20E-09	q25	19	735415	PALM	Body	N_Shore
cg17255063	6.973	(2.84;17.121)	2.30E-05	0.0012	8.70E-09	q25	4	41751830	PHOX2B	TSS1500	N_Shore
cg21821388	6.957	(2.831;17.092)	2.30E-05	0.0012	9.80E-09	q25	17	40352975	STAT5B	3'UTR	Open_Sea
cg22275125	6.957	(2.831;17.096)	2.40E-05	0.0012	1.00E-08	q25	11	62783573	SLC22A8	TSS1500	Open_Sea
cg13677149	6.949	(2.83;17.063)	2.30E-05	0.0012	9.50E-09	q25	7	27284789	EVX1	Body	Island
cg18182844	6.89	(3.021;15.713)	4.50E-06	0.00059	8.40E-10	q25	3	52828292	ITIH3	TSS1500	Open_Sea
cg14093715	6.842	(2.999;15.61)	4.90E-06	0.00061	1.00E-09	q25	9	126776180	LHX2	Body	Island
cg07719679	6.813	(2.774;16.736)	2.90E-05	0.0013	1.50E-08	q25	7	87936392	STEAP4	TSS200	Open_Sea
cg24051242	6.793	(2.765;16.691)	2.90E-05	0.0013	1.70E-08	q25	11	68096138	LRP5	Body	Open_Sea
cg18984724	6.765	(2.755;16.612)	3.00E-05	0.0014	1.70E-08	q25	2	220299604	SPEG	TSS200	Island
cg18281418	6.761	(2.753;16.602)	3.10E-05	0.0014	1.80E-08	q25	1	161193933	APOA2	TSS1500	Open_Sea
cg05437823	6.76	(2.753;16.599)	3.10E-05	0.0014	1.80E-08	q25	6	50813676	TFAP2B	3'UTR	Island
cg09236434	6.744	(2.746;16.559)	3.10E-05	0.0014	1.80E-08	q25	2	99553642	C2orf55	TSS1500	Island
cg06472439	6.701	(2.729;16.454)	3.30E-05	0.0014	2.10E-08	q25	5	92918560	NR2F1	TSS1500	N_Shore
cg24332577	6.701	(2.938;15.281)	6.10E-06	0.00068	1.70E-09	q25	20	50419248	SALL4	TSS1500	S_Shore
cg26380291	6.667	(2.922;15.213)	6.60E-06	7.00E-04	2.00E-09	q25	10	114787843	TCF7L2	Body	Open_Sea
cg08691577	6.662	(2.915;15.227)	6.90E-06	0.00071	2.40E-09	q25	5	14668489	FAM105B	Body	S_Shelf
cg16729555	6.648	(2.707;16.325)	3.60E-05	0.0015	2.50E-08	q25	1	207143533	FCAMR	5'UTR;1stExon	Open_Sea
cg04211179	6.642	(2.91;15.157)	6.90E-06	0.00071	2.30E-09	q25	1	16301562	ZBTB17	5'UTR	N_Shore
cg03218374	6.617	(2.695;16.25)	3.70E-05	0.0015	2.80E-08	q25	20	896981	ANGPT4	TSS200	Open_Sea
cg06501790	6.611	(2.898;15.082)	7.20E-06	0.00073	2.50E-09	q25	5	176811257	SLC34A1	TSS200	Open_Sea
cg14121142	6.592	(2.885;15.062)	7.70E-06	0.00075	3.10E-09	q25	12	49391363	DDN	Body	Island
cg04223420	6.551	(2.871;14.948)	8.00E-06	0.00076	3.10E-09	q25	5	134363877	PITX1	3'UTR	Island
cg00091569	6.525	(2.657;16.022)	4.30E-05	0.0016	3.80E-08	q25	3	40428383	ENTPD3	TSS1500	N_Shore
cg04961911	6.525	(2.858;14.899)	8.50E-06	0.00079	3.70E-09	q25	9	35079141	FANCG	Body	N_Shore
cg03846076	6.502	(2.648;15.969)	4.40E-05	0.0016	4.10E-08	q25	5	172743837	STC2	3'UTR	Open_Sea
cg26125625	6.469	(2.635;15.882)	4.60E-05	0.0016	4.50E-08	q25	3	124860871	SLC12A8	Body	Island
cg21550612	6.417	(2.81;14.654)	1.00E-05	0.00086	5.60E-09	q25	6	31094185	PSORS1C1	5'UTR	Open_Sea
cg11419403	6.375	(2.794;14.549)	1.10E-05	0.00087	6.10E-09	q25	5	1335846	CLPTM1L	Body	S_Shore
cg09906647	6.367	(2.793;14.518)	1.10E-05	0.00087	5.70E-09	q25	17	26939344	FLJ25006;LOC645851	Body;TSS1500	Open_Sea
cg25792439	6.364	(2.79;14.513)	1.10E-05	0.00087	5.90E-09	q25	17	78163268	CARD14	Body	N_Shore
cg02174203	6.354	(2.785;14.495)	1.10E-05	0.00088	6.40E-09	q25	11	76848054	MYO7A	Body	N_Shore
cg22125838	6.352	(2.586;15.603)	5.50E-05	0.0018	7.00E-08	q25	2	219757961	WNT10A	Body	Island
cg14327393	6.346	(2.78;14.483)	1.10E-05	0.00089	6.90E-09	q25	9	75141633	TMC1	5'UTR	Open_Sea
cg13430450	6.345	(2.778;14.494)	1.20E-05	9.00E-04	7.50E-09	q25	9	107691064	ABCA1	TSS1500	S_Shore
cg15129144	6.315	(2.766;14.419)	1.20E-05	0.00092	8.10E-09	q25	2	46527958	EPAS1	Body	S_Shore
cg16402814	6.312	(2.57;15.503)	5.80E-05	0.0018	7.80E-08	q25	11	915227	CHID1	TSS200	S_Shelf
cg16129200	6.256	(2.743;14.267)	1.30E-05	0.00095	9.00E-09	q25	8	98879893	MATN2	TSS1500	N_Shore
cg18606375	6.244	(2.738;14.237)	1.30E-05	0.00095	9.20E-09	q25	5	134365728	PITX1	Body	N_Shore
cg07063883	6.243	(2.542;15.331)	6.50E-05	0.0019	9.70E-08	q25	16	3079953	CCDC64B	Body	Island
cg16606561	6.242	(2.737;14.236)	1.30E-05	0.00096	9.50E-09	q25	20	824641	FAM110A	5'UTR;TSS1500	N_Shore
cg05007126	6.216	(2.724;14.184)	1.40E-05	0.00098	1.10E-08	q25	20	1310884	SDCBP2	TSS1500	Open_Sea
cg21898844	6.216	(2.723;14.192)	1.40E-05	0.00098	1.20E-08	q25	14	89628169	FOXN3	3'UTR	N_Shore
cg00334863	6.192	(2.712;14.138)	1.50E-05	0.001	1.30E-08	q25	22	42779093	NFAM1	3'UTR	Open_Sea
cg21827581	6.192	(2.716;14.119)	1.50E-05	0.00099	1.10E-08	q25	2	3328497	TSSC1	Body	Open_Sea
cg03080147	6.144	(2.695;14.01)	1.60E-05	0.001	1.30E-08	q25	8	17270347	MTMR7	Body	N_Shore
cg16009558	6.133	(2.689;13.988)	1.60E-05	0.001	1.40E-08	q25	6	101846707	GRIK2	TSS200	N_Shore
cg12284382	6.132	(2.687;13.991)	1.60E-05	0.001	1.50E-08	q25	22	35940438	RASD2	5'UTR	S_Shelf
cg06639440	6.121	(2.492;15.034)	7.80E-05	0.002	1.50E-07	q25	11	915170	CHID1	TSS200	S_Shelf
cg00667298	6.093	(2.82;13.165)	4.30E-06	0.00058	2.70E-09	q25	6	29576329	GABBR1	Body	Open_Sea
cg00085732	6.089	(2.67;13.887)	1.70E-05	0.0011	1.70E-08	q25	17	26800820	SLC13A2	1stExon;Body	Open_Sea
cg07426848	6.087	(2.669;13.882)	1.80E-05	0.0011	1.70E-08	q25	1	153521712	S100A3	5'UTR;1stExon	Open_Sea
cg25446086	6.081	(2.665;13.875)	1.80E-05	0.0011	1.80E-08	q25	1	155829549	SYT11	1stExon;5'UTR	N_Shore
cg05958050	6.065	(2.658;13.835)	1.80E-05	0.0011	1.90E-08	q25	16	56703400	MT1G;MT1H	TSS1500	Island
cg00812578	6.043	(2.798;13.052)	4.70E-06	6.00E-04	3.30E-09	q25	6	31625701	APOM	Body	N_Shelf
cg08960448	6.037	(2.798;13.028)	4.60E-06	6.00E-04	3.10E-09	q25	16	4838658	SEPT12;LOC440335	TSS1500;Body	Open_Sea
cg04372674	6.01	(2.633;13.722)	2.10E-05	0.0011	2.50E-08	q25	7	30951381	AQP1	TSS200	Open_Sea
cg25479916	6	(2.443;14.738)	9.30E-05	0.0022	2.20E-07	q25	6	27841392	HIST1H4L;HIST1H3I	TSS200;TSS1500	S_Shore
cg03882698	5.965	(2.614;13.613)	2.20E-05	0.0012	2.80E-08	q25	3	69230955	FRMD4B	Body	Open_Sea
cg06271561	5.961	(2.611;13.613)	2.30E-05	0.0012	3.00E-08	q25	15	85359854	ALPK3	TSS200	N_Shore
cg09174162	5.948	(2.608;13.567)	2.30E-05	0.0012	2.90E-08	q25	7	2150845	MAD1L1	Body	N_Shelf
cg13465946	5.948	(2.608;13.567)	2.20E-05	0.0012	2.90E-08	q25	19	13208349	NFIX	3'UTR	Island
cg13799919	5.935	(2.602;13.54)	2.30E-05	0.0012	3.10E-08	q25	13	28212848	POLR1D	Body	Open_Sea
cg04205653	0.114	(0.042;0.31)	2.10E-05	0.0011	1.20E-09	q75	21	46225890	SUMO3	3'UTR	S_Shelf
cg09775238	0.138	(0.056;0.339)	1.60E-05	0.001	3.90E-09	q75	16	86580410	MTHFSD	Body	Open_Sea
cg07281781	0.14	(0.061;0.321)	3.20E-06	0.00051	4.70E-10	q75	2	242794854	PDCD1	Body	Island
cg17564498	0.146	(0.064;0.334)	5.20E-06	0.00063	1.40E-09	q75	7	133247477	EXOC4	Body	Open_Sea
cg23620049	0.157	(0.069;0.359)	1.10E-05	0.00089	6.90E-09	q75	3	185270558	LIPH	TSS200	Open_Sea
cg08907436	0.159	(0.07;0.363)	1.30E-05	0.00094	9.30E-09	q75	6	152125965	ESR1	5'UTR;TSS1500	N_Shelf
cg17051905	0.159	(0.07;0.362)	1.20E-05	0.00091	7.30E-09	q75	8	1725140	CLN8	Body	Open_Sea
cg03151191	0.16	(0.07;0.366)	1.40E-05	0.00098	1.20E-08	q75	7	98600622	TRRAP	Body	Open_Sea
cg03339077	0.161	(0.07;0.367)	1.50E-05	0.00099	1.20E-08	q75	11	47165057	C11orf49	Body	Open_Sea
cg08455936	0.168	(0.078;0.362)	5.40E-06	0.00064	4.30E-09	q75	16	27781069	KIAA0556	Body	Open_Sea
cg24867601	0.168	(0.074;0.383)	2.20E-05	0.0012	2.80E-08	q75	7	104758865	SRPK2	Body	Open_Sea
cg14669515	0.169	(0.074;0.385)	2.40E-05	0.0012	3.20E-08	q75	1	9386821	SPSB1	5'UTR	Open_Sea
cg23432795	0.173	(0.086;0.347)	8.50E-07	0.00027	7.80E-10	q75	7	99036699	CPSF4;PTCD1	5'UTR;TSS1500;1stExon	Island
cg10789050	0.175	(0.081;0.378)	8.70E-06	8.00E-04	1.10E-08	q75	3	31713847	OSBPL10	Body	Open_Sea
cg00164583	0.176	(0.077;0.402)	3.70E-05	0.0015	8.20E-08	q75	16	88554420	ZFPM1	Body	Island
cg18382893	0.176	(0.081;0.379)	9.50E-06	0.00083	1.30E-08	q75	6	30139641	TRIM15	Body	N_Shore
cg26103306	0.176	(0.081;0.381)	1.00E-05	0.00086	1.70E-08	q75	4	154515617	KIAA0922	Body	Open_Sea
cg16517298	0.177	(0.082;0.381)	9.80E-06	0.00084	1.40E-08	q75	1	230413174	GALNT2	Body	N_Shelf
cg17940234	0.177	(0.082;0.383)	1.10E-05	0.00087	1.70E-08	q75	6	150070697	PCMT1	TSS200	Island
cg00762029	0.179	(0.083;0.387)	1.20E-05	0.00092	2.30E-08	q75	4	185317438	IRF2	Body	Open_Sea
cg02478828	0.179	(0.083;0.388)	1.30E-05	0.00093	2.40E-08	q75	2	74153195	DGUOK	TSS1500	N_Shore
cg07375700	0.179	(0.083;0.387)	1.20E-05	9.00E-04	1.90E-08	q75	1	2013507	PRKCZ	Body;5'UTR	Open_Sea
cg08151370	0.18	(0.088;0.372)	3.50E-06	0.00052	4.20E-09	q75	3	43073152	FAM198A	Body	Open_Sea
cg14606328	0.18	(0.084;0.388)	1.20E-05	0.00092	2.10E-08	q75	21	45177639	PDXK	3'UTR	Island

Suppl. Table 2 second part

cg18809076	0.18	(0.079;0.41)	4.50E-05	0.0016	1.30E-07	q75	7	55177623	EGFR	Body	Open_Sea
cg26683023	0.183	(0.089;0.377)	4.30E-06	0.00057	6.50E-09	q75	1	227750892	ZNF678	TSS1500	N_Shore
cg00347863	0.184	(0.089;0.378)	4.30E-06	0.00058	6.20E-09	q75	7	94220697	SGCE	Body	Open_Sea
cg21217846	0.184	(0.085;0.397)	1.60E-05	0.001	3.70E-08	q75	1	153515539	S100A5	TSS1500	Open_Sea
cg27245185	0.185	(0.086;0.399)	1.70E-05	0.001	4.00E-08	q75	12	99257038	ANKS1B	Body	Open_Sea
cg05272790	0.188	(0.087;0.406)	2.10E-05	0.0011	6.20E-08	q75	11	117800276	TMPRSS13	TSS200	Open_Sea
cg16174225	0.19	(0.088;0.409)	2.20E-05	0.0012	6.80E-08	q75	1	1268469	TAS1R3	Body	Island
cg06592860	0.192	(0.089;0.414)	2.60E-05	0.0013	9.60E-08	q75	16	88969627	CBFA2T3	5'UTR;Body	N_Shore
cg21923525	0.192	(0.096;0.382)	2.70E-06	0.00047	6.00E-09	q75	18	9474143	RALBP1	TSS1500	N_Shore
cg09890339	0.193	(0.093;0.397)	8.10E-06	0.00077	2.10E-08	q75	12	2734150	CACNA1C	Body	Open_Sea
cg15366555	0.194	(0.09;0.418)	2.90E-05	0.0013	1.20E-07	q75	2	74601926	DCTN1	Body;TSS200	Open_Sea
cg14387452	0.195	(0.09;0.42)	3.00E-05	0.0013	1.20E-07	q75	9	20512334	MLLT3	Body	Open_Sea
cg12691427	0.196	(0.095;0.404)	1.00E-05	0.00086	3.20E-08	q75	8	141567025	EIF2C2	Body	Open_Sea
cg21553980	0.196	(0.091;0.422)	3.20E-05	0.0014	1.40E-07	q75	17	109444	RPH3AL	Body	Open_Sea
cg03861143	0.197	(0.091;0.425)	3.40E-05	0.0014	1.50E-07	q75	11	1855746	SYT8	1stExon;5'UTR	Open_Sea
cg05441854	0.197	(0.096;0.405)	1.00E-05	0.00086	3.30E-08	q75	16	85650374	KIAA0182	5'UTR;Body	S_Shore
cg00731304	0.199	(0.092;0.429)	3.90E-05	0.0015	2.10E-07	q75	6	18458468	RNF144B	Body	Open_Sea
cg11834658	0.199	(0.093;0.43)	3.80E-05	0.0015	1.90E-07	q75	1	21611224	ECE1	Body	Open_Sea
cg08080213	0.2	(0.097;0.411)	1.20E-05	0.00093	4.50E-08	q75	19	55866907	FAM71E2;COX6B2	Body;TSS1500	S_Shore
cg27565899	0.2	(0.097;0.411)	1.30E-05	0.00093	4.80E-08	q75	1	110166664	AMPD2	Body;TSS1500	S_Shelf
cg25296314	0.202	(0.098;0.416)	1.40E-05	0.00098	5.90E-08	q75	12	122277851	HPD	Body	S_Shore
cg04676766	0.203	(0.099;0.419)	1.60E-05	0.001	7.00E-08	q75	4	153699460	TIGD4	5'UTR	N_Shore
cg03466598	0.204	(0.095;0.44)	5.00E-05	0.0017	3.20E-07	q75	16	1305718	TPSD1	TSS1500	N_Shelf
cg13441891	0.204	(0.099;0.42)	1.60E-05	0.001	7.20E-08	q75	16	85648370	KIAA0182	5'UTR;Body	Island
cg07631359	0.205	(0.095;0.441)	5.00E-05	0.0017	3.20E-07	q75	3	10276672	IRAK2	Body	S_Shore
cg09245698	0.205	(0.103;0.408)	6.50E-06	7.00E-04	2.80E-08	q75	6	159084370	SYTL3	Body	Open_Sea
cg24527751	0.205	(0.095;0.441)	5.10E-05	0.0017	3.20E-07	q75	2	171877888	TLK1	Body	Open_Sea
cg00377332	0.206	(0.1;0.425)	1.90E-05	0.0011	9.70E-08	q75	3	45761250	SACM1L	Body	Open_Sea
cg02160684	0.206	(0.1;0.425)	1.80E-05	0.0011	9.30E-08	q75	8	126448033	TRIB1	Body	S_Shelf
cg19558628	0.206	(0.1;0.425)	1.80E-05	0.0011	9.40E-08	q75	14	24801616	ADCY4	Body	N_Shore
cg23337031	0.206	(0.104;0.409)	6.30E-06	0.00069	2.60E-08	q75	2	236416064	AGAP1	Body	Open_Sea
cg24870967	0.206	(0.096;0.444)	5.50E-05	0.0017	3.70E-07	q75	5	115783345	SEMA6A	Body	Island
cg10607603	0.207	(0.101;0.427)	2.00E-05	0.0011	1.10E-07	q75	17	73151410	HN1	TSS1500	S_Shore
cg11964810	0.207	(0.096;0.445)	5.60E-05	0.0018	3.90E-07	q75	1	1478170	SSU72	Body	S_Shore
cg04026948	0.208	(0.101;0.429)	2.20E-05	0.0012	1.40E-07	q75	8	97953330	PGCP	Body	Open_Sea
cg18122696	0.208	(0.096;0.447)	5.90E-05	0.0018	4.20E-07	q75	11	1855389	SYT8	TSS1500	Open_Sea
cg20251110	0.208	(0.101;0.43)	2.10E-05	0.0012	1.30E-07	q75	12	124813301	NCOR2	Body	S_Shelf
cg14071947	0.209	(0.097;0.45)	6.40E-05	0.0019	5.10E-07	q75	9	136229324	SURF4	3'UTR	Open_Sea
cg23638518	0.209	(0.102;0.43)	2.10E-05	0.0012	1.20E-07	q75	6	39080067	C6orf64	Body	N_Shelf
cg16255307	0.21	(0.102;0.432)	2.30E-05	0.0012	1.50E-07	q75	3	185271383	LIPH	TSS1500	Open_Sea
cg16266918	0.21	(0.102;0.433)	2.40E-05	0.0012	1.50E-07	q75	21	45175448	PDXK	Body	N_Shelf
cg08009697	0.211	(0.106;0.419)	8.70E-06	8.00E-04	4.40E-08	q75	7	104883450	SRPK2	Body	N_Shore
cg13475704	0.212	(0.103;0.437)	2.60E-05	0.0013	1.80E-07	q75	17	77810269	CBX4	Body	N_Shore
cg18984002	0.212	(0.103;0.437)	2.60E-05	0.0013	1.80E-07	q75	17	36876514	MLLT6	Body	Open_Sea
cg04438064	0.213	(0.104;0.439)	2.80E-05	0.0013	1.90E-07	q75	2	240161619	HDAC4	Body	Open_Sea
cg05722035	0.213	(0.107;0.425)	1.10E-05	0.00089	7.20E-08	q75	3	56896467	ARHGEF3	Body	Open_Sea
cg07351675	0.213	(0.099;0.458)	7.70E-05	0.002	7.10E-07	q75	7	1578263	MAFK	5'UTR	S_Shelf
cg11154879	0.213	(0.103;0.439)	2.80E-05	0.0013	2.00E-07	q75	20	61001775	C20orf151	5'UTR	Open_Sea
cg14682345	0.213	(0.107;0.425)	1.10E-05	0.00089	7.60E-08	q75	19	1104646	GPX4	Body;TSS200	Island
cg17774001	0.213	(0.099;0.459)	8.00E-05	0.0021	7.80E-07	q75	2	25600752	DTNB	3'UTR	S_Shore
cg01147727	0.215	(0.1;0.463)	8.50E-05	0.0021	8.60E-07	q75	14	105363015	KIAA0284	3'UTR	S_Shelf
cg11274172	0.215	(0.104;0.442)	3.00E-05	0.0014	2.40E-07	q75	11	75736215	UVRAG	Body	Open_Sea
cg18089519	0.215	(0.1;0.463)	8.50E-05	0.0021	8.80E-07	q75	11	763339	TALDO1	Body	Open_Sea
cg19360930	0.215	(0.1;0.464)	8.80E-05	0.0022	9.20E-07	q75	2	20111778	WDR35	3'UTR	Open_Sea
cg08522784	0.216	(0.105;0.445)	3.30E-05	0.0014	2.70E-07	q75	6	38060312	ZFAND3	Body	Open_Sea
cg15612947	0.216	(0.109;0.429)	1.20E-05	0.00091	7.70E-08	q75	5	14464064	TRIO	Body	S_Shelf
cg19752861	0.216	(0.105;0.445)	3.20E-05	0.0014	2.60E-07	q75	2	74153213	DGUOK	TSS1500	N_Shore
cg23657388	0.216	(0.105;0.446)	3.30E-05	0.0014	2.80E-07	q75	7	101171330	EMID2	Body	Open_Sea
cg04468741	0.217	(0.109;0.43)	1.30E-05	0.00093	8.60E-08	q75	11	12181467	MICAL2	5'UTR	Open_Sea
cg07011711	0.217	(0.105;0.447)	3.50E-05	0.0014	3.10E-07	q75	11	61467286	DAGLA	5'UTR	Open_Sea
cg07618085	0.217	(0.105;0.447)	3.40E-05	0.0014	2.80E-07	q75	17	77924582	TBC1D16	Body	S_Shore
cg07855083	0.217	(0.106;0.448)	3.50E-05	0.0014	3.10E-07	q75	2	200320871	SATB2	5'UTR	Island
cg08925882	0.217	(0.109;0.431)	1.30E-05	0.00093	8.50E-08	q75	11	67350491	GSTP1	TSS1500	N_Shore
cg10603826	0.217	(0.101;0.467)	9.30E-05	0.0022	1.00E-06	q75	12	26986825	ITPR2	TSS1500	S_Shore
cg23549264	0.217	(0.122;0.387)	2.20E-07	0.00016	1.10E-09	mean	13	21714632	SAP18	TSS200	Island
cg23696432	0.217	(0.105;0.447)	3.40E-05	0.0014	3.00E-07	q75	19	45150725	PVR	Body	Island
cg00167684	0.218	(0.106;0.448)	3.50E-05	0.0014	3.00E-07	q75	16	70505196	FUK	Body	N_Shore
cg00705992	0.218	(0.11;0.432)	1.30E-05	0.00094	8.90E-08	q75	7	27226329	HOXA11AS;HOXA11	Body;TSS1500	N_Shore
cg26799474	0.219	(0.111;0.435)	1.40E-05	0.00098	1.00E-07	q75	2	202098951	CASP8	5'UTR	Open_Sea
cg06720949	0.221	(0.107;0.455)	4.20E-05	0.0016	4.10E-07	q75	19	45381937	PVRL2	Body;3'UTR	Open_Sea
cg21986821	0.221	(0.108;0.456)	4.20E-05	0.0016	4.20E-07	q75	19	6730061	GPR108	3'UTR	Open_Sea
cg02852421	0.222	(0.107;0.458)	4.70E-05	0.0016	5.30E-07	q75	15	70372614	TLE3;MIR629	Body;TSS1500	Open_Sea
cg07925633	0.222	(0.112;0.442)	1.80E-05	0.0011	1.60E-07	q75	17	30469120	RHOT1	TSS1500	N_Shore
cg20018344	0.222	(0.112;0.441)	1.70E-05	0.0011	1.40E-07	q75	20	3204138	ITPA	3'UTR	Open_Sea
cg04205769	0.223	(0.108;0.459)	4.60E-05	0.0016	5.00E-07	q75	1	101488098	DPH5	Body	N_Shelf
cg08470264	0.223	(0.108;0.458)	4.60E-05	0.0016	4.90E-07	q75	16	86580384	MTHFSD	Body	Open_Sea

Top biomarker of ccRCC in KIRC: Analysed by Methsurv

Suppl. Table 3 first part

gene	protein	uniprot	effect~CLDN10-EGFP~limma	t~CLDN10-EGFP~limma	p~CLDN10-EGFP~limma	p~F~limma	effect~CLDN10-EGFP~limma	t~CLDN10-EGFP~limma	p-log10~CLDN10-EGFP~limma	p-log10~F~limma	maxlfq_HEK_EGFP_r01	maxlfq_HEK_EGFP_r02	maxlfq_HEK_EGFP_r03	maxlfq_HEK_CLDN10_r01	maxlfq_HEK_CLDN10_r02	maxlfq_HEK_CLDN10_r03	n_pep_HEK_EGFP_r01	n_pep_HEK_EGFP_r02	n_pep_HEK_EGFP_r03	n_pep_HEK_CLDN10_r01	n_pep_HEK_CLDN10_r02	n_pep_HEK_CLDN10_r03
MIA3	TGO1_HUMAN	Q5JRA6	7.57	1.59E+01	3.24E-06	3.24E-06	-8.79E-01	-1.20E+00	5.49E+00	5.49E+00	12.34			20.01	19.96	19.73	2	1	1	21	10	20
NAMPT	NAMPT_HUMAN	P43490	6.69	1.82E+01	7.19E-08	7.19E-08	-8.25E-01	-1.26E+00	7.14E+00	7.14E+00	14.52	14.95	14.91	21.63	20.90	21.92	2	4	3	23	18	23
PLAA	PLAP_HUMAN	Q9Y263	6.53	1.94E+01	4.29E-08	4.29E-08	-8.15E-01	-1.29E+00	7.37E+00	7.37E+00	12.84	13.66	13.37	19.88	19.65	19.92	3	4	3	29	23	30
ATR	ATR_HUMAN	Q13535	6.21	1.61E+01	1.90E-07	1.90E-07	-7.93E-01	-1.21E+00	6.72E+00	6.72E+00	12.80	13.34	13.71	19.79	18.97	19.72	2	4	3	21	7	23
SGPL1	SGPL1_HUMAN	O95470	6.00	1.93E+01	4.55E-08	4.55E-08	-7.78E-01	-1.28E+00	7.34E+00	7.34E+00	13.82	14.26	13.76	19.80	19.92	20.10	4	4	4	7	6	7
GATD3;GA	GAL3(A|B)_HUMAN	A0A0B4J2D	5.99	7.33E+00	7.59E-05	7.59E-05	-7.77E-01	-8.65E-01	4.12E+00	4.12E+00	16.71	13.03	14.37	20.70	21.02	20.35	3	3	4	9	7	8
PEX19	PEX19_HUMAN	P40855	5.98	9.45E+00	2.78E-05	2.78E-05	-7.76E-01	-9.76E-01	4.56E+00	4.56E+00		11.89	11.25	18.43	17.82	16.38	1	2	2	8	6	8
SRPRB	SRPRB_HUMAN	Q9Y5M8	5.81	1.70E+01	1.26E-07	1.26E-07	-7.64E-01	-1.23E+00	6.90E+00	6.90E+00	15.09	15.80	15.37	21.28	21.50	20.90	5	7	7	13	11	13
SEC22B	SC22B_HUMAN	O75396	5.80	1.11E+01	3.46E-06	3.46E-06	-7.63E-01	-1.04E+00	5.46E+00	5.46E+00	13.22	14.12	13.69	19.19	20.57	18.66	3	3	3	8	10	8
HAX1	HAX1_HUMAN	O00165	5.23	1.20E+01	5.55E-06	5.55E-06	-7.18E-01	-1.08E+00	5.26E+00	5.26E+00		15.22	15.62	20.62	20.13	21.19	1	5	3	12	7	12
UNC45A	UN45A_HUMAN	Q9H3U1	5.21	1.31E+01	9.76E-07	9.76E-07	-7.17E-01	-1.12E+00	6.01E+00	6.01E+00	15.25	14.82	14.35	19.69	20.62	19.74	8	9	7	37	35	39
HSPB1	HSPB1_HUMAN	P04792	5.21	7.66E+00	5.50E-05	5.50E-05	-7.17E-01	-8.84E-01	4.26E+00	4.26E+00	15.53	16.25	17.51	22.41	20.32	22.19	2	7	5	12	12	13
LAP3	AMPL_HUMAN	P28838	5.20	1.27E+01	1.22E-06	1.22E-06	-7.16E-01	-1.10E+00	5.91E+00	5.91E+00	14.64	15.25	15.46	19.81	20.97	20.17	8	8	11	19	24	21
BRAT1	BRAT1_HUMAN	Q6PJG6	5.20	1.27E+01	1.21E-06	1.21E-06	-7.16E-01	-1.10E+00	5.92E+00	5.92E+00	14.57	13.49	14.04	18.69	19.64	19.36	6	7	7	22	14	26
TMX1	TMX1_HUMAN	Q9H3N1	5.19	1.01E+01	7.26E-06	7.26E-06	-7.15E-01	-1.00E+00	5.14E+00	5.14E+00	14.72	14.79	15.08	19.01	21.10	20.05	2	4	3	9	11	11
PRKDC	PRKDC_HUMAN	P78527	5.17	1.33E+01	8.32E-07	8.32E-07	-7.14E-01	-1.13E+00	6.08E+00	6.08E+00	16.19	17.29	17.24	22.02	21.93	22.29	39	44	48	128	116	133
MTX1	MTX1_HUMAN	Q13505	5.17	1.22E+01	1.69E-06	1.69E-06	-7.14E-01	-1.09E+00	5.77E+00	5.77E+00	12.84	13.25	13.37	19.07	18.29	17.61	2	2	2	8	6	7
ADSS2	PURA2_HUMAN	P30520	5.16	1.30E+01	1.02E-06	1.02E-06	-7.12E-01	-1.11E+00	5.99E+00	5.99E+00	14.21	15.29	14.65	19.64	20.35	19.63	3	5	6	16	10	17
RCN1	RCN1_HUMAN	Q15293	5.14	1.41E+01	5.35E-07	5.35E-07	-7.11E-01	-1.15E+00	6.27E+00	6.27E+00	14.69	15.45	15.48	20.52	19.95	20.56	2	3	4	10	7	11
APMAP	APMAP_HUMAN	Q9HDC9	5.12	1.41E+01	5.41E-07	5.41E-07	-7.10E-01	-1.15E+00	6.27E+00	6.27E+00	13.96	14.38	14.05	18.68	19.68	19.39	2	2	2	14	11	15
TELO2	TELO2_HUMAN	Q9Y4R8	5.12	1.32E+01	9.25E-07	9.25E-07	-7.09E-01	-1.12E+00	6.03E+00	6.03E+00	14.06	14.50	14.12	19.64	18.65	19.76	5	5	5	21	24	20
COX4I1	COX41_HUMAN	P13073	5.11	7.19E+00	8.63E-05	8.63E-05	-7.09E-01	-8.57E-01	4.06E+00	4.06E+00	16.05	14.96	14.30	19.16	21.73	19.77	4	4	2	8	6	9
USP34	UBP34_HUMAN	Q70CQ2	5.04	1.33E+01	2.81E-06	2.81E-06	-7.02E-01	-1.12E+00	5.55E+00	5.55E+00		12.49	13.05	17.66	17.71	18.05	1	3	2	21	6	25
TMX3	TMX3_HUMAN	Q96JJ7	5.01	1.07E+01	3.51E-05	3.51E-05	-7.00E-01	-1.03E+00	4.45E+00	4.45E+00			13.17	18.29	18.07	18.18	0	1	2	8	7	8
PDHX	ODPX_HUMAN	O00330	4.98	1.39E+01	2.04E-06	2.04E-06	-6.98E-01	-1.14E+00	5.69E+00	5.69E+00		13.46	13.25	18.55	18.12	18.34	1	2	2	7	7	8
DNAJC7	DNJC7_HUMAN	Q99615	4.96	1.17E+01	2.32E-06	2.32E-06	-6.95E-01	-1.07E+00	5.63E+00	5.63E+00	14.81	15.58	15.76	20.77	20.58	19.67	4	6	8	28	24	30
XPO5	XPO5_HUMAN	Q9HAV4	4.95	1.00E+01	7.57E-06	7.57E-06	-6.95E-01	-1.00E+00	5.12E+00	5.12E+00	13.47	15.04	14.05	19.48	19.48	18.44	7	7	9	32	24	34
FANCD2	FACD2_HUMAN	Q9BXW9	4.95	1.08E+01	4.11E-06	4.11E-06	-6.94E-01	-1.04E+00	5.39E+00	5.39E+00	13.86	15.49	15.04	19.45	19.92	19.87	8	7	7	27	25	27
SAE1	SAE1_HUMAN	Q9UBE0	4.91	1.30E+01	1.01E-06	1.01E-06	-6.91E-01	-1.11E+00	6.00E+00	6.00E+00	15.64	16.57	16.08	20.83	21.46	20.73	2	4	4	21	16	21
PANK4	PANK4_HUMAN	Q9NVE7	4.89	1.15E+01	7.28E-06	7.28E-06	-6.90E-01	-1.06E+00	5.14E+00	5.14E+00		14.03	13.64	18.26	19.23	18.70	1	4	2	20	9	22
PGRMC1	PGRC1_HUMAN	O00264	4.88	1.19E+01	2.04E-06	2.04E-06	-6.88E-01	-1.07E+00	5.69E+00	5.69E+00	14.61	15.60	15.60	19.76	20.65	20.03	3	3	4	7	10	7
HIP1R	HIP1R_HUMAN	O75146	4.87	7.99E+00	8.40E-05	8.40E-05	-6.88E-01	-9.02E-01	4.08E+00	4.08E+00	13.50		13.06	19.31	17.78	17.37	2	1	2	19	10	19
ZYX	ZYX_HUMAN	Q15942	4.85	7.96E+00	4.15E-05	4.15E-05	-6.86E-01	-9.01E-01	4.38E+00	4.38E+00	16.98	14.49	14.96	20.40	20.24	20.34	10	8	6	14	7	15
PXN	PAXI_HUMAN	P49023	4.82	1.52E+01	3.05E-07	3.05E-07	-6.83E-01	-1.18E+00	6.52E+00	6.52E+00	14.62	15.10	14.89	19.84	19.40	19.85	4	5	5	14	8	14
SLC25A3	S25A3_HUMAN	Q00325	4.81	1.30E+01	1.02E-06	1.02E-06	-6.82E-01	-1.11E+00	5.99E+00	5.99E+00	16.64	17.09	16.66	21.10	22.15	21.56	4	4	4	12	10	11
HLA-C	HLAC_HUMAN	P10321	4.78	8.39E+00	2.84E-05	2.84E-05	-6.79E-01	-9.24E-01	4.55E+00	4.55E+00	14.54	15.13	16.43	19.39	20.86	20.17	4	5	5	8	12	9
AARS1	SYAC_HUMAN	P49588	4.77	1.51E+01	3.06E-07	3.06E-07	-6.78E-01	-1.18E+00	6.51E+00	6.51E+00	15.37	15.44	15.86	20.44	20.44	20.09	11	9	11	32	27	30
PPP2R1B	2AAB_HUMAN	P30154	4.77	1.04E+01	5.70E-06	5.70E-06	-6.78E-01	-1.02E+00	5.24E+00	5.24E+00	14.35	13.03	12.96	18.69	18.05	17.91	2	2	2	11	7	11
UMPS	UMPS_HUMAN	P11172	4.76	6.12E+00	2.66E-04	2.66E-04	-6.77E-01	-7.87E-01	3.57E+00	3.57E+00	13.60	16.32	13.97	18.25	20.13	19.80	4	4	5	21	19	22
ALDH1L2	AL1L2_HUMAN	Q3SY69	4.71	7.96E+00	4.16E-05	4.16E-05	-6.73E-01	-9.01E-01	4.38E+00	4.38E+00	12.35	13.93	12.77	17.08	18.87	17.25	2	5	3	21	12	21
GCN1	GCN1_HUMAN	Q92616	4.71	1.05E+01	5.23E-06	5.23E-06	-6.73E-01	-1.02E+00	5.28E+00	5.28E+00	15.28	16.89	15.86	20.65	20.51	21.01	17	22	21	92	66	100
LMAN2	LMAN2_HUMAN	Q12907	4.69	9.51E+00	1.12E-05	1.12E-05	-6.72E-01	-9.78E-01	4.95E+00	4.95E+00	15.35	15.41	14.99	19.04	20.97	19.81	5	6	6	14	7	14
FAM114A2	F1142_HUMAN	Q9NRY5	4.67	9.01E+00	9.36E-05	9.36E-05	-6.69E-01	-9.55E-01	4.03E+00	4.03E+00	12.46			16.77	17.47	17.14	2	1	1	8	7	9
STAT3	STAT3_HUMAN	P40763	4.65	1.28E+01	1.13E-06	1.13E-06	-6.67E-01	-1.11E+00	5.95E+00	5.95E+00	13.33	12.92	13.88	17.96	17.78	18.32	3	2	2	17	6	19
RARS2	SYRM_HUMAN	Q5T160	4.64	9.79E+00	8.95E-06	8.95E-06	-6.66E-01	-9.91E-01	5.05E+00	5.05E+00	13.27	13.94	14.02	18.72	17.42	18.99	2	3	3	15	19	18
NOTCH2	NOTC2_HUMAN	Q04721	4.60	1.39E+01	5.99E-07	5.99E-07	-6.63E-01	-1.14E+00	6.22E+00	6.22E+00	13.73	13.30	13.96	17.98	18.40	18.40	4	3	3	20	16	22
ADAM9	ADAM9_HUMAN	Q13443	4.57	9.39E+00	7.35E-05	7.35E-05	-6.60E-01	-9.73E-01	4.13E+00	4.13E+00	12.35			16.69	17.14	16.93	2	0	1	10	12	12
HGH1	HGH1_HUMAN	Q9BTY7	4.57	8.28E+00	3.12E-05	3.12E-05	-6.60E-01	-9.18E-01	4.51E+00	4.51E+00	13.50	14.12	14.62	19.18	17.47	19.30	5	2	4	10	7	10
FNDC3B	FND3B_HUMAN	Q53EP0	4.56	6.67E+00	2.64E-04	2.64E-04	-6.59E-01	-8.24E-01	3.58E+00	3.58E+00	13.39	13.88		18.69	16.83	19.08	3	2	1	6	6	6
INF2	INF2_HUMAN	Q27J81	4.56	1.11E+01	3.48E-06	3.48E-06	-6.59E-01	-1.04E+00	5.46E+00	5.46E+00	13.32	14.30	13.32	18.47	17.67	18.47	4	3	4	19	10	18
SPTLC1	SPTC1_HUMAN	O15269	4.56	7.12E+00	1.75E-04	1.75E-04	-6.59E-01	-8.53E-01	3.76E+00	3.76E+00	12.72	13.68		17.19	17.16	18.93	2	3	1	11	8	11
NUP205	NU205_HUMAN	Q92621	4.54	1.30E+01	1.02E-06	1.02E-06	-6.57E-01	-1.11E+00	5.99E+00	5.99E+00	14.08	14.53	14.84	18.66	19.30	19.12	8	9	9	28	18	29
ATP6V1H	VATH_HUMAN	Q9UI12	4.54	1.29E+01	1.11E-06	1.11E-06	-6.57E-01	-1.11E+00	5.96E+00	5.96E+00	14.94	14.28	14.10	18.68	19.21	19.05	3	2	2	8	6	8
AMBRA1	AMRA1_HUMAN	Q9C0C7	4.51	6.44E+00	1.89E-04	1.89E-04	-6.54E-01	-8.09E-01	3.72E+00	3.72E+00	15.27	14.10	14.12	19.51	17.39	20.12	3	5	4	16	8	16
TRAP1	TRAP1_HUMAN	Q12931	4.51	4.44E+00	2.10E-03	2.10E-03	-6.54E-01	-6.47E-01	2.68E+00	2.68E+00	14.57	14.28	15.28	17.20	18.66	21.80	6	6	7	12	7	14
CTPS1	PYRG1_HUMAN	P17812	4.49	1.22E+01	1.63E-06	1.63E-06	-6.52E-01	-1.09E+00	5.79E+00	5.79E+00	15.17	14.93	15.25	19.31	20.24	19.27	8	8	7	25	20	26
IDH3A	IDH3A_HUMAN	P50213	4.48	1.18E+01	2.16E-06	2.16E-06	-6.51E-01	-1.07E+00	5.67E+00	5.67E+00	14.61	15.16	15.79	19.84	19.48	19.68	3	3	5	14	11	14
HOOK1	HOOK1_HUMAN	Q9UJC3	4.47	1.10E+01	3.58E-06	3.58E-06	-6.51E-01	-1.04E+00	5.45E+00	5.45E+00	14.92	13.81	14.13	18.87	18.30	19.13	3	2	4	17	15	17
TRIP13	PCH2_HUMAN	Q15645	4.42	1.40E+01	5.64E-07	5.64E-07	-6.45E-01	-1.15E+00	6.25E+00	6.25E+00	14.83	15.04	15.43	19.45	19.43	19.68	7	7	7	16	9	17
NUP93	NUP93_HUMAN	Q8N1F7	4.41	1.13E+01	2.96E-06	2.96E-06	-6.44E-01	-1.05E+00	5.53E+00	5.53E+00	15.26	15.46	15.68	19.37	20.57	19.69	11	8	7	22	17	23
LMNB1	LMNB1_HUMAN	P20700	4.39	1.33E+01	8.37E-07	8.37E-07	-6.43E-01	-1.12E+00	6.08E+00	6.08E+00	14.32	14.60	14.87	18.65	19.25	19.06	4	4	4	23	15	28
TSC2	TSC2_HUMAN	P49815	4.39	1.36E+01	7.02E-07	7.02E-07	-6.42E-01	-1.13E+00	6.15E+00	6.15E+00	13.29	13.74	13.73	17.81	17.82	18.28	4	5	5	20	14	22
TFRC	TFR1_HUMAN	P02786	4.38	6.43E+00	1.90E-04	1.90E-04	-6.42E-01	-8.08E-01	3.72E+00	3.72E+00	14.55	15.35	17.50	20.15	20.16	20.23	7	8	8	27	17	26
LRPPRC	LPPRC_HUMAN	P42704	4.36	1.31E+01	9.69E-07	9.69E-07	-6.40E-01	-1.12E+00	6.01E+00	6.01E+00	15.59	15.56	16.02	20.19	19.70	20.36	25	26	28	56	43	57
VPS11	VPS11_HUMAN	Q9H270	4.36	1.17E+01	2.32E-06	2.32E-06	-6.40E-01	-1.07E+00	5.64E+00	5.64E+00	13.79	14.02	14.11	18.83	17.71	18.47	2	3	3	20	14	22
PRKAR1A	KAP0_HUMAN	P10644	4.35	7.12E+00	9.26E-05	9.26E-05	-6.38E-01	-8.53E-01	4.03E+00	4.03E+00	13.36	13.97	14.56	19.08	16.94	18.92	2	3	4	9	6	10
ACTR2	ARP2_HUMAN	P61160	4.34	1.04E+01	5.53E-06	5.53E-06	-6.37E-01	-1.02E+00	5.26E+00	5.26E+00	13.96	14.17	14.54	17.88	19.26	18.55	5	4	5	11	7	13
LSS	LSS_HUMAN	P48449	4.31	1.22E+01	1.64E-06	1.64E-06	-6.35E-01	-1.09E+00	5.79E+00	5.79E+00	13.68	14.49	14.14	18.26	18.21	18.79	4	5	5	17	14	19
MON2	MON2_HUMAN	Q7Z3U7	4.31	9.27E+00	1.35E-05	1.35E-05	-6.34E-01	-9.67E-01	4.87E+00	4.87E+00	13.04	14.18	14.69	18.08	18.67	18.09	4	6	6	32	21	31
HSPD1	CH60_HUMAN	P10809	4.27	1.09E+01	4.01E-06	4.01E-06	-6.31E-01	-1.04E+00	5.40E+00	5.40E+00	17.71	17.24	18.38	21.82	22.44	21.88	14	11	15	34	32	34
HECTD1	HECD1_HUMAN	Q9ULT8	4.25	9.96E+00	7.88E-06	7.88E-06	-6.28E-01	-9.98E-01	5.10E+00	5.10E+00	13.75	12.50	13.71	17.63	17.21	17.87	5	5	7	32	18	34
SEC61A1	S61A1_HUMAN	P61619	4.25	4.55E+00	3.70E-03	3.70E-03	-6.28E-01	-6.58E-01	2.43E+00	2.43E+00		14.92		18.26	20.57	18.69	1	2	1	6	7	8

Suppl. Table 3 second part

NUP205	NU205_HUMAN	Q92621	4.54	1.30E+01	1.02E-06	1.02E-06	-6.57E-01	-1.11E+00	5.99E+00	5.99E+00	14.08	14.53	14.84	18.66	19.30	19.12	8	9	9	28	18	29
ATP6V1H	VATH_HUMAN	Q9UI12	4.54	1.29E+01	1.11E-06	1.11E-06	-6.57E-01	-1.11E+00	5.96E+00	5.96E+00	14.94	14.28	14.10	18.68	19.21	19.05	3	2	2	8	6	8
AMBRA1	AMRA1_HUMAN	Q9C0C7	4.51	6.44E+00	1.89E-04	1.89E-04	-6.54E-01	-8.09E-01	3.72E+00	3.72E+00	15.27	14.10	14.12	19.51	17.39	20.12	3	5	4	16	8	16
TRAP1	TRAP1_HUMAN	Q12931	4.51	4.44E+00	2.10E-03	2.10E-03	-6.54E-01	-6.47E-01	2.68E+00	2.68E+00	14.57	14.28	15.28	17.20	18.66	21.80	6	6	7	12	7	14
CTPS1	PYRG1_HUMAN	P17812	4.49	1.22E+01	1.63E-06	1.63E-06	-6.52E-01	-1.09E+00	5.79E+00	5.79E+00	15.17	14.93	15.25	19.31	20.24	19.27	8	8	7	25	20	26
IDH3A	IDH3A_HUMAN	P50213	4.48	1.18E+01	2.16E-06	2.16E-06	-6.51E-01	-1.07E+00	5.67E+00	5.67E+00	14.61	15.16	15.79	19.84	19.48	19.68	3	3	5	14	11	14
HOOK1	HOOK1_HUMAN	Q9UJC3	4.47	1.10E+01	3.58E-06	3.58E-06	-6.51E-01	-1.04E+00	5.45E+00	5.45E+00	14.92	13.81	14.13	18.87	18.30	19.13	3	2	4	17	15	17
TRIP13	PCH2_HUMAN	Q15645	4.42	1.40E+01	5.64E-07	5.64E-07	-6.45E-01	-1.15E+00	6.25E+00	6.25E+00	14.83	15.04	15.43	19.45	19.43	19.68	7	7	7	16	9	17
NUP93	NUP93_HUMAN	Q8N1F7	4.41	1.13E+01	2.96E-06	2.96E-06	-6.44E-01	-1.05E+00	5.53E+00	5.53E+00	15.26	15.46	15.68	19.37	20.57	19.69	11	8	7	22	17	23
LMNB1	LMNB1_HUMAN	P20700	4.39	1.33E+01	8.37E-07	8.37E-07	-6.43E-01	-1.12E+00	6.08E+00	6.08E+00	14.32	14.60	14.87	18.65	19.25	19.06	4	4	4	23	15	28
TSC2	TSC2_HUMAN	P49815	4.39	1.36E+01	7.02E-07	7.02E-07	-6.42E-01	-1.13E+00	6.15E+00	6.15E+00	13.29	13.74	13.73	17.81	17.82	18.28	4	5	5	20	14	22
TFRC	TFR1_HUMAN	P02786	4.38	6.43E+00	1.90E-04	1.90E-04	-6.42E-01	-8.08E-01	3.72E+00	3.72E+00	14.55	15.35	17.50	20.15	20.16	20.23	7	8	8	27	17	26
LRPPRC	LPPRC_HUMAN	P42704	4.36	1.31E+01	9.69E-07	9.69E-07	-6.40E-01	-1.12E+00	6.01E+00	6.01E+00	15.59	15.56	16.02	20.19	19.70	20.36	25	26	28	56	43	57
VPS11	VPS11_HUMAN	Q9H270	4.36	1.17E+01	2.32E-06	2.32E-06	-6.40E-01	-1.07E+00	5.64E+00	5.64E+00	13.79	14.02	14.11	18.83	17.71	18.47	2	3	3	20	14	22
PRKAR1A	KAP0_HUMAN	P10644	4.35	7.12E+00	9.26E-05	9.26E-05	-6.38E-01	-8.53E-01	4.03E+00	4.03E+00	13.36	13.97	14.56	19.08	16.94	18.92	2	3	4	9	6	10
ACTR2	ARP2_HUMAN	P61160	4.34	1.04E+01	5.53E-06	5.53E-06	-6.37E-01	-1.02E+00	5.26E+00	5.26E+00	13.96	14.17	14.54	17.88	19.26	18.55	5	4	5	11	7	13
LSS	LSS_HUMAN	P48449	4.31	1.22E+01	1.64E-06	1.64E-06	-6.35E-01	-1.09E+00	5.79E+00	5.79E+00	13.68	14.49	14.14	18.26	18.21	18.79	4	5	5	17	14	19
MON2	MON2_HUMAN	Q7Z3U7	4.31	9.27E+00	1.35E-05	1.35E-05	-6.34E-01	-9.67E-01	4.87E+00	4.87E+00	13.04	14.18	14.69	18.08	18.67	18.09	4	6	6	32	21	31
HSPD1	CH60_HUMAN	P10809	4.27	1.09E+01	4.01E-06	4.01E-06	-6.31E-01	-1.04E+00	5.40E+00	5.40E+00	17.71	17.24	18.38	21.82	22.44	21.88	14	11	15	34	32	34
HECTD1	HECD1_HUMAN	Q9ULT8	4.25	9.96E+00	7.88E-06	7.88E-06	-6.28E-01	-9.98E-01	5.10E+00	5.10E+00	13.75	12.50	13.71	17.63	17.21	17.87	5	5	7	32	18	34
SEC61A1	S61A1_HUMAN	P61619	4.25	4.55E+00	3.70E-03	3.70E-03	-6.28E-01	-6.58E-01	2.43E+00	2.43E+00		14.92		18.26	20.57	18.69	1	2	1	6	7	8
TNPO1	TNPO1_HUMAN	Q92973	4.23	9.06E+00	1.60E-05	1.60E-05	-6.26E-01	-9.57E-01	4.80E+00	4.80E+00	15.43	15.89	16.49	19.44	20.92	20.13	8	8	7	18	12	18
AHSA1	AHSA1_HUMAN	O95433	4.23	1.30E+01	1.01E-06	1.01E-06	-6.26E-01	-1.11E+00	5.99E+00	5.99E+00	14.81	14.83	15.41	19.03	19.33	19.36	2	5	3	13	11	15
GPSM1	GPSM1_HUMAN	Q86YR5	4.22	1.28E+01	1.16E-06	1.16E-06	-6.26E-01	-1.11E+00	5.93E+00	5.93E+00	14.92	15.25	15.62	19.72	19.29	19.45	4	8	8	24	17	23
LIMD1	LIMD1_HUMAN	Q9UGP4	4.21	1.35E+01	7.68E-07	7.68E-07	-6.24E-01	-1.13E+00	6.11E+00	6.11E+00	14.35	14.65	14.85	18.63	18.82	19.02	3	3	3	10	6	10
MRI1	MTNA_HUMAN	Q9BV20	4.20	6.59E+00	1.61E-04	1.61E-04	-6.23E-01	-8.19E-01	3.79E+00	3.79E+00	14.06	14.60	15.54	18.26	20.31	18.23	2	3	2	8	7	8
RAB6A	RAB6A_HUMAN	P20340	4.19	1.24E+01	1.43E-06	1.43E-06	-6.22E-01	-1.09E+00	5.84E+00	5.84E+00	14.92	15.10	15.19	18.89	19.16	19.72	2	3	2	12	9	12
DCUN1D5	DCNL5_HUMAN	Q9BTE7	4.19	1.14E+01	7.74E-06	7.74E-06	-6.22E-01	-1.06E+00	5.11E+00	5.11E+00	13.96		13.42	17.85	17.82	17.96	2	1	2	9	6	9
ERCC6L	ERC6L_HUMAN	Q2NKX8	4.18	1.04E+01	5.59E-06	5.59E-06	-6.21E-01	-1.02E+00	5.25E+00	5.25E+00	13.70	14.08	14.42	18.63	17.56	18.55	5	3	5	10	6	14
APOL2	APOL2_HUMAN	Q9BQE5	4.12	9.56E+00	2.58E-05	2.58E-05	-6.15E-01	-9.80E-01	4.59E+00	4.59E+00		13.68	14.24	17.58	18.51	18.16	1	3	2	6	6	6
RAB3GAP1	RB3GP_HUMAN	Q15042	4.12	1.37E+01	6.71E-07	6.71E-07	-6.15E-01	-1.14E+00	6.17E+00	6.17E+00	13.57	13.69	13.62	17.52	17.98	17.73	5	6	4	19	15	19
HMOX2	HMOX2_HUMAN	P30519	4.11	1.03E+01	6.29E-06	6.29E-06	-6.14E-01	-1.01E+00	5.20E+00	5.20E+00	14.80	15.39	15.94	19.47	19.10	19.91	4	7	7	10	10	13
TES	TES_HUMAN	Q9UGI8	4.11	1.39E+01	5.90E-07	5.90E-07	-6.14E-01	-1.14E+00	6.23E+00	6.23E+00	14.51	14.83	14.72	18.76	18.71	18.92	7	7	7	18	16	20
ANKLE2	ANKL2_HUMAN	Q86XL3	4.10	1.30E+01	1.03E-06	1.03E-06	-6.12E-01	-1.11E+00	5.99E+00	5.99E+00	14.46	14.70	14.59	18.66	18.37	19.00	5	8	7	21	16	23
CDK1	CDK1_HUMAN	P06493	4.09	9.49E+00	1.14E-05	1.14E-05	-6.12E-01	-9.77E-01	4.94E+00	4.94E+00	16.47	17.20	16.01	20.65	21.18	20.13	6	6	5	16	11	17
IPO8	IPO8_HUMAN	O15397	4.08	1.04E+01	5.70E-06	5.70E-06	-6.10E-01	-1.02E+00	5.24E+00	5.24E+00	14.73	14.17	15.46	18.77	19.03	18.79	3	5	5	8	6	9
CYP51A1	CP51A_HUMAN	Q16850	4.07	7.85E+00	9.42E-05	9.42E-05	-6.10E-01	-8.95E-01	4.03E+00	4.03E+00	13.79	14.33		17.76	19.01	17.63	2	2	1	13	8	14
CLPB	CLPB_HUMAN	Q9H078	4.07	8.92E+00	1.79E-05	1.79E-05	-6.10E-01	-9.51E-01	4.75E+00	4.75E+00	15.06	15.75	15.22	18.61	20.22	19.42	4	7	6	22	16	23
BTAF1	BTAF1_HUMAN	O14981	4.07	6.82E+00	1.26E-04	1.26E-04	-6.10E-01	-8.34E-01	3.90E+00	3.90E+00	13.20	15.09	13.80	18.33	17.15	18.82	7	11	8	27	11	26
RAB18	RAB18_HUMAN	Q9NP72	4.07	1.20E+01	1.89E-06	1.89E-06	-6.10E-01	-1.08E+00	5.72E+00	5.72E+00	14.89	14.86	14.95	18.59	18.85	19.47	3	4	3	9	7	11
XPO6	XPO6_HUMAN	Q96QU8	4.07	1.08E+01	1.13E-05	1.13E-05	-6.09E-01	-1.03E+00	4.95E+00	4.95E+00		13.10	13.24	17.43	16.86	17.41	1	2	2	9	6	11
SDF4	CAB45_HUMAN	Q9BRK5	4.07	7.29E+00	3.10E-04	3.10E-04	-6.09E-01	-8.63E-01	3.51E+00	3.51E+00	13.74			17.96	17.28	18.18	2	1	1	8	6	8
ECPAS	ECM29_HUMAN	Q5VYK3	4.06	1.06E+01	5.00E-06	5.00E-06	-6.08E-01	-1.02E+00	5.30E+00	5.30E+00	15.37	16.07	15.84	19.69	19.37	20.40	13	15	14	38	25	38
IPO9	IPO9_HUMAN	Q96P70	4.05	1.08E+01	4.37E-06	4.37E-06	-6.08E-01	-1.03E+00	5.36E+00	5.36E+00	13.95	15.07	14.49	18.60	18.30	18.76	4	6	5	15	12	16
B4GAT1	B4GA1_HUMAN	O43505	4.04	1.06E+01	5.05E-06	5.05E-06	-6.06E-01	-1.02E+00	5.30E+00	5.30E+00	14.56	15.58	15.38	18.84	19.42	19.39	2	2	2	11	9	13
TBC1D15	TBC15_HUMAN	Q8TC07	4.03	1.20E+01	1.92E-06	1.92E-06	-6.05E-01	-1.08E+00	5.72E+00	5.72E+00	14.38	14.51	15.16	18.54	18.83	18.77	5	6	5	15	11	15
APP	A4_HUMAN	P05067	4.03	8.02E+00	1.82E-04	1.82E-04	-6.05E-01	-9.04E-01	3.74E+00	3.74E+00		13.47		17.70	17.65	17.17	1	4	1	14	6	13
ITGB1	ITB1_HUMAN	P05556	4.02	6.12E+00	8.05E-04	8.05E-04	-6.04E-01	-7.87E-01	3.09E+00	3.09E+00			13.60	17.60	16.91	18.32	1	1	2	12	8	13
ARFGEF3	BIG3_HUMAN	Q5TH69	4.00	1.02E+01	6.75E-06	6.75E-06	-6.03E-01	-1.01E+00	5.17E+00	5.17E+00	12.94	13.29	12.81	16.81	16.53	17.72	2	3	4	20	11	21
XPOT	XPOT_HUMAN	O43592	4.00	1.06E+01	4.82E-06	4.82E-06	-6.02E-01	-1.03E+00	5.32E+00	5.32E+00	13.80	14.53	14.72	18.77	18.18	18.10	4	7	6	25	14	25
VAT1	VAT1_HUMAN	Q99536	n.a.	n.a.	n.a.	n.a.	n.a.	n.a.	n.a.	n.a.	n.a.	n.a.	n.a.	19.85	20.89	19.31	1	1	1	11	10	11
TTC1	TTC1_HUMAN	Q99614	n.a.	n.a.	n.a.	n.a.	n.a.	n.a.	n.a.	n.a.	n.a.	n.a.	n.a.	19.55	20.03	19.83	1	1	1	10	9	10
TMEM106	T106B_HUMAN	Q9NUM4	n.a.	n.a.	n.a.	n.a.	n.a.	n.a.	n.a.	n.a.	n.a.	n.a.	n.a.	19.16	17.93	19.63	1	1	1	7	7	7
NUP107	NU107_HUMAN	P57740	n.a.	n.a.	n.a.	n.a.	n.a.	n.a.	n.a.	n.a.	n.a.	n.a.	n.a.	18.54	18.58	18.74	1	1	1	8	11	9
SYNRG	SYNRG_HUMAN	Q9UMZ2	n.a.	n.a.	n.a.	n.a.	n.a.	n.a.	n.a.	n.a.	n.a.	n.a.	n.a.	18.42	16.13	17.85	1	0	1	12	11	12
BUB1	BUB1_HUMAN	O43683	n.a.	n.a.	n.a.	n.a.	n.a.	n.a.	n.a.	n.a.	n.a.	n.a.	n.a.	18.19	19.75	19.18	1	1	1	14	6	15
STX17	STX17_HUMAN	P56962	n.a.	n.a.	n.a.	n.a.	n.a.	n.a.	n.a.	n.a.	n.a.	n.a.	n.a.	18.10	19.27	18.09	0	0	0	9	10	8
CPT1A	CPT1A_HUMAN	P50416	n.a.	n.a.	n.a.	n.a.	n.a.	n.a.	n.a.	n.a.	n.a.	n.a.	n.a.	18.01	18.16	18.60	1	1	0	15	9	13
ECHDC1	ECHD1_HUMAN	Q9NTX5	n.a.	n.a.	n.a.	n.a.	n.a.	n.a.	n.a.	n.a.	n.a.	n.a.	n.a.	17.85	17.88	17.97	1	1	1	8	6	8
BZW1	5MP2_HUMAN	Q7L1Q6	n.a.	n.a.	n.a.	n.a.	n.a.	n.a.	n.a.	n.a.	n.a.	n.a.	n.a.	17.81	20.08	18.08	1	0	0	11	11	11
FSTL1	FSTL1_HUMAN	Q12841	n.a.	n.a.	n.a.	n.a.	n.a.	n.a.	n.a.	n.a.	n.a.	n.a.	n.a.	17.80	17.92	18.02	1	1	1	7	6	6
COG3	COG3_HUMAN	Q96JB2	n.a.	n.a.	n.a.	n.a.	n.a.	n.a.	n.a.	n.a.	n.a.	n.a.	n.a.	17.54	17.07	17.47	1	0	0	18	8	18
EPHX1	HYEP_HUMAN	P07099	n.a.	n.a.	n.a.	n.a.	n.a.	n.a.	n.a.	n.a.	n.a.	n.a.	n.a.	17.53	17.33	16.73	0	0	0	9	7	11
NR3C1	GCR_HUMAN	P04150	n.a.	n.a.	n.a.	n.a.	n.a.	n.a.	n.a.	n.a.	n.a.	n.a.	n.a.	17.51	17.19	16.49	0	1	1	11	7	10
PCDH9	PCDH9_HUMAN	Q9HC56	n.a.	n.a.	n.a.	n.a.	n.a.	n.a.	n.a.	n.a.	n.a.	n.a.	n.a.	17.28	17.78	17.28	0	1	0	9	7	9
GPD1L	GPD1L_HUMAN	Q8N335	n.a.	n.a.	n.a.	n.a.	n.a.	n.a.	n.a.	n.a.	n.a.	n.a.	n.a.	17.27	18.41	17.47	1	1	1	11	8	12
FIGNL1	FIGL1_HUMAN	Q6PIW4	n.a.	n.a.	n.a.	n.a.	n.a.	n.a.	n.a.	n.a.	n.a.	n.a.	n.a.	17.25	17.24	17.91	1	0	0	17	9	15
DNAJB12	DJB12_HUMAN	Q9NXW2	n.a.	n.a.	n.a.	n.a.	n.a.	n.a.	n.a.	n.a.	n.a.	n.a.	n.a.	17.16	17.89	17.45	1	1	1	6	9	6
WDTC1	WDTC1_HUMAN	Q8N5D0	n.a.	n.a.	n.a.	n.a.	n.a.	n.a.	n.a.	n.a.	n.a.	n.a.	n.a.	17.05	15.70	17.72	0	1	1	11	6	10
ALMS1	ALMS1_HUMAN	Q8TCU4	n.a.	n.a.	n.a.	n.a.	n.a.	n.a.	n.a.	n.a.	n.a.	n.a.	n.a.	17.03	15.47	17.34	1	0	0	16	11	15
UNK	UNK_HUMAN	Q9C0B0	n.a.	n.a.	n.a.	n.a.	n.a.	n.a.	n.a.	n.a.	n.a.	n.a.	n.a.	16.84	16.07	16.59	1	0	0	6	6	7
THADA	THADA_HUMAN	Q6YHU6	n.a.	n.a.	n.a.	n.a.	n.a.	n.a.	n.a.	n.a.	n.a.	n.a.	n.a.	16.81	17.93	16.56	1	1	1	13	7	13
IPO13	IPO13_HUMAN	O94829	n.a.	n.a.	n.a.	n.a.	n.a.	n.a.	n.a.	n.a.	n.a.	n.a.	n.a.	16.79	15.92	16.08	1	1	1	7	7	7
GARRE1	GRRE1_HUMAN	O15063	n.a.	n.a.	n.a.	n.a.	n.a.	n.a.	n.a.	n.a.	n.a.	n.a.	n.a.	16.71	16.55	17.14	1	0	1	16	6	15
PRPF39	PRP39_HUMAN	Q86UA1	n.a.	n.a.	n.a.	n.a.	n.a.	n.a.	n.a.	n.a.	n.a.	n.a.	n.a.	16.58	18.10	16.59	0	1	1	18	11	21
HEATR5B	HTR5B_HUMAN	Q9P2D3	n.a.	n.a.	n.a.	n.a.	n.a.	n.a.	n.a.	n.a.	n.a.	n.a.	n.a.	16.31	16.89	16.92	0	1	1	16	7	15
TSHZ3	TSH3_HUMAN	Q63HK5	n.a.	n.a.	n.a.	n.a.	n.a.	n.a.	n.a.	n.a.	n.a.	n.a.	n.a.	16.28	16.76	17.11	1	1	1	17	10	19
PPM1B	PPM1B_HUMAN	O75688	n.a.	n.a.	n.a.	n.a.	n.a.	n.a.	n.a.	n.a.	n.a.	n.a.	n.a.	16.26	16.42	16.63	0	0	0	8	6	8
TGFBRAP1	TGFA1_HUMAN	Q8WUH2	n.a.	n.a.	n.a.	n.a.	n.a.	n.a.	n.a.	n.a.	n.a.	n.a.	n.a.	16.14	15.93	16.46	1	1	1	6	6	6
ANKRD13A	AN13A_HUMAN	Q8IZ07	n.a.	n.a.	n.a.	n.a.	n.a.	n.a.	n.a.	n.a.	n.a.	n.a.	n.a.	15.86	17.01	15.29	0	1	1	8	6	9
MTFR2	MTFR2_HUMAN	Q6P444	n.a.	n.a.	n.a.	n.a.	n.a.	n.a.	n.a.	n.a.	n.a.	n.a.	n.a.	15.71	16.28	16.58	1	1	1	9	6	8
SHROOM1	SHRM1_HUMAN	Q2M3G4	n.a.	n.a.	n.a.	n.a.	n.a.	n.a.	n.a.	n.a.	n.a.	n.a.	n.a.	15.53	16.69	15.45	0	0	0	7	6	7
BORA	BORA_HUMAN	Q6PGQ7	n.a.	n.a.	n.a.	n.a.	n.a.	n.a.	n.a.	n.a.	n.a.	n.a.	n.a.	15.53	16.19	15.54	0	0	0	7	7	7
IGF1R	IGF1R_HUMAN	P08069	n.a.	n.a.	n.a.	n.a.	n.a.	n.a.	n.a.	n.a.	n.a.	n.a.	n.a.	15.34	15.48	16.43	1	0	0	12	6	13
ATP7B	ATP7B_HUMAN	P35670	n.a.	n.a.	n.a.	n.a.	n.a.	n.a.	n.a.	n.a.	n.a.	n.a.	n.a.	15.22	16.17	15.58	0	0	0	8	6	8

Suppl. Table 4

CLDN10-GFP-Trap	Biological Process	Molecular Function	Cellular Component
**GO-Term**	GO:0051292	GO:0051879	GO:0070381
**Description**	Nuclear pore complex assembly	Hsp90 protein binding	Endosome to plasma membrane transport vesicle
**Count in Network**	3 of 9	5 of 45	2 of 3
**Strength**	1.69	1.21	1.99
**FDR**	0.0377	0.0222	0.0304
**GO-Term**	GO:0061077	GO:0031072	GO:0030134
**Description**	Chaperone-mediated protein folding	Heat shock protein binding	COPII-coated ER to Golgi transport vesicle
**Count in Network**	6 of 71	10 of 126	6 of 90
**Strength**	1.09	1.06	0.99
**FDR**	0.0101	7.72E-05	0.0044
**GO-Term**	GO:0006606	GO:0031267	GO:0030135
**Description**	Protein import into nucleus	Small GTPase binding	Coated vesicle
**Count in Network**	8 of 115	11 of 273	14 of 307
**Strength**	1.01	0.77	0.82
**FDR**	0.0018	0.0049	9.51E-06
**GO-Term**	GO:0010821	GO:0051020	GO:0034399
**Description**	Regulation of mitochondrion organization	GTPase binding	Nuclear periphery
**Count in Network**	8 of 153	12 of 304	6 of 150
**Strength**	0.88	0.76	0.77
**FDR**	0.0101	0.0029	0.0423
**GO-Term**	GO:0006913	GO:0019899	GO:0030662
**Description**	Nucleocytoplasmic transport	Enzyme binding	Coated vesicle membrane
**Count in Network**	12 of 248	38 of 2084	7 of 193
**Strength**	0.85	0.42	0.72
**FDR**	0.00025	7.20E-05	0.0304

Suppl. Table 5

Verification of CLDN10 interaction partners			
IntAct Database		GFP Trap	
Gene ID	human cell line	pos. CoIP	human cell line
ATE1	HEK293T	no	HEK293T
ECPAS	HCT116	yes	HEK293T
FGF1	HEK293T	no	HEK293T
GOPC	HCT116	no	HEK293T
PANK4	HCT116	yes	HEK293T
PRKCA	HCT116	no	HEK293T
RAB29	HCT116	yes	HEK293T
SRC	HCT116	no	HEK293T
STX17	HCT116	no	HEK293T
TELO2	HCT116	yes	HEK293T
TMEM263	HCT116	no	HEK293T

Suppl. Table 6

		Number of Mismatches					
Nr.	Sequence guide	0	1	2	3	4	5
1	AACAAGCGAGCCCTTCTCCG	1	0	0	1	7	75
2	CTGCAGATGGAGAACCCGGG	1	0	1	9	58	170
3	CCCCCGCACTCCCAACCCCG	1	0	1	16	83	411
4	TGGAAGGTGTCTACCATCGA	1	0	0	0	0	23
5	CTGGACGGTCTGCATCCCCG	1	0	0	3	14	120
6	CCCTAGACTGGACTCCTCTG	1	0	0	2	59	430

Generated with OffSpotter (https://cm.jefferson.edu/Off-Spotter/) using default settings; last 5 bases (3') were set as variable, no mismatches were counted here.
